# Enantioselective Organophotocatalytic α‑Functionalization of Aldehydes with *N*‑Lactam Radicals: A Viable Strategy for the Telescoped Synthesis of Levetiracetam

**DOI:** 10.1021/acs.orglett.5c02365

**Published:** 2025-07-29

**Authors:** Eleonora Colombo, Monica Fiorenza Boselli, Marco Fattalini, Valerio Chiroli, Sergio Rossi, Maurizio Benaglia, Alessandra Puglisi

**Affiliations:** Dipartimento di Chimica, 560025 Università degli Studi di Milano, via Golgi, 19, 20133 Milano, Italy

## Abstract

The mild photocatalytic generation of *N*-lactam radicals is explored for the enantioselective α-functionalization of aldehydes in the presence of a chiral imidazolidinone organocatalyst. The reaction provides the desired products in high yields and up to 92% ee using a structurally simple, low-cost, and easy-to-prepare organocatalyst. The methodology was successfully applied to the telescoped synthesis of a pharmacologically relevant compound, (*S*)-levetiracetam, an antiepileptic drug.

## Graphical abstract

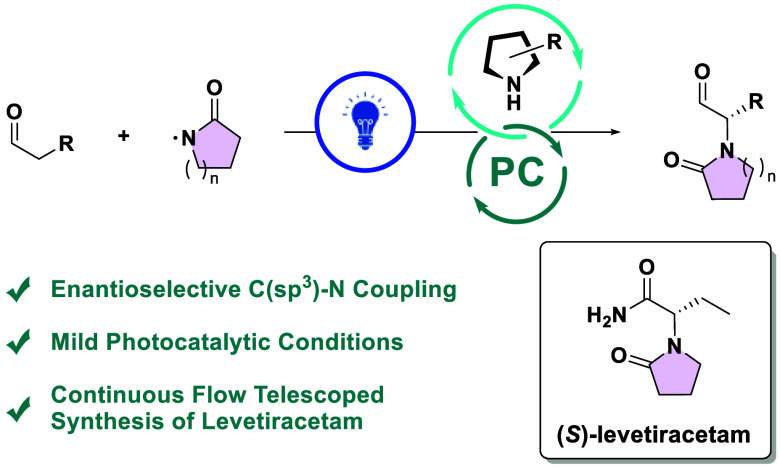

Organic compounds containing nitrogen atoms are commonly found in pharmaceutical and agrochemical products. As a result, developing new and environmentally friendly methods for forming C–N bonds under mild conditions is a key objective in modern chemistry. In this sense, visible light photochemistry^1^ and photocatalytic techniques^2^ have made radical chemistry, especially that involving nitrogen radicals,^3^ a powerful approach for creating innovative synthetic methodologies. Despite the variety of synthetic applications of *N*-centered radicals^4^ developed in the past decade, still very few examples of enantioselective catalytic reactions have been reported. In the pioneering work of 2013, MacMillan published the first asymmetric addition of electrophilic nitrogen radicals to enamines, exploiting the catalysis of imidazolidinones.^5^ Later on, only a few other relevant contributions by other groups, including ours,^6^ appeared.^7^

In this work, we report on the enantioselective α-functionalization of aldehydes exploiting the photoredox generation of *N*-lactam radicals. Lactam rings are essential structural motifs in organic chemistry, widely present in natural products and clinically important drugs, including antibiotics and antiepileptics.^8^ We recently reported a mild and efficient photocatalytic methodology to generate *N*-centered lactam radicals and their reaction with aryl and heteroaryl compounds.^9^ Herein, we report the direct, enantioselective addition of *N*-lactam radicals to aldehydes, leading to an innovative and efficient synthesis of (*S*)-levetiracetam, a clinically important antiepileptic drug.

The reaction between butanal **1a** and radical precursor **2a** ^9,10^ in the presence of organocatalyst **Imi-1** with 4CzIPN as a photocatalyst,^11^ 2,6-lutidine, and 2,6-lutidine triflate in DMA as a solvent under blue light irradiation was chosen as a model reaction to study the stereoselective addition of *N*-lactam radicals in the α-position to aldehyde, as represented in Scheme 1. We first investigated the photoreactor setup. Three different setups, equipped with different light sources and currently used in our laboratories, were tested: a cylinder photoreactor, a Kessil lamp, and a plate photoreactor, developed in the König group^9^ (see the Supporting Information for further details). The three setups allowed us to obtain product **4aa** in 57%, 40%, and 55% yields, respectively; therefore, the plate photoreactor was used for the following screening of the reaction conditions thanks to its ease of use. Given the labile nature of the newly formed stereocenter, aldehyde **3aa** was not isolated but directly reduced with NaBH_4_ in 1:1 EtOH/CH_2_Cl_2_ mixture as a solvent to alcohol **4aa** in 89% ee (determined on the corresponding 2-naphthoyl derivative by HPLC on chiral stationary phase).^5^

**Scheme 1:**
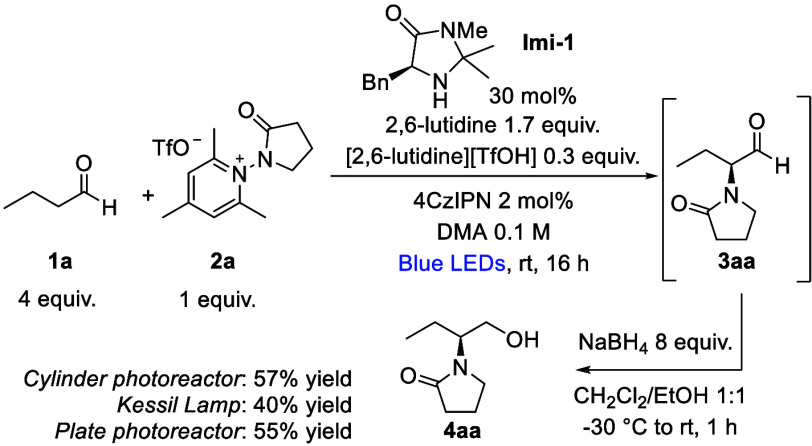
Photoreactor Screening

A small library of organocatalysts, derived from phenylalanine, alanine, and tyrosine, was then synthesized to evaluate the influence of the organocatalyst structure on the ee. In designing the catalyst, we considered the necessity to have a quaternary carbon atom in the C2 position, as already highlighted by MacMillan and co-workers, since the aminal C2 position on the imidazolidinone framework can be susceptible to H atom abstraction by the *N*-centered radical, leading to decreased reaction efficiency.^5^ It was also highly desirable to have a readily available catalyst, easily prepared on a gram scale. The synthesized catalysts and the results in the model reaction are reported in Scheme 2. MacMillan first-generation organocatalyst **Imi-1**, derived from phenylalanine, proved to be the most efficient, leading to the highest yield (55%, isolated yield) and ee (89%) of product **4aa**. On the other hand, more sterically hindered phenylalanine derivative **Imi-2** did not promote the reaction.^12^ Tyrosine derivatives **Imi-6** and **Imi-7**, structurally similar to **Imi-1**, proved to be excellent catalysts, affording the desired product **4aa** in 50% and 45% yields and 89 and 88% ee, respectively. Less satisfactory results, in terms of both yield and ee, were obtained with alanine derivatives **Imi-3–5**.

**Scheme 2:**
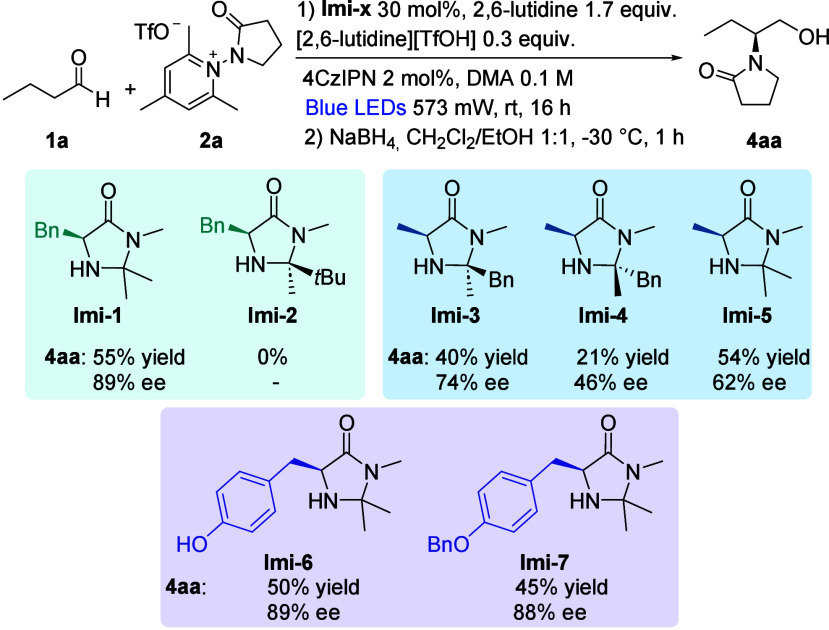
Organocatalyst Screening

We finally investigated the concentration and found that 0.2 M precursor **2a** in DMA was optimal, leading to 70% isolated yield of alcohol **4aa**.

Having in hand the best reaction conditions, we expanded the reaction scope to various aldehydes and *N*-radical precursors of different ring size (Scheme 3).

**Scheme 3:**
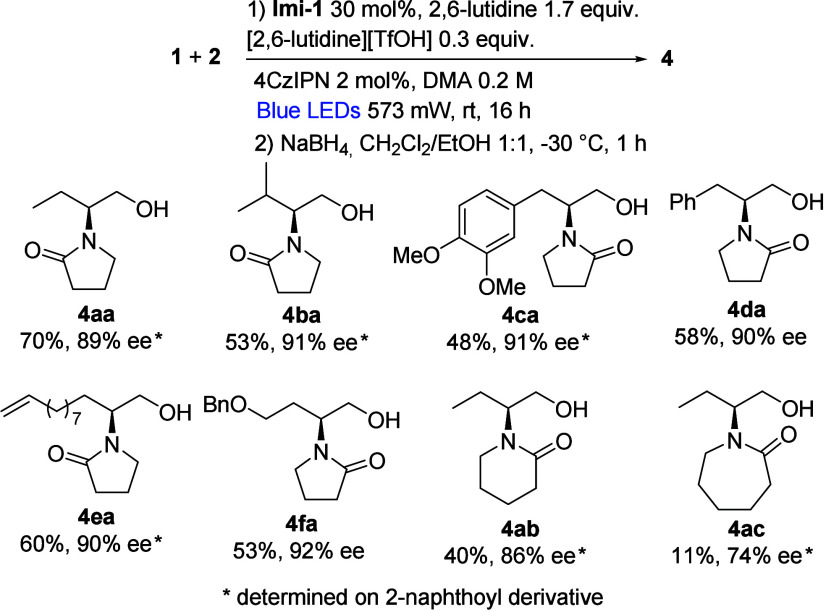
Reaction Scope

The reaction works with different aliphatic aldehydes also bearing aromatic rings (**4ca** and **4da**), a double bond (**4ea**), and protected alcohol (**4fa**); the desired products are isolated in good yields and excellent ee (up to 92%).^13^ The α-functionalization of aldehydes proceeds also with *N*-radical precursors bearing six- and seven-membered rings. It is interesting to notice that the isolated yields of products **4aa**–**4ac** align with the calculated electrophilicity of the corresponding *N*-radicals.^9,14^

By analogy with the literature,^5^ a proposed mechanism for the enantioselective organophotocatalytic α-functionalization of aldehydes with *N*-lactam radicals is depicted in Figure 1. In the photocatalytic cycle, the electrophilic *N*-lactam radical is generated by single-electron transfer (SET) from excited 4CzIPN* to precursor **2**, followed by the release of a molecule of 2,4,6-collidine. The electrophilic *N*-lactam radical rapidly adds to the transiently generated π-rich, chiral enamine, derived from the condensation of imidazolidinone catalyst **Imi-1** with aldehyde coupling partner **1**. Oxidation of the resulting radical species via SET to the radical cation of 4CzIPN (4CzIPN^•+^) closes the photocatalytic cycle, restoring the photocatalyst and delivering the iminium ion. Hydrolysis of the iminium ion reconstitutes imidazolidinone organocatalyst **Imi-1**, leading to the desired enantioenriched α-lactam aldehyde product **3**. This mechanism is supported by the Stern–Volmer experiments, which confirmed that *N*-radical precursor **2a** is a quencher for the excited state of photocatalyst 4CzIPN (see the Supporting Information).

**Figure 1:**
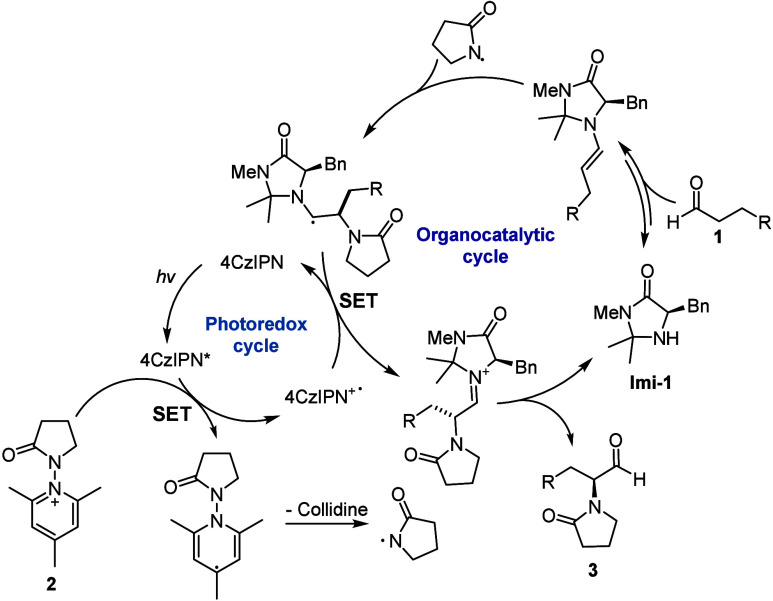
Proposed reaction mechanism for the direct enantioselective photocatalytic α-functionalization of aldehydes with *N*-lactam radicals.

Having in hand a robust methodology, we planned to develop a telescoped synthesis^15^ of (*S*)-levetiracetam, marketed under the name Keppra. It belongs to the broad class of γ-butyrolactams and is a broad-spectrum antiepileptic drug, routinely used as an add-on medication in both children and adults.^16^ Several syntheses have been reported in the literature that can be broadly classified into three main methodologies: chiral pool approach, stereoselective synthesis, and resolution methods. Recent advances in the synthesis of (*S*)-levetiracetam **5** have been thoroughly reviewed;^17^ however, the development of novel stereoselective synthetic routes is strongly encouraged. We reasoned that the direct enantioselective α-functionalization of aldehydes with the *N*-lactam radical could represent a valuable and mild method to prepare **5**. The planned continuous flow synthesis^18^ of **5** is reported in Scheme 4.

**Scheme 4:**
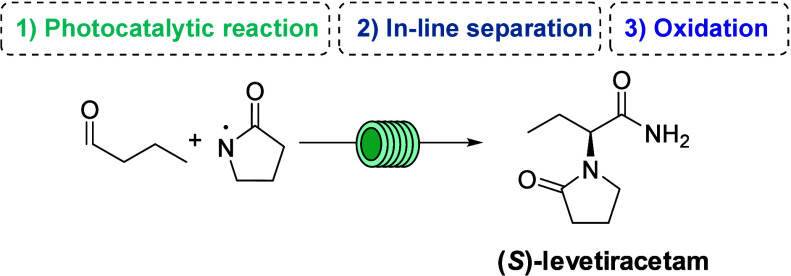
Planned Synthesis of (*S*)-Levetiracetam **5**

We started investigating the optimal reaction conditions for the continuous flow reaction between *N*-lactam radical precursor **2a** and butyraldehyde **1a**. The screening was performed in the Syrris ASIA Premium System, equipped with four 450 nm LEDs in the photochemistry module. After an extensive screening of reaction parameters, we were able to isolate alcohol **4aa** in 45% yield and 89% ee working at 20 °C with 1 mol % 4CzIPN and a 0.2 M solution of precursor **2a** in DMA in a 30 min residence time only. Operating with a 60 min residence time, alcohol **4aa** was isolated in 60% yield; however, a 30 min residence time was selected to provide the best compromise between the overall yield and process productivity. We next developed an in-line removal of unreacted butanal **1a** and a continuous flow, two-step oxidation of aldehyde **3aa** to amide **5** ^19^ (see the Supporting Information for a detailed description).

The final telescoped synthesis is illustrated in Scheme 5. A solution of aldehyde **1a**, precursor **2a**, organocatalyst **Imi-1**, 2,6-lutidine, 2,6-lutidine triflate, and 4CzIPN in DMA was fed into the photochemistry module of an Asia Syrris premium system equipped with 450 nm LEDs, at 20 °C (1 mL, 30 min residence time); the reaction output was mixed with water and hexane in a CSTR and then passed through a SEP-10. The nonpolar phase was discarded, while the polar phase was combined with hexane in a T-junction and passed through a second SEP-10. The resulting DMA/water solution was combined with 30% aqueous NH_3_ and a 1.1 M THF solution of I_2_ and then passed into a first coil (1.3 mL, 15 min); the output and 30% H_2_O_2_ entered a second coil (0.5 mL, 5 min), to complete the oxidation of the aldehyde to the amide, and collected in a flask containing Na_2_SO_3_. After purification by column chromatography, (*S*)-levetiracetam **5** was isolated in 24% overall yield and 82% ee. For the sake of comparison, a batch reaction without product isolation was performed, leading to (*S*)-levetiracetam in 22% yield and 82% ee. We replicated the telescoped synthesis of (*S*)-levetiracetam four times, consistently achieving the same yield and ee, thus assessing the robustness of the process. The continuous flow process allowed us to reach improved productivity (0.1 mmol h^–1^) and space time yield (0.037 mmol h^–1^ mL^–1^) with respect to the batch, proving the superior efficiency of continuous flow technology in light-driven transformations.^20^

**Scheme 5:**
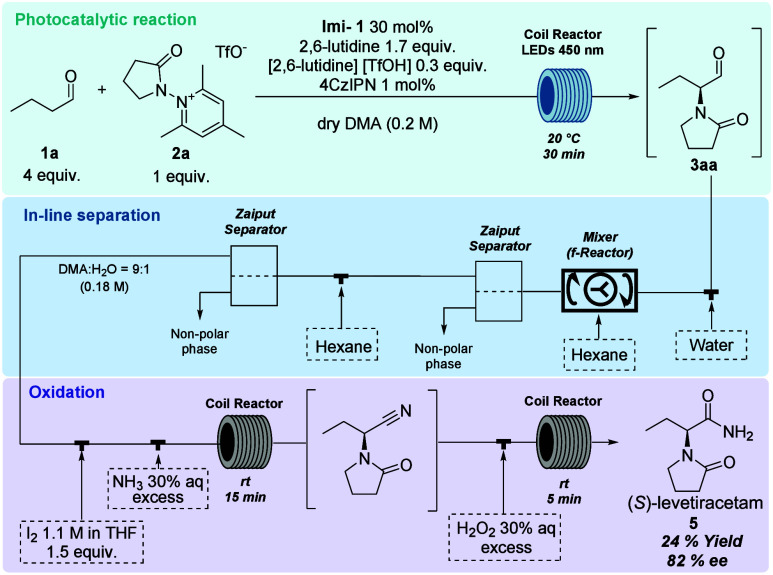
Telescoped Synthesis of (*S*)-Levetiracetam **5**

In conclusion, the direct organophotocatalytic enantioselective addition of *N*-lactam radicals to aldehydes was investigated. The reactivity of an *N*-γ-lactam radical precursor with different aldehydes was studied, leading, under the optimized conditions, to the desired products in good yields and excellent ee using an easily available chiral imidazolidinone as an organocatalyst and 4CzIPN as an organic dye. The reaction can be extended to the use of δ- and ε-lactams. The synthetic methodology was then applied to the telescoped synthesis of (*S*)-levetiracetam, an antiepileptic drug. The overall process, in three operation units, allowed us to isolate (*S*)-levetiracetam in higher productivity and with improved space time yield compared to the in-batch reaction, thus highlighting the benefits of flow technology in light-driven transformations.

## Supplementary Material



## Data Availability

The data underlying this study are available in the published article and its Supporting Information.

## Notes

Experimental procedures and spectroscopic data for the starting material (aldehydes **1** and *N*-radical precursors **2**) and compounds **4** and a detailed description of the equipment used in batch and in flow (PDF)
