# Supplementary material for: Enantioselective Organophotocatalytic α‑Functionalization of Aldehydes with N‑Lactam Radicals: A Viable Strategy for the Telescoped Synthesis of Levetiracetam
Source: Org Lett. 2025 Jul 29;27(31):8540–4. doi: 10.1021/acs.orglett.5c02365 (PMC12340966; doi:10.1021/acs.orglett.5c02365)
Supplement: Supplementary file 1 [file ol5c02365_si_001.pdf]

Supporting Information

Enantioselective Organophotocatalytic α-Functionalization of Aldehydes with *N*-Lactam Radicals: a Viable Strategy for the Telescoped Synthesis of Levetiracetam.

Eleonora Colombo,‡ Monica Fiorenza Boselli,‡ Marco Fattalini, Valerio Chiroli, Sergio Rossi, Maurizio Benaglia, Alessandra Puglisi*

Dipartimento di Chimica, Università degli Studi di Milano, via Golgi, 19 – 20133 Milano - Italy

Table of Contents

1 General methods....4

2 Description of the Photoredox equipment....6

2.1 Plate photoreactor (batch set-up)....6

2.2 Cylinder photoreactor (batch set-up)....6

2.3 Kessil Lamp (batch set-up)....7

2.4 Sublimator photoreactor....7

2.5 Sublimator photoreactor for continuous flow operations **PR-1** (Flow set-up)....8

2.6 Syrris ASIA System **PR-2** (Flow set-up)....8

3 Flow equipment: Specifications of the Coil Reactors....10

4 Synthesis and Characterization of the Substrates....12

4.1 Synthesis of the pyrilium ions....12

4.2 Synthesis of pyridinium ions....13

4.3 Cyclic Voltammetry of the pyridinium ions....19

4.4 Synthesis of the catalysts....22

4.5 Synthesis of Aldehydes starting materials....26

4.6 Synthesis of the racemic 2-(2-Oxo-pyrrolidin-1-yl)-butyraldehyde **3aa**....30

5 Screening of the reaction conditions....31

6 Substrate scope....36

7 Unsuccessful results....41

8 Batch Telescoped Synthesis of Levetiracetam....42

8.1 Approach A....42

8.2 Approach B....43

9 γ-*N* lactam addition to butyraldehyde in flow conditions....44

10 Continuous Flow Telescoped Synthesis of Levetiracetam....45

10.1 Photocatalytic step....45

10.2 Extraction step....45

10.2.1 Batch extraction optimization....46

10.2.2 In-flow extraction optimization....48

10.3 Oxidation step....50

*10.3.1 Aldehyde **3aa** Oxidation to Nitrile under continuous Flow Conditions*....50

*10.3.2 Aldehyde **3aa** Oxidation to Levetiracetam **5** under continuous Flow Conditions*.........................51
10.4 Continuous Flow Telescoped Synthesis of Levetiracetam 53
10.5 Productivity and Space Time Yield Calculations...56
11 Mechanistic Investigations 57
11.1 Stern-Volmer Analysis...57
11.2 ^{1}H-NMR Enamine Formation Studies 58
12 DFT Calculations..60
12.1 Computational details...60
12.2 Optimized Structures, Cartesian Coordinates, Fukui indices, and Energies..........................60
12.3 Electrophilicity Calculation 62
13 NMR Spectra 63
14 HPLC Traces 84
15 References...93

1 General methods

All reactions were carried out under a positive pressure of nitrogen (5 cm of mercury, or with a spring-loaded silicon oil bubbler set to 100 mbar) and dry solvents. If not otherwise stated, reagents were purchased at the highest commercial quality, the solvents were purchased with Across seal and they were used without further purifications. The starting materials, whenever necessary, were synthesized according to the literature procedures. 1,2,3,5-Tetrakis(carbazol-9-yl)-4,6-dicyanobenzene (4CzIPN) was synthesized according to a reported protocol.

Reactions were monitored by thin layer chromatography (TLC) on Macherey-Nagel pre-coated silica gel plates (0.25 mm) and visualized by UV light. Flash chromatography was performed on standard flash column chromatography on Merck silica gel (60, particle size: 0.040–0.063 mm) using petroleum ether or hexane, ethyl acetate (EtOAc), dichloromethane (DCM), methanol (MeOH) as standard solvents.

^{1}H NMR ^{13}C NMR and ^{19}F NMR spectra were recorded on Bruker Avance spectrometers (300 MHz, 75 MHz, 282 MHz, 121 MHz) or (400 MHz, 101 MHz, 376 MHz, 162 MHz) in $CDCl_3$, DMSO-*d6*, MeOH-*d4* and ACN-*d3* solutions with internal solvent signals (for ^{1}H and ^{13}C) as reference (7.26 and 77.2 for $CDCl_3$, 2.50 and 39.5 for DMSO-*d6*, 3.31 and 49.0 for MeOH-*d4*, 1.94 and 1.32 for ACN-*d3*). ^{1}H NMR data are reported as follows: chemical shift (ppm), multiplicity (s = singlet, br. s. = broad singlet, d = doublet, t = triplet, q = quartet, quint = quintet, sext = sextet, hept = heptet, dd = doublet of doublets, ddd = doublet of doublets of doublets, td = triplet of doublets, qd = quartet of doublets, m = multiplet), coupling constants (Hz), and numbers of protons. ^{19}F data are reported as follows: chemical shift (ppm), multiplicity (wherever applicable, s = singlet, d = doublet, t = triplet, q = quartet), coupling constants (Hz), and numbers of fluorine atoms (wherever applicable). Data for ^{13}C NMR are reported in terms of chemical shift, and no special nomenclature is used for equivalent carbons. Structural assignments were made with additional information from gCOSY, gHSQC, gHMBC experiments.

A Spinsolve 80 MHz Benchtop NMR, bought from Magritek, was used to record the NMR spectra during the liquid-liquid extraction studies.

High resolution mass spectra (HRMS) were acquired using a Q-TOF Synapt G2-Si/HDMS 8K instrument available at the MS facility of the Unitech COSPECT at the University of Milan.

Chiral HPLC was measured on an Agilent 1100 or 1200 Serie and they are reported according to the IUPAC recommendations 2013.

Gas chromatography-mass spectrometry (GC-MS) was performed on GC Agilent 6890N, inlet: EPC splitsplitless; column: Agilent 19091S-433 HP-5MS 5% phenyl methyl siloxane; MS: quadrupole G2589A EI; Autosampler: Agilent 7683; gas carrier: helium.

Reaction with microwave apparatus: the reactions were conducted with CEM Discover SP microwave; with an irradiation power of 200 W and reaction temperature of 200 °C for 1 hour with a sealed vessel.

3D-printed photoreactors were 3D-printed with Formlabs “FORM 3” 3D-printer, using Clear V4 or Draft V2 resins.

A Fusion 100-X syringe pump, bought from CHEMYX, has been used to feed reagents in home-made flow setups.

All fluidic connections were made by ¼-28-bore finger tight ferules and adapters (connectors, Y- and T-shape) and were purchased by Cole-Parmer seller.

f-Reactor (Mixer) was purchased from Asynt (internal volume 1.75 mL in absence of stirrer and 1.6 mL in presence of the stirrer).

SEP-10 liquid-liquid separators, as well as the needed inner membranes, were bought from Zaiput Flow Technologies. OB-900-S10 membrane was employed to carry out the desired separation.

UV–Vis measurements were performed with a Cary 4000 spectrometer.

Cyclic voltammetries were carried out under an argon atmosphere. The measurements were performed in respective solvents (DMA) containing 0.1 M tetra-*n*-butylammonium tetrafluoroborate using ferrocene/ferrocenium (Fc/Fc^+) as an internal reference. A glassy carbon electrode (working electrode), platinum wire counter electrode, and Ag quasi-reference electrode were employed.

Melting points were measured using Stuart SMP3 melting point apparatus, provided by STUART SCIENTIFIC.

$[\alpha]_D^T$: Optical rotations were obtained on a JASCO P-1030 polarimeter (Series: A014060839) at 589 nm using a 1 mL cell, with a length of 1 dm.

2 Description of the Photoredox equipment

The Photoredox reactions were performed with three different reaction set-ups in batch and two different set-ups in flow.

2.1 Plate photoreactor (batch set-up)

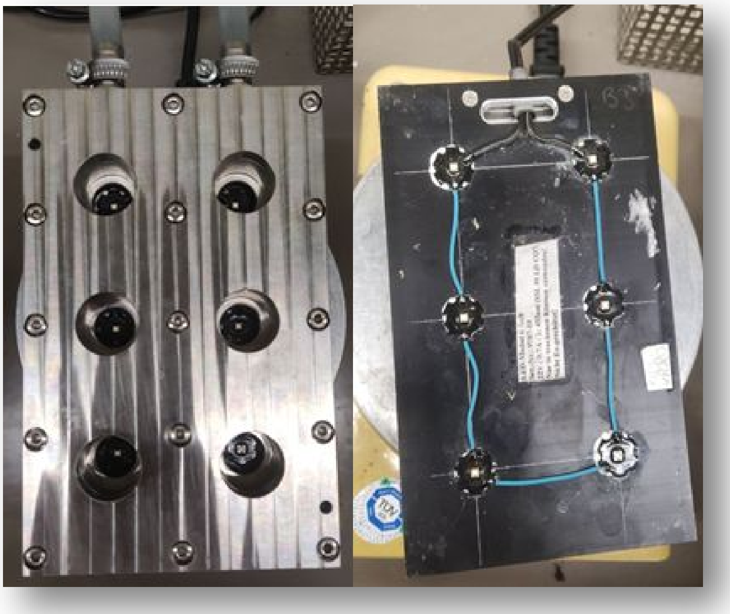

Figure S1 *Image of the plate reactor used in this study.*

The reaction vials for 0.2 – 0.1 mmol scale reactions (5/10/20 mL crimp cap vials) were illuminated from the bottom side with LEDs (in this case with blue LEDs: λ = 455 (± 15) nm, 500 mW – 1.4 W, OSRAM Oslon SSL 80 LDCQ7P-1U3U) and the temperatures were maintained either at 25/60 °C unless noted otherwise from the side using custom-made aluminum cooling blocks connected to a thermostat.

2.2 Cylinder photoreactor (batch set-up)

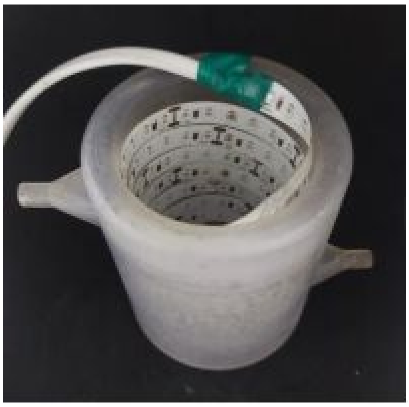

Figure S2 *Image of the cylinder reactor used in this study*

3D-printed, double jacketed cylindrical reaction vessel (11 cm h x 6.5 cm internal diameter). A 100 cm long strip of blue LEDs (540 mW/cm^2, 24 V) has been sticked on the internal wall of the vessel

and the vial of the reaction is placed at the centre of the vessel, 3 cm far from the LEDs. In the external jacket, water flows to cool down the reaction vessel, it is also possible to use compressed air to further cool the reaction vial.
Led-Specifications: Ledpoint **Blu** LED 2835 120 led/m 24 V with a self-adhesive tape that holds the strip light safely and securely to the photoreactor support. The LEDs wavelength emission profile together with their specific light intensity (expressed as mW/cm^2) have been determined using a compact CCD spectrometer (model CCS200/M) connected to a multimode optical fiber, purchased by Thorlabs. Blu LEDs employed are characterized by an almost monochromatic emission profile showing a maximum of intensity located at ca. 460 nm. The light power intensity was thus checked using a Thorlabs PM200 power meter equipped with a S130VC power head with a Si detector. The measured light intensities, though slightly decreasing by moving the maximum of LEDs emission towards longer wavelengths, resulted to be I = 540.2 mW/cm^2.

2.3 Kessil Lamp (batch set-up)

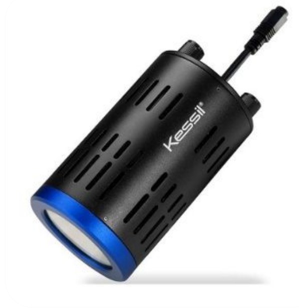

Figure S3 *Image of the cylinder reactor used in this study.*

The reaction vial was positioned 2 cm from the Kessil lamp (456 nm) 100 % of light intensity with an estimated power of 350 mW/cm^2.

2.4 Sublimator photoreactor

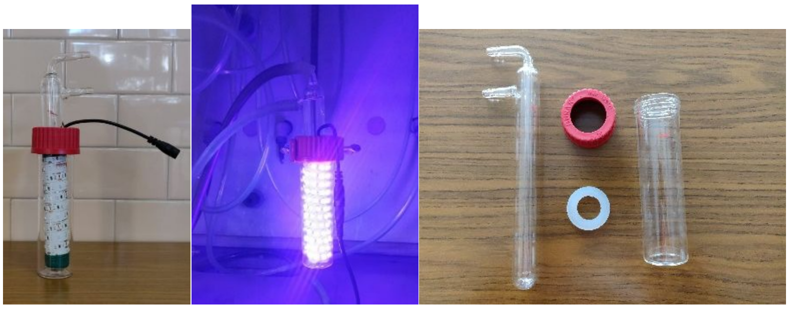

Figure S4 *Image of the water-cooled LED photoreactor used in this study Photograph.*

Construction of the Photoreactor: A making-off video related to the construction of the photoreactor starting from a sublimator apparatus was already reported in literature.[1] The central sublimator glass-piece is first wrapped to the desired length with heavy duty aluminium foil to generate a socket for the LED-strip that possesses high heat conductive properties. Around this first layer is then coiled and glued (double sided adhesive tape) the LED-strip which is further secured in place at the top and bottom with electric isolating tape. The cable is guided through the silicon rubber seal by puncturing it. The final reactor is then assembled as presented in Figure S3.

Led-Specifications: Ledpoint **Blu** LED 2835 120 led/m 24 V with a self-adhesive tape that holds the strip light safely and securely to the photoreactor support. The LEDs wavelength emission profile together with their specific light intensity (expressed as mW/cm^2) have been determined using a compact CCD spectrometer (model CCS200/M) connected to a multimode optical fiber, purchased by Thorlabs. Blu LEDs employed are characterized by an almost monochromatic emission profile showing a maximum of intensity located at ca. 460 nm. The light power intensity was thus checked using a Thorlabs PM200 power meter equipped with a S130VC power head with a Si detector. The measured light intensities, though slightly decreasing by moving the maximum of LEDs emission towards longer wavelengths, resulted to be I = 540.2 mW/cm^2.

2.5 Sublimator photoreactor for continuous flow operations **PR-1** (Flow set-up)

In this photoreactor, the sublimator photoreactor is inserted inside a cylindric 3D printed photoreactor providing a two-sided irradiation of the coil, which is wrapped around the sublimator unit. To further cool the coil reactor, an air vent is placed at the bottom of the continuous flow apparatus (*Figure S4*).

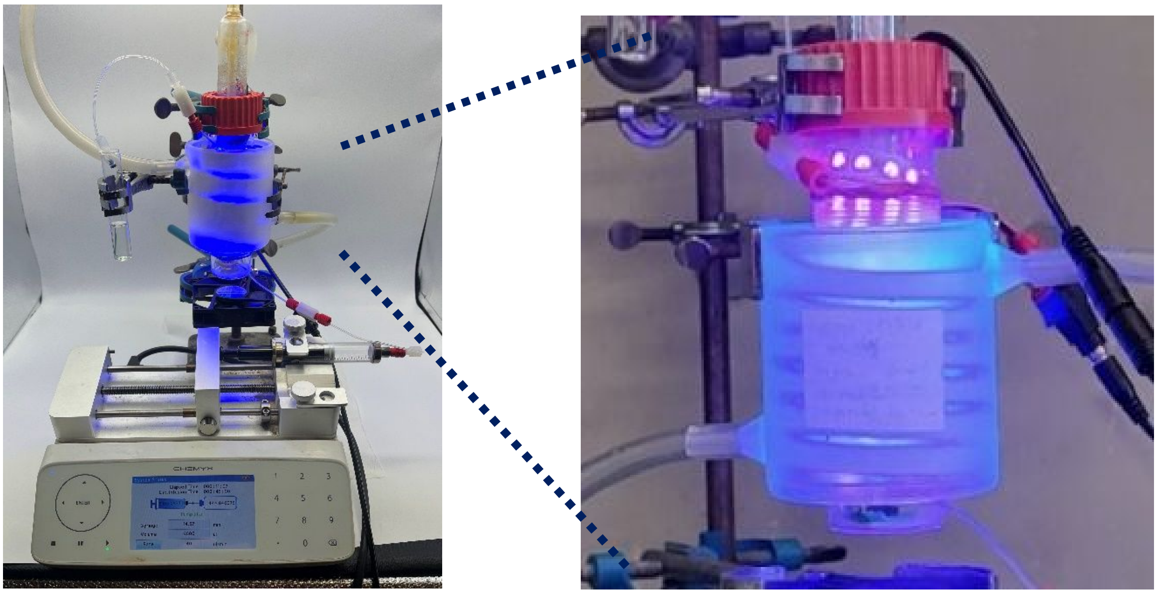

Figure S5 *Photograph of the double photoreactor, composed by the Sublimator reactor inserted inside the cylindrical photoreactor.*

2.6 Syrris ASIA System **PR-2** (Flow set-up)

In this photoreactor, a Syrris ASIA Premium System was employed. The whole continuous-flow systems can be purchased in a modular fashion (Syrris, Asia Ltd) (*Figure S5*).

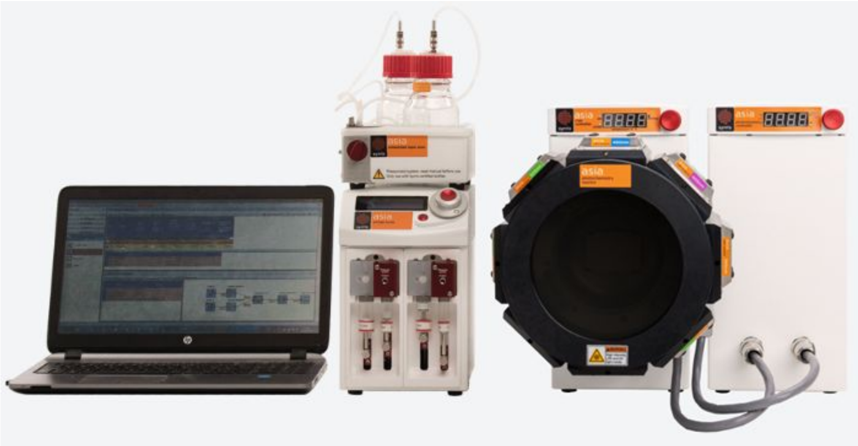

Figure S6 *Syrris ASIA Premium System.*

The liquid feed solution was prepared and stored under inert atmosphere and directly pumped from the volumetric flask using a *Syrris ASIA Syringe Pump*. The solution is pumped in a *Syrris ASIA Tube Reactor*: this system is placed inside a *Syrris ASIA Photochemistry Module*, which is equipped with four 450nm LEDs lamps operating at the maximum possible emitting power. A *Syrris ASIA Heater* allows the controlled heating of the coil reactor at the desired temperature (20 °C). After reaching a steady state, a known amount of the output was collected and analysed.

3 Flow equipment: Specifications of the Coil Reactors

COIL: All fluidic connections were made by ¼-28-bore finger tight ferules and adapters (connectors, Y- and T-shape) and were purchased by Cole-Parmer seller.
To perform the in-flow investigations, different coil reactors have been used. All the coil reactors are made from standard HPLC-Tubing, employing PFA (perfluoroalkoxyalkane) as material of construction.

- Coils employed in the photochemical investigation:

Coil reactor name	Photoreactor	Reactor Volume (µL)	Internal diameter (in)	Reactor Length (cm)
**CR-P-1**	**PR-1**	570	0.02	280
**CR-P-2**	**PR-2**	1000	0.02	510

Table S1 *Specifications for the reactors used for the photochemical studies.*

- Coil employed in the continuous liquid-liquid extractions

Coil reactor name	Reactor Volume (µL)	Internal diameter (in)	Reactor Length (cm)
**CR-E-1**	120 + 60	0.02	60 + 30

Table S2 *Specifications for the reactors used for the in-flow extraction studies.*

- Coils employed in the oxidation investigation:

Oxidation Step	Coil reactor name	Reactor Volume (µL)	Internal diameter (in)	Reactor Length (cm)
**Nitrile**	**CR-O-1**	1360	0.02	670
	**CR-O-2**	1240	0.02	610
**Amide**	**CR-O-4**	1550	0.03	340
	**CR-O-5**	775	0.03	170
	**CR-O-6**	250	0.03	55

Table S3 *Specifications for the reactors used for the In-Flow 2-(2-Oxo-pyrrolidin-1-yl)-butyraldehyde oxidation studies.*

- Coils employed in the Continuous Flow Telescoped Synthesis of Levetiracetam

In the following reaction schemes, it is reported the inner diameter (in blue) and volume (in red) of the various coils that composes the overall setup for the Continuous Flow Telescoped Synthesis of Levetiracetam.

CR-CFS-1

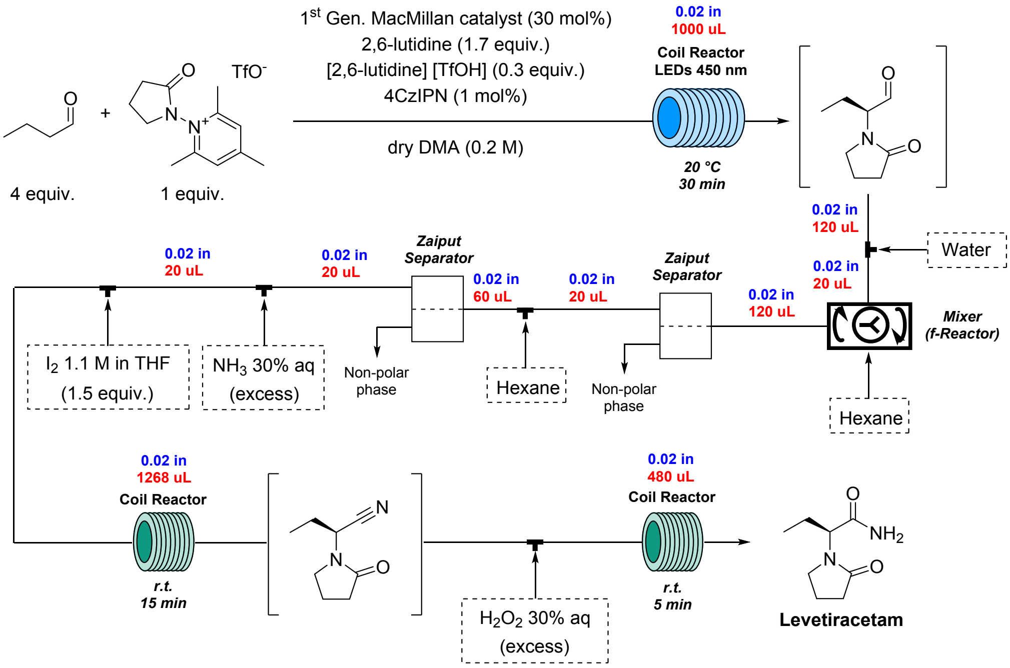

Scheme S1 *Continuous Flow Telescoped Synthesis of Levetiracetam*

4 Synthesis and Characterization of the Substrates

4.1 Synthesis of the pyrilium ions

2,4,6-trimethylpyrylium tetrafluoroborate

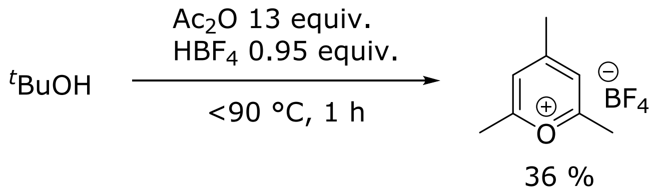

A 250 ml two-necked flask was charged with acetic anhydride (591 mmol, 13 equiv.) and *tert*-butanol (47 mmol, 1 equiv.), providing a colorless solution. HBF_4 (44 mmol, 0.95 equiv.) was introduced dropwise with a dropping funnel: the addition was exothermic, the solution turned yellow at first, and then reddish. After completion of the addition, the reaction mixture has been cooled by dipping the flask in an ice bath: the formation of a white precipitate has been observed. The reaction was quenched by the addition of 10 ml of cold Et_2O. The white precipitate has been recovered by filtration under vacuum, washed with cold Et_2O and dried. The desired product was recovered as a white solid in 36% yield. All the analytical data are in agreement with the literature. [2]

^{1}H NMR (300 MHz, MeOD) δ 7.88 (s, 2H), 2.88 (s, 8H). **^{19}F NMR** (282 MHz, MeOD) δ -154.88.

2,4,6-trimethylpyrylium triflate

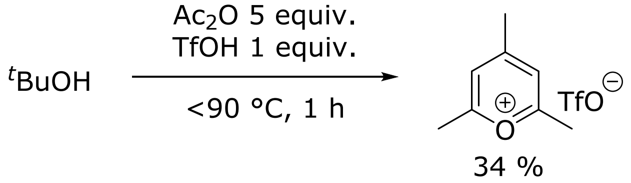

Into a 100 ml round bottom two-necked flask, the acetic anhydride (65 ml, 688 mmol, 5 equiv.) and the ^{t}BuOH (13 ml, 136 mmol, 1 equiv.) were introduced. The addition of the TfOH (11.7 ml, 136 mmol, 1 equiv.) was exothermic and performed with a drip funnel under vigorous stirring controlling with a thermometer that the reaction temperature must be under 90 °C. After the addition, the reaction mixture was slowly cooled down to room temperature, then to 0°C with an ice bath. Et_2O was added and the precipitate was filtered on a Buckner. The desired product was obtained in 45% yield (16.3 g) as a white solid. All the analytical data are in agreement with the literature. [3]

^{1}H NMR (400 MHz, DMSO) δ 7.81 (s, 2H), 2.66 (s, 6H), 2.34 (s, 3H) ppm. **^{19}F NMR** (377 MHz, DMSO) δ -77.33 ppm. **^{13}C NMR** (101 MHz, DMSO) δ 177.8, 173.9, 123.3, 122.7, 119.5, 23.3, 21.3 ppm. **HRMS (ESI + TOF) m/z:** $[M]^+$ Calcd for $C_8H_{11}O$ 123.0804; found 123.0805. **HRMS (ESI - TOF) m/z:** $[M]^-$ Calcd for CF_3O_3S 148.9526; found 148.9529.

4.2 Synthesis of pyridinium ions

4-chloro-*N*'-(diphenylmethylene)butanehydrazide

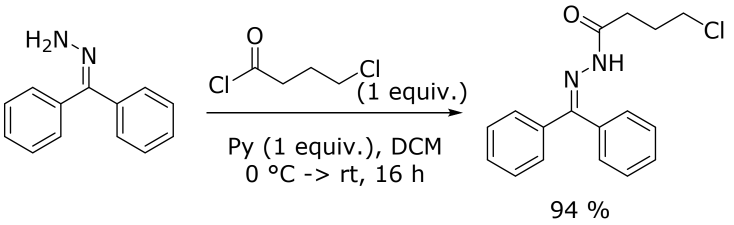

Into a Schlenk 250 ml flask, the (diphenylmethylene)hydrazine (8.8 g, 45 mmol, 1equiv.) was introduced then tree nitrogen-vacuum cycles were done. Dry CH_2Cl_2 (73 ml, 0.62 M) was added, and the reaction mixture was cool down to 0 °C with an ice bath. Pyridine previously distilled (3.7 ml, 45 mmol, 1equiv.) and the 4-chlorobutanoyl chloride (5 ml, 45 mmol, 1 equiv.) were added through syringes. The reaction mixture was stirred overnight with the ice bath slowly warming up under nitrogen atmosphere. After 15 hours, 30 ml of a saturated solution of K_2CO_3 was added and the two phases were separated. The water phase was extracted with CH_2Cl_2 (2 X 40 ml), the combined organic phases were dried out with Na_2SO_4, and the solvent was removed under reduced pressure. The reaction crude was purified by hot crystallization from IPA (40 ml) to provide the desired product as a white solid in 92% yield (12.4 g). All the analytical data are in agreement with the literature. [4]

^{1}H NMR (400 MHz, $CDCl_3$) δ 8.37 (s, 1H), 7.56-7.52 (m, 5H), 7.38-7.33 (m, 3H), 7.25-7.23 (m, 2H), 3.72 (t, *J* = 6.4 Hz, 2H), 3.06 (t, *J* = 7.2 Hz, 2H), 2.25 (p, *J* = 6.8 Hz, 2H).

1-((diphenylmethylene)amino)pyrrolidin-2-one

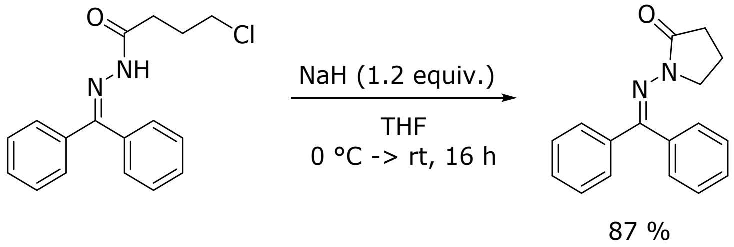

Into a Schlenk 250 ml flask, 2.1 g of NaH 60% in mineral oil (54.2 mmol, 1.2 equiv.) were washed with 10 ml of pentane (3 times). Then, 110 ml of dry THF were added and the 4-chloro-*N*'-(diphenylmethylene)butanehydrazide was added portion wise. The reaction mixture was stirred overnight at room temperature. Then, 50 ml of a saturated solution of NH_4Cl were added and the reaction mixture was stirred for 30 minutes. The phases were separated, and the aqueous phase was extracted with AcOEt (2 x 50 ml). The combined organic phases were dried out with Na_2SO_4, and the solvent was removed under reduced pressure. The reaction crude was purified by hot crystallization from IPA (40 ml) to provide the desired product as a yellow solid in 87 % yield (9.6 g). All the analytical data are in agreement with the literature.[4]

^{1}H NMR (400 MHz, $CDCl_3$) δ 7.61-7.59 (m, 2H), 7.44-7.41 (m, 4H), 7.35-7.30 (m, 4H), 3.32 (t, *J*= 7.0 Hz, 2H), 2.32 (t, *J*= 8.0 Hz, 2H), 1.92 (p, *J*= 7.5 Hz, 2H) ppm.

1-aminopyrrolidin-2-one chlorohydrate

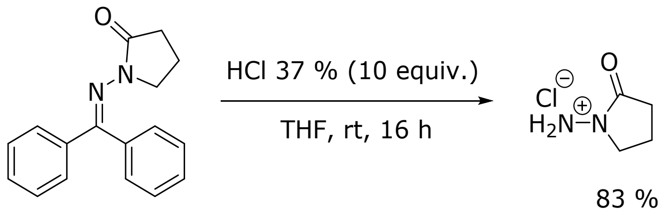

Into a 250 ml 1 neck flask, the 1-((diphenylmethylene)amino)pyrrolidin-2-one was dissolved in 61 ml of THF and then, 30 ml of HCl 37 % were added. The reaction mixture was stirred overnight at room temperature. Then, the THF was removed under reduced pressure. The aqueous phase was extracted with AcOEt (3 x 25 ml) and evaporated under reduced pressure. The reaction crude was purified by hot crystallization from IPA (40 ml) to provide the desired product as a white solid in 83 % yield (3.3 g). All the analytical data are in agreement with the literature.[4]
^{1}H NMR (400 MHz, MeOD) δ 3.65 (t, *J*= 6.91 Hz, 2H), 2.44 (t, *J*= 7.93 Hz, 2H), 2.20 (p, *J*= 7.41 Hz, 2H) ppm.

General procedure A: Synthesis of the pyridinium ions

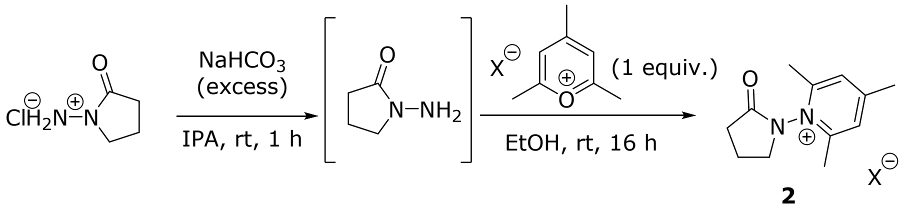

2

1-aminopyrrolidin-2-one chlorohydrate (1 equiv.) was suspended in IPA (0.1 M) and solid $NaHCO_3$ (10 equiv.) was added. The reaction mixture was stirred for 1 hours, and the salts were removed by filtration. The solution was evaporated under reduced pressure obtaining the desired hydrazine as a yellow oil. According to a literature procedure,[2] the hydrazine was dissolved into EtOH (0.3 M) and the pyrylium ion (1 equiv.) was added. The reaction mixture was stirred overnight at room temperature. Then, Et_2O were added the desired product was filtered on a bucker.

2,4,6-trimethyl-1-(2-oxopyrrolidin-1-yl)pyridinium tetrafluoroborate

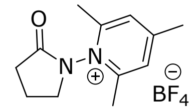

Prepared according to the general procedure A on 20 mmol scale. The desired product was obtained as a white solid in 84 % yield (4.95 g). All the analytical data are in agreement with the literature. [3]
^{1}H NMR (300 MHz, CD_3CN) δ 7.69 (s, 2H), 3.88 (t, *J* = 7.0, 2H), 2.64 (d, *J* = 4.9, 3H), 2.59 (d, *J* = 4.7, 11H), 2.40 (q, *J* = 7.5, 2H) ppm. **^{13}C NMR** (75 MHz, CD_3CN) δ 172.5, 163.1, 157.8, 129.6, 124.2, 48.3, 28.1, 22.2, 19.2, 18.1. **^{19}F NMR** (282 MHz, CD_3CN) δ -152.78 ppm. **HRMS (ESI + TOF) m/z:** $[M]^+$ Calcd for $C_{12}H_{17}N_2O$205.1341; found 205.1341. **M.p.** 154 °C-156 °C.

2,4,6-trimethyl-1-(2-oxopyrrolidin-1-yl)pyridinium triflate (2a)

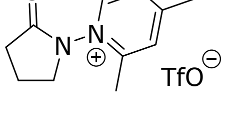

Prepared according to the general procedure A on 1.46 mmol scale. The desired product was obtained as a yellow solid in 55 % yield (287 mg). All the analytical data are in agreement with the literature. [3]
^{1}H NMR (400 MHz, MeOD) δ 7.85 (s, 2H), 4.01 (t, *J* = 7.0 Hz, 2H), 2.76 – 2.66 (m, 8H), 2.64 (s, 3H), 2.46 (p, *J* = 7.5, 2H) ppm. **^{19}F NMR** (376 MHz, MeOD) δ -78.47 ppm. **^{13}C NMR** (101

MHz, MeOD) δ 171.9, 162.3, 156.9, 128.3, 26.9, 20.6, 17.4, 17.0 ppm. **HRMS (ESI + TOF) m/z:** $[M]^+$ Calcd for $C_{12}H_{17}N_2O$ 205.1335; found 205.1336. **HRMS (ESI - TOF) m/z:** $[M]^-$ Calcd for CF_3O_3S 148.9526; found 148.9529. **M.p.** 134 °C-136 °C.

5-chloro-*N*'-(diphenylmethylene)pentanehydrazide

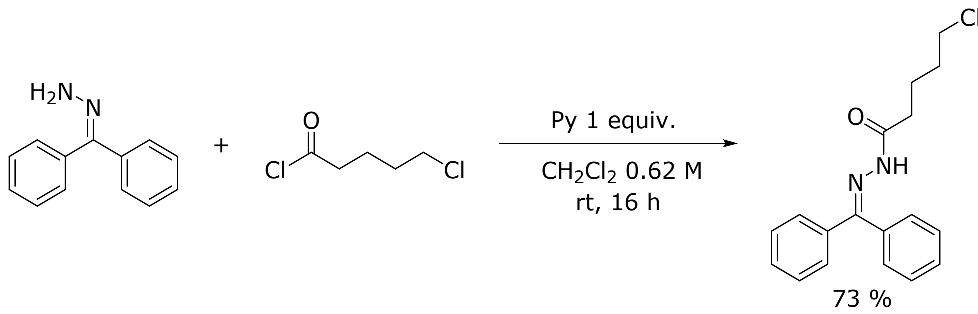

Into a Schlenk 50 ml flask, the (diphenylmethylene)hydrazine (2 g, 10.2 mmol, 1equiv.) was introduced then tree nitrogen-vacuum cycles were done. Dry CH_2Cl_2 (16 ml, 0.62 M) was added, and the reaction mixture was cool down to 0 °C with an ice bath. Pyridine previously distilled (825 µl, 10.2 mmol, 1equiv.) and the 5-chloropentanoyl chloride (1.3 ml, 10.2 mmol, 1 equiv.) were added through syringes. The reaction mixture was stirred overnight with the ice bath slowly warming up. 30 ml of a saturated solution of K_2CO_3 was added and the two phases were separated. The water phase was extracted with CH_2Cl_2 (2 X 40 ml), the combined organic phases were dried out with Na_2SO_4, and the solvent was removed under reduced pressure. The reaction crude was purified by hot crystallization from IPA (40 ml) to provide the desired product as a white solid in 73 % yield (2.34 g). All the analytical data are in agreement with the literature.[3]

^{1}H NMR (400 MHz, $CDCl_3$) δ 8.36 (s, 1H), 7.66 – 7.46 (m, 5H), 7.43 – 7.30 (m, 3H), 7.24 (dd, *J* = 7.8, 1.7 Hz, 2H), 3.67 – 3.56 (m, 2H), 2.94 – 2.83 (m, 2H), 1.92 (m, 4H). **^{13}C NMR** (101 MHz, $CDCl_3$) δ 174.7, 150.4, 137.0, 131.6, 130.0, 129.9, 129.8, 128.54, 128.51, 127.4, 44.8, 32.3, 32.1, 22.0. **HRMS (ESI + TOF) m/z:** $[M + H]^+$ $C_{18}H_{20}ClN_2O$ 315.1259; found 315.1267.

1-((diphenylmethylene)amino)-2-piperidinone

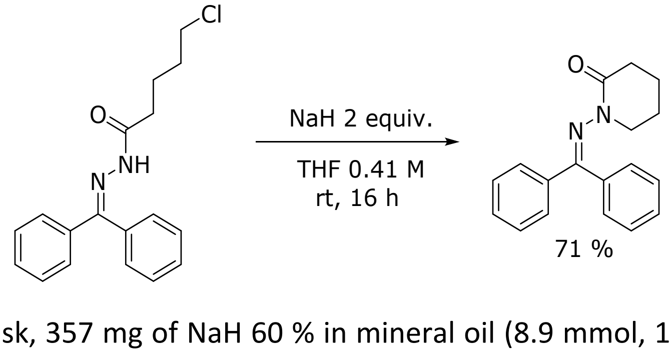

Into a Schlenk 25 ml flask, 357 mg of NaH 60 % in mineral oil (8.9 mmol, 1.2 eq) was washed with 10 ml of pentane (3 times). Then, 19 ml of dry THF were added and the 4-chloro-*N*'-(diphenylmethylene)pentanehydrazide (2.34 g, 1 eq) was added portion wise. The reaction mixture was stirred overnight at room temperature. Then, 50 ml of a saturated solution of NH_4Cl were added and the reaction mixture was stirred for 30 minutes. The phases were separated, and the aqueous phase was extracted with AcOEt (2 x 50 ml). The combined organic phases were dried out with Na_2SO_4, and the solvent was removed under reduced pressure. The reaction crude was purified by

hot crystallization from IPA (40 ml) to provide the desired product as a yellow solid in 71 % yield (1.47 g). All the analytical data are in agreement with the literature.[3]

^{1}H NMR (300 MHz, $CDCl_3$) δ 7.71 – 7.65 (m, 2H), 7.49 – 7.20 (m, 8H), 3.41 (t, *J* = 5.7 Hz, 2H), 2.22 (t, *J* = 6.2 Hz, 2H), 1.76 – 1.58 (m, 2H). **^{13}C NMR** (75 MHz, $CDCl_3$) δ 175.5, 164.4, 136.6, 135.2, 131.2, 129.3, 129.2, 128.1, 127.9, 127.5, 50.3, 32.4, 22.9, 21.0.

1-aminopiperidin-2-one hydrochloride

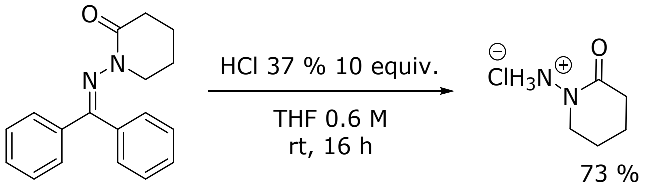

Into a 25 ml 1 neck flask, the 1-((diphenylmethylene)amino)-2-piperidinone (1.47 g, 1 equiv.) was dissolved in 9 ml of THF and then, 5 ml of aqueous HCl 37 % were added. The reaction mixture was stirred overnight at room temperature. Then, the THF was removed under reduced pressure. The aqueous phase was extracted with AcOEt (3 x 25 ml) and evaporated under reduced pressure. The reaction crude was purified by hot crystallization from acetonitrile (10 ml) to provide the desired product as a yellow solid in 73 % yield (573 mg). All the analytical data are in agreement with the literature.[3]

^{1}H NMR (300 MHz, MeOD) δ 5.48 (s, 2H), 3.73 (t, *J* = 5.8 Hz, 2H), 2.53 (t, *J* = 6.4 Hz, 2H), 2.03 (dd, *J* = 5.8, 2.6 Hz, 2H), 1.96 – 1.82 (m, 2H) ppm. **^{13}C NMR** (75 MHz, MeOD) δ 170.6, 50.9, 32.6, 23.7, 21.5 ppm.

2,4,6-trimethyl-1-(2-oxopiperidin-1-yl)pyridinium triflate (2b)

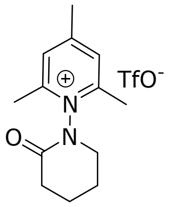

Prepared according to the general procedure A on 3.8 mmol scale. The desired product was obtained as a yellow solid in 60 % yield (825 mg) after purification by flash column chromatography (CH_2Cl_2:MeOH 95:5 -> 85:15). All the analytical data are in agreement with the literature. [3]

^{1}H NMR (300 MHz, MeOD) δ 7.84 (s, 2H), 3.92 (t, *J* = 6.0 Hz, 2H), 2.76 (t, *J* = 6.5 Hz, 2H), 2.68 (s, 6H), 2.64 (s, 3H), 2.27 – 2.13 (m, 2H), 2.06 (m, 2H). **^{19}F NMR** (376 MHz, MeOD) δ -78.49. **^{13}C NMR** (75 MHz, MeOD) δ 167.9, 161.9, 156.1, 128.5, 51.5, 31.7, 22.5, 20.6, 19.8, 17.3. **HRMS (ESI + TOF) m/z:** $[M]^+$ Calcd for $C_{13}H_{19}N_2O$ 219.1497; found 219.1492. **M.p.** 141 °C-143 °C.

6-chloro-*N'*-(diphenylmethylene)esanehydrazide

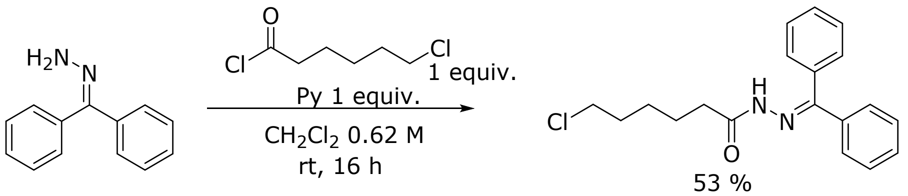

Into a Schlenk 100 ml flask, the (diphenylmethylene)hydrazine (4.6 g, 23.6 mmol, 1 equiv.) was introduced then tree nitrogen-vacuum cycles were done. Dry CH_2Cl_2 (40 ml, 0.62 M) was added, and the reaction mixture was cool down to 0 °C with an ice bath. Pyridine previously distilled (1.9 ml, 23.6 mmol, 1 equiv.) and the 6-chloroesananoyl chloride (4 g, 23.3 mmol, 1 equiv.) were added

through syringes. The reaction mixture was stirred overnight with the ice bath slowly warming up. 30 ml of a saturated solution of K_2CO_3 was added and the two phases were separated. The water phase was extracted with CH_2Cl_2 (2 X 40 ml) and the combined organic phases were dried out with Na_2SO_4 and the solvent was removed under reduced pressure. The reaction crude was purified by hot crystallization from IPA (40 ml) to provide the desired product as a white solid in 58 % yield (4.55 g). All the analytical data are in agreement with the literature.[3]
^{1}H NMR (300 MHz, $CDCl_3$) δ 8.37 (s, 1H), 7.69 – 7.20 (m, 10H), 3.59 (t, *J* = 6.7 Hz, 2H), 2.89 (t, *J* = 7.5 Hz, 2H), 1.85 (dp, *J* = 23.6, 7.3 Hz, 4H), 1.61 (tdd, *J* = 9.5, 6.4, 3.6 Hz, 2H) ppm. **^{13}C NMR** (75 MHz, $CDCl_3$) δ 175.0, 150.2, 137.0, 131.6, 129.9, 129.8, 129.6, 129.3, 128.6, 128.4, 128.4, 128.0, 127.8, 127.3, 44.9, 32.7, 32.4, 26.7, 23.9 ppm.

1-((diphenylmethylene)amino)-2-azepan2-one

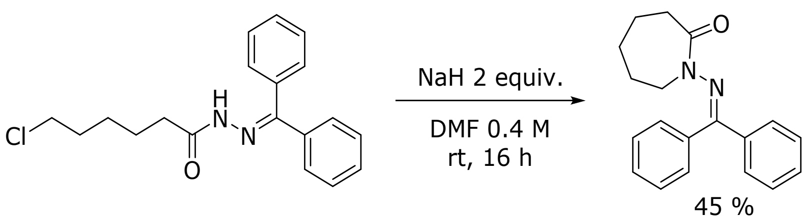

Into a Schlenk 100 ml flask, 740 mg of NaH 60 % in mineral oil (19.3 mmol, 2 equiv.) was washed with 10 ml of pentane (3 times). Then, 24 ml of dry DMF were added and the 6-chloro-*N*'-(diphenylmethylene)esanehydrazide (3.17 g, 1 equiv.) was added portion wise. The reaction mixture was stirred overnight at room temperature. Then, 15 ml of H_2O were added, and the reaction mixture was stirred for 30 minutes. The phases were separated, and the aqueous phase was extracted with AcOEt (2 x 20 ml). The combined organic phases were dried out with Na_2SO_4, and the solvent was removed under reduced pressure. The reaction crude was purified by column chromatography on basic alumina (Hexane-AcOEt 7:3 -> 6:4 -> 1:1) to provide the desired product as a yellow solid in 41 % yield (1.150 g). All the analytical data are in agreement with the literature.[3]
^{1}H NMR (300 MHz, $CDCl_3$) δ 7.68 – 7.59 (m, 2H), 7.41 – 7.18 (m, 8H), 3.59 – 3.48 (m, 2H), 2.40 – 2.30 (m, 2H), 1.68 – 1.41 (m, 6H) ppm. **^{13}C NMR** (75 MHz, $CDCl_3$) δ 173.5, 170.0, 136.8, 136.0, 131.0, 129.7, 129.2, 129.0, 128.9, 128.3, 128.0, 127.89, 127.86, 127.2, 53.0, 36.9, 29.8, 26.9, 22.8 ppm. **HRMS (ESI+ TOF) m/z:** $[M + Na]^+$ Calcd for $C_{19}H_{20}N_2ONa$ 15.1473; found 315.1475.

amino-azepan-2-one hydrochloride

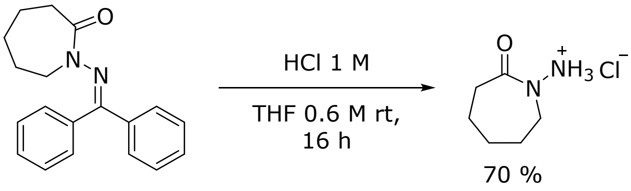

Into a 10 ml 1 neck flask, the 1-((diphenylmethylene)amino)-2-azepan-2-one (380 mg, 1 equiv.) was dissolved in 2 ml of THF and then, 760 µl of HCl 1 M were added. The reaction mixture was stirred overnight at room temperature. Then, the THF was removed under reduced pressure. The aqueous phase was extracted with AcOEt (3 x 25 ml) and evaporated under reduced pressure. The desired product was obtained as a white solid in 70 % yield (150 mg) without purification. All the analytical data are in agreement with the literature. [3]

^{1}H NMR (300 MHz, MeOD) δ 3.76 (dd, *J* = 6.6, 2.8 Hz, 2H), 2.68 – 2.58 (m, 2H), 1.89 – 1.73 (m, 4H), 1.76 – 1.60 (m, 2H) ppm. **^{13}C NMR** (75 MHz, MeOD) δ 175.8, 51.8, 35.8, 30.2, 28.2, 23.8 ppm. **HRMS (ESI+ TOF) m/z:** $[M]^+$ Calcd for $C_6H_{13}N_2O$ 129.1028; found 129.1026.

2,4,6-trimethyl-1-(2-oxoazepan-1-yl)pyridinium triflate (2c)

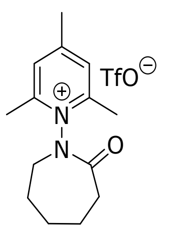

Prepared according to the general procedure A on 0.62 mmol scale. The desired product was obtained as a yellow solid in 52 % yield (123 mg). All the analytical data are in agreement with the literature. [3]

^{1}H NMR (300 MHz, MeOD) δ 7.82 (s, 2H), 4.14 – 4.05 (m, 2H), 2.91 – 2.80 (m, 2H), 2.68 (s, 6H), 2.63 (s, 3H), 2.06 (s, 2H), 1.96 – 1.85 (m, 4H) ppm. **^{13}C NMR** (75 MHz, MeOD) δ 174.8, 162.9, 157.7, 129.8, 124.3, 55.0, 36.7, 30.4, 29.1, 23.3, 21.9, 19.9 ppm. **^{19}F NMR** (76 MHz, MeOD) δ -78.50. **HRMS (ESI+ TOF) m/z:** $[M]^+$ Calcd for $C_{14}H_{21}N_2O$ 233.1654; found 233.1652. **M.p.** 126 °C-128 °C.

4.3 Cyclic Voltammetry of the pyridinium ions

Measurements are carried out with an **Autolab PGSTAT302N Metrohm**. Working electrode: Glassy Carbon. Counter electrode: Platinum wire. Pseudo reference electrode: Silver wire. Supporting electrolyte: Tetrabutylammonium tetrafluoroborate Fluka 0.1 M. Prior to the measurement the DMA is degassed with argon. All experiments are performed under argon atmosphere. Ferrocene is used as an internal reference for determining the reduction and oxidation potentials. The scan rate was 100 mV s^{-1}.

2,4,6-trimethyl-1-(2-oxopyrrolidin-1-yl)pyridinium tetrafluoroborate

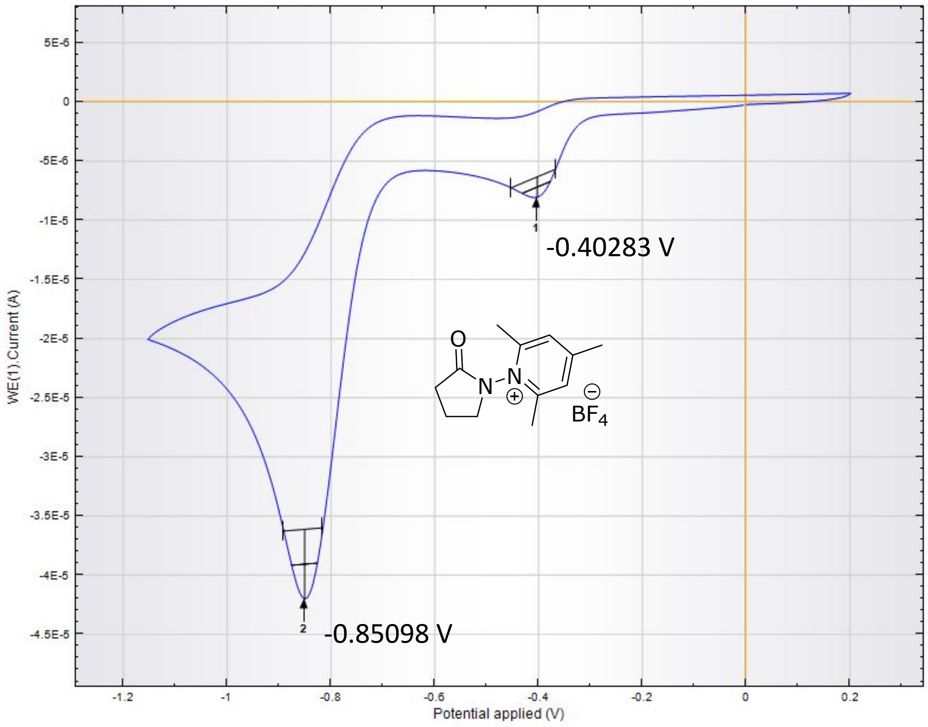

Addition of Ferrocene

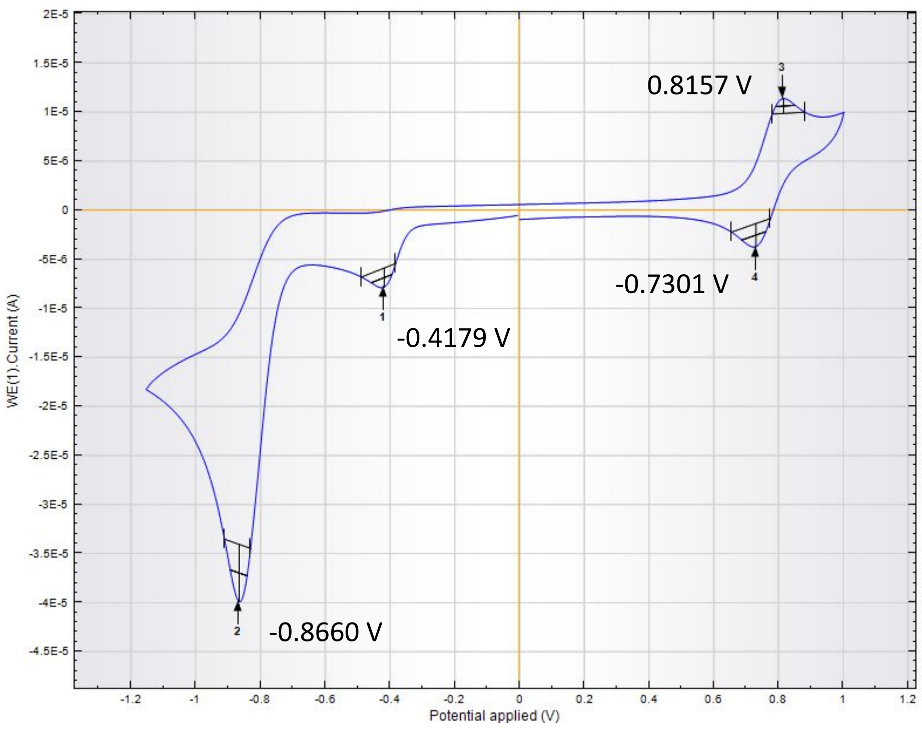

2,4,6-trimethyl-1-(2-oxopyrrolidin-1-yl)pyridinium triflate (2a)

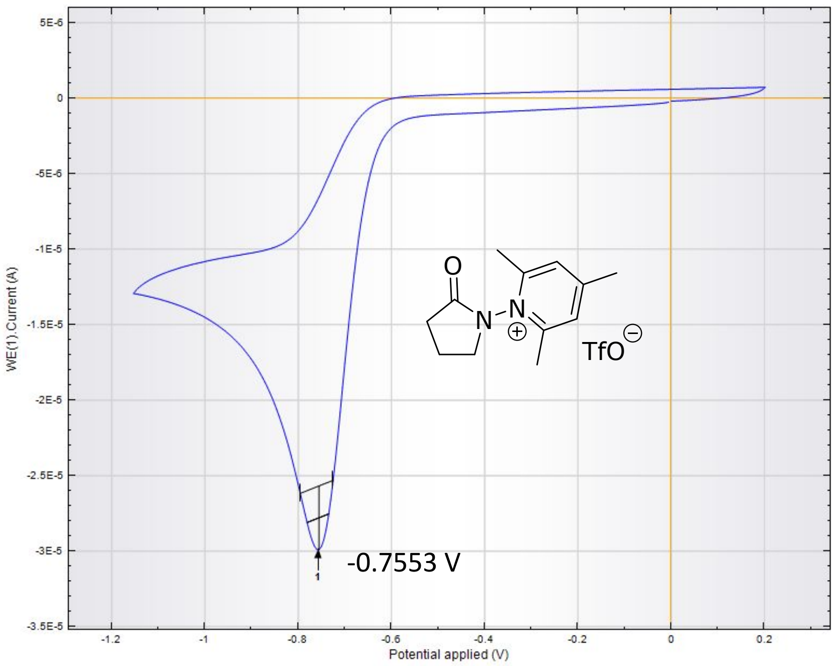

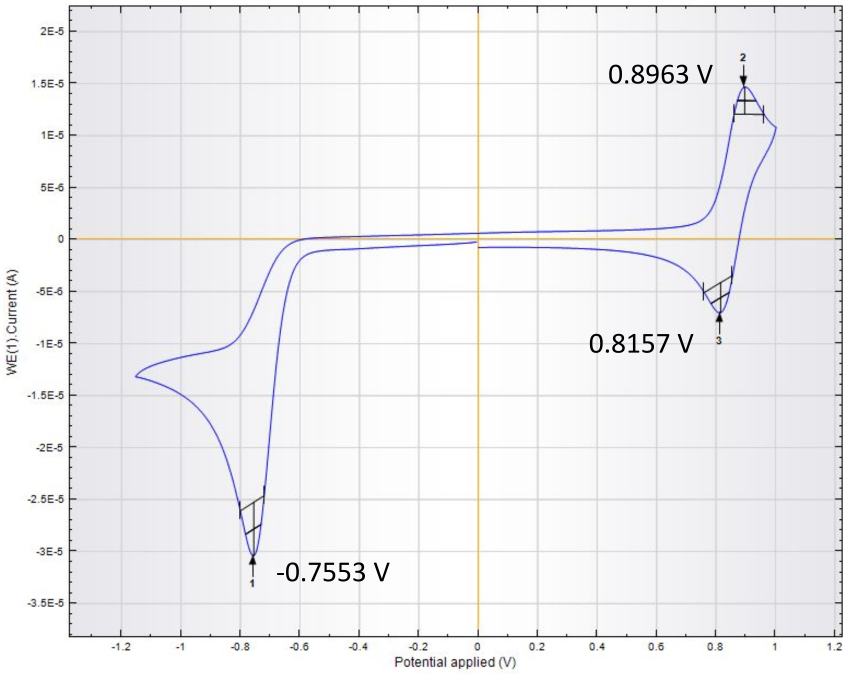

4.4 Synthesis of the catalysts

General Procedure B: Synthesis of the amino amides

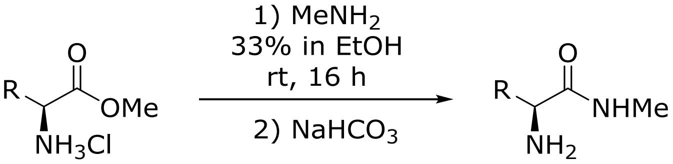

The amino methyl ester hydrochloride (1 equiv.) was dissolved into a methylamine solution (33% in EtOH, 18 equiv.). The reaction mixture was stirred overnight at room temperature. After that time, the volatiles were removed under reduced pressure, toluene (1 M) was added and removed under pressure in order to obtain a white solid. Then, the crude was dissolved into a s.s. $NaHCO_3$ and extracted with CH_2Cl_2 (3 times). The combined organic phases were washed with brine, dried with Na_2SO_4 and the solvent was removed under reduced pressure. The desired products were obtained as yellow oils, without further purification.

(*S*)-2-amino-*N*-methyl-3-phenylpropanamide

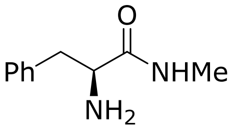

Prepared according to general Procedure B on 7.5 mmol scale. The desired product was obtained as a yellow oil in 78% yield. All the analytical data are in agreement with the literature. [5]

^{1}H NMR (300 MHz, $CDCl_3$) δ 7.30-7.19 (m, 5H), 3.64 (dd, *J* = 9.3, 4.0 Hz, 1H), 3.31 (dd, *J* = 13.7, 4.2 Hz, 1H), 2.83 (d, *J* = 4.9 Hz, 3H), 2.70 (dd, *J* = 13.8, 9.3 Hz, 1H).

2-amino-*N*-methylacetamide

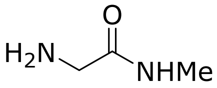

Prepared according to general Procedure B on 22.6 mmol scale.The desired product was obtained as a yellow oil in 99% yield. All the analytical data are in agreement with the literature. [6]

^{1}H NMR (300 MHz, MeOD) δ 3.25 (s, 2H), 2.77 (s, 3H).

(*S*)-2-amino-*N*-methyl-3-methylpropanamide

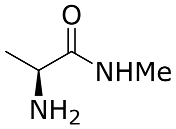

Prepared according to general Procedure B on 5.3 mmol scale. The desired product was obtained as a yellow oil in 99% yield. All the analytical data are in agreement with the literature. [7]

^{1}H NMR (300 MHz, MeOD) δ 3.87 (q, *J* = 7.1 Hz, 1H), 2.79 (s, 3H), 1.48 (d, *J* = 7.1 Hz, 3H).

(*S*)-2-amino-*N*-methyl-3-methylpropanamide

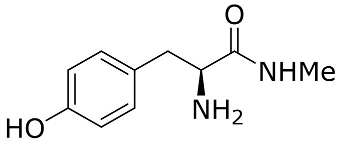

Prepared according to general Procedure B on 4.6 mmol scale. The desired product was obtained as a yellow oil in 94% yield. All the analytical data are in agreement with the literature. [8]

^{1}H NMR (300 MHz, MeOD) δ 7.01 (d, J = 8.4 Hz, 2H), 6.71 (d, J = 8.4 Hz, 2H), 3.50 – 3.41 (m, 1H), 2.88 (dd, J = 13.5, 6.5 Hz, 1H), 2.71 (dd, J = 13.5, 7.2 Hz, 1H), 2.67 (s, 3H).

<u>General Procedure C</u> Synthesis of the imidazolidinones organocatalysts

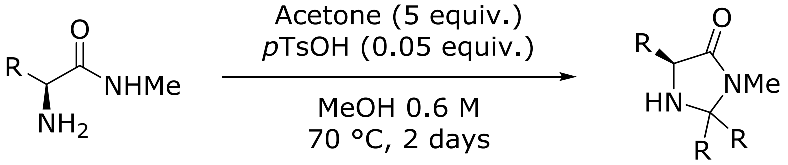

The aminoamides previously obtained (1 equiv.) was introduced into a one-neck flask and dissolved in 10 ml of methanol (1 M), then *p*TsOH (0.05 equiv.) and acetone (5 equiv.) were added. The flask was equipped with a dean-stark apparatus containing molecular sieves, bubble condenser and a nitrogen inlet. The reaction was stirred at reflux with an oil bath for 2 days under nitrogen atmosphere. Then, the solvent was removed under reduced pressure, the product was dissolved in CH_2Cl_2 and a s.s. $NaHCO_3$ were added. The aqueous layer was extracted with 10 mL of CH_2Cl_2 three times. The combined organic phases were dried over Na_2SO_4 and the solvent was removed under reduced pressure. The reaction crude was purified by flash column chromatography on silica gel to provide the desired products as transparent oils.

1st generation MacMillan catalyst (Imi-1)

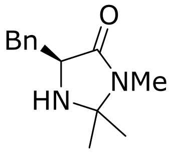

Prepared according to general Procedure C, on 6 mmol scale. The desired product was obtained as a transparent oil in 92% yield (1.18 g) after purification by flash column chromatography (CH_2Cl_2:MeOH 99:1 → 97:3). All the analytical data are in agreement with the literature. [5]

^{1}H NMR (300 MHz, $CDCl_3$) δ 7.35 – 7.19 (m, 5H), 3.80 (dd, *J* = 6.8, 4.5 Hz, 1H), 3.15 (dd, *J* = 14.2, 4.5 Hz, 1H), 3.02 (dd, *J* = 14.1, 6.7 Hz, 1H), 2.76 (s, 3H), 1.69 (bs, 2H), 1.27 (s, 3H), 1.16 (s, 3H).

2,2,3-trimethylimidazolidin-4-one

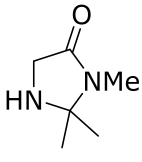

Prepared according to general Procedure C on 23 mmol scale. The desired product was obtained as a transparent oil in 74% yield (2.18 g) after purification by flash column chromatography on silica gel (Hexane-AcOEt 95-5 -> 8-2). All the analytical data are in agreement with the literature. [6]

^{1}H NMR (300 MHz, $CDCl_3$) δ 3.47 (s, 2H), 2.78 (s, 3H), 1.36 (d, *J* = 1.5 Hz, 6H).

2-benzyl-2,3,5-trimethylimidazolin-4-one (Imi-3 and Imi-4)

Prepared according to general Procedure C on 1.9 mmol scale employing 1 mL of 1-phenylpropan-2-one (4 equiv., 7.5 mmol). The reaction crude was purified by flash column chromatography on silica gel (hexane:EtOAc 6:4 → 0:1) to afford the desired products as white solids in 97% yield. The two diastereoisomer were separated by flash column chromatography.

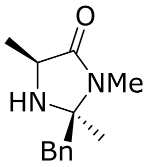

Imi-3: (2*S*,5*S*)-2-benzyl-2,3,5-trimethylimidazolin-4-one was obtained in 31% yield as a low melting solid. **^{1}H NMR** (400 MHz, $CDCl_3$) δ 7.36 – 7.22 (m, 2H), 7.15 – 7.08 (m, 2H), 3.47 (q, *J* = 6.8 Hz, 1H), 3.06 (d, *J* = 14.3 Hz, 1H), 2.92 (s, 3H), 1.67 (bs, 1H), 1.38 (s, 3H), 0.95 (d, *J* = 6.8 Hz, 3H). **^{13}C NMR** (75 MHz, $CDCl_3$) δ 175.5, 134.9, 130.2, 128.6, 127.3, 77.8, 53.8, 43.8, 26.0, 25.3, 17.3. **HRMS (ESI+ TOF) m/z:** $[M + Na]^+$ Calcd for $C_{13}H_{18}NO_2Na$ 241.1317; found 241.1311. **R_f** = 0.15 (Hexane-AcOEt 1:1).

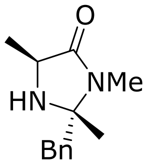

Imi-4: (2*R*,5*S*)-2-benzyl-2,3,5-trimethylimidazolin-4-one was obtained in 66% yield. **^{1}H NMR** (400 MHz, $CDCl_3$) δ 7.33 – 7.22 (m, 2H), 7.24 – 7.13 (m, 3H), 2.90 (d, *J* = 13.6 Hz, 1H), 2.89 (s, 3H), 2.80 (q, *J* = 6.9 Hz, 1H), 2.71 (d, *J* = 13.6 Hz, 1H), 1.59 (bs, 1H), 1.41 (s, 3H), 1.18 (d, *J* = 6.8 Hz, 3H). **^{13}C NMR** (75 MHz, $CDCl_3$) δ 175.5, 136.0, 130.3, 128.4, 127.0, 77.9, 54.0, 43.7, 27.0, 25.7, 18.2. **HRMS (ESI+ TOF) m/z:** $[M + Na]^+$ Calcd for $C_{13}H_{18}NO_2Na$ 241.1317; found 241.1312. **R_f** = 0.20 (Hexane-AcOEt 1:1). **M.p.** 94 °C-96 °C.

(*S*)-2,2,3,5-tetramethylimidazolidin-4-one (Imi-5)

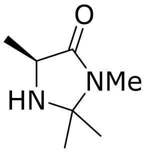

Prepared according to general Procedure C on 4.8 mmol scale. The desired product was obtained as a white solid in 65% yield (444 mg) after purification by flash column chromatography on silica gel (EtOAc → EtOAc:MeOH 9:1). All the analytical data are in agreement with the literature.[9]

^{1}H NMR (300 MHz, $CDCl_3$) δ 3.54 (q, *J* = 6.9 Hz, 1H), 2.78 (s, 3H), 1.81 (s, 1H), 1.41 (s, 3H), 1.35 (d, *J* = 6.9 Hz, 3H), 1.30 (s, 3H). **M.p.** 70°C-73°C.

(*S*)-5-(4-hydroxybenzyl)-2,2,3-trimethylimidazolidin-4-one (Imi-6)

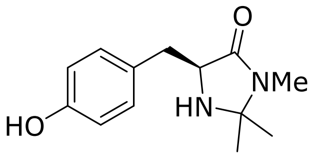

Prepared according to general Procedure C on 1 mmol scale. The desired product was obtained as a white solid 83% yield (195 mg) after purification by flash column chromatography on silica gel (EtOAc). All the analytical data are in agreement with the literature.[8]

^{1}H NMR (300 MHz, $CDCl_3$) δ 7.05 (d, *J* = 8.4 Hz, 2H), 6.72 (d, *J* = 8.4 Hz, 2H), 3.77 (t, *J* = 5.5 Hz, 1H), 3.11 – 2.92 (m, 2H), 2.76 (s, 3H), 1.28 (s, 3H), 1.19 (s, 3H). **M.p.** 83 °C-85 °C.

(2*S*,5*S*)-2-(*tert*-butyl)-2,3,5-trimethylimidazolidin-4-one (Imi-2)

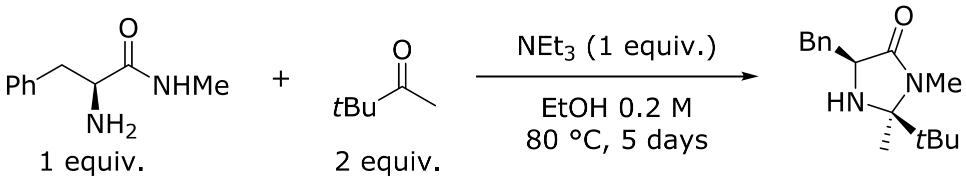

Into a 25 mL one necked flask, 306 mg of (*S*)-2-amino-*N*-methyl-3-phenylpropanamide (1.71 mmol, 1 equiv.) was dissolved into 9 mL of EtOH (0.2 M), then 430 μL of 3-methylbutan-2-one (3.43 mmol, 2 equiv.) and 240 μL of NEt_3 (1.71 mmol, 1 equiv.) were introduced. The flask was equipped with magnetic stirrer, molecular, sieves, reflux condenser and Dean-Stark apparatus. The reaction mixture was heated up at 80 °C (reflux) with an oil bath under nitrogen atmosphere and stirred for five days. After this time, the solvent was removed under reduced pressure. Then, the reaction crude was diluted with EtOAc and 5 mL of H_2O. The phases were separated and the aqueous one was extracted with 2x10 mL of EtOAc. The combined organic phases were dried over anhydrous Na_2SO_4, and the solvent was removed under reduced pressure. The reaction crude was purified by flash column chromatography (hexane:EtOAc 8:2 → 1:1) to afford the desired product in 15 % yield (67 mg) as a low melting solid, as a single diastereoisomer. All the analytical data are in agreement with the literature. [10]

1H NMR (300 MHz, $CDCl_3$) δ 7.35 – 7.19 (m, 5H), 3.76 (t, *J* = 6.0 Hz, 1H), 3.16 (dd, *J* = 13.6, 4.4 Hz, 1H), 3.06 (dd, *J* = 13.6, 6.8 Hz, 1H), 2.89 (s, 3H), 1.27 (s, 3H), 0.84 (s, 9H).

(2*S*,5*S*)-2-(*tert*-butyl)-2,3,5-trimethylimidazolidin-4-one (Imi-7)

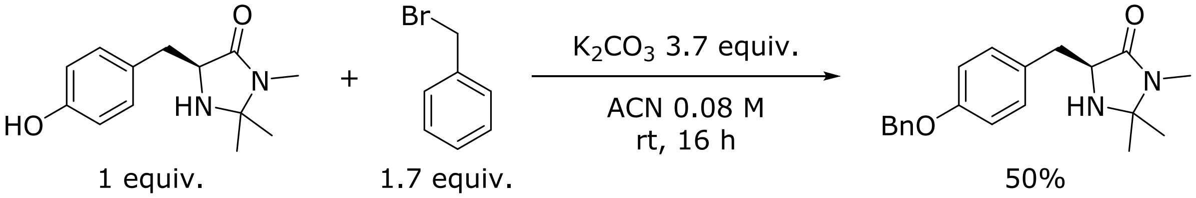

In 50 mL one-necked flask, (*S*)-5-(4-hydroxybenzyl)-2,2,3-trimethylimidazolidin-4-one (430 mg, 1.8 mmol, 1 equiv.) was dissolved in 22 mL of acetonitrile (0.08 M). After 15 minutes, 370 μL of benzyl bromide were added portion-wise, then the reaction mixture was stirred at room temperature overnight. After this time, the volatiles were removed under reduced pressure and the reaction crude was purified by flash column chromatography on silica gel (EtOAc:hexane 1:1 → EtOAc) to afford the desired product as a yellow oil in 50% yield.

^{1}H NMR (300 MHz, $CDCl_3$) δ 7.44 – 7.21 (m, 5H), 7.12 (d, *J* = 8.5 Hz, 2H), 6.89 (d, *J* = 8.6 Hz, 2H), 5.00 (s, 2H), 3.73 (t, *J* = 5.5 Hz, 1H), 3.11 – 2.86 (m, 2H), 2.71 (s, 3H), 1.23 (s, 3H), 1.13 (s, 3H). **^{13}C NMR** (75 MHz, $CDCl_3$) δ 173.4, 157.6, 136.9, 130.5, 130.4, 129.1, 128.5, 127.9, 127.4, 127.3, 114.9, 75.5, 69.8, 59.3, 36.0, 25.2, 25,0. **HRMS (ESI+ TOF) m/z:** $[M + Na]^+$ Calcd for $C_{20}H_{24}N_2O_2Na$ 347.1735; found 347.1737.

1,2,3,5-tetrakis(carbazol-9-yl)-4,6-dicyanobenzene (4CzIPN)

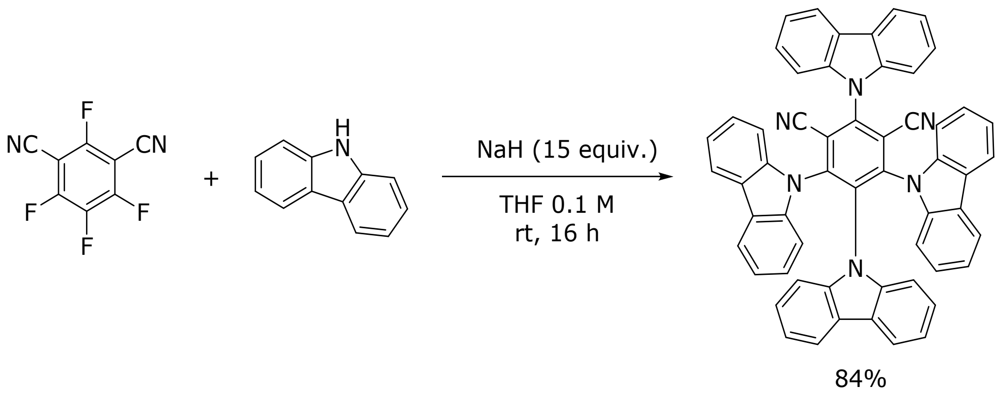

In a 50 mL two-neck flask under a nitrogen atmosphere 600 mg of NaH (60% mineral oil, 15 mmol, 15 equiv.) was washed with 10 ml of pentane (3 times). Then, 10 ml of dry THF were added and the carbazole (840 mg, 5 mmol, 5 equiv.) were introduced. The suspension was stirred for 1 hour at room temperature. After that time, the tetrafluoroIsophthalonitrile (200 mg, 1 mmol, 1 equiv.) was introduced portion-wise and the reaction mixture was stirred overnight at room temperature.
Then, 5 ml of H_2O were added and the THF was removed at reduced pressure. The reaction crude was extracted with 10 ml of $CHCl_3$ (2 times), the combined organic phases were dried out with Na_2SO_4 and the solvent was removed under reduced pressure. The desired product was crystallized with an antisolvent precipitation: the minimum quantity of $CHCl_3$ to solubilize 4CzIPN was employed, then hexane was added. The desired product was obtained as a yellow solid in 84% yield after filtration on Buckner and dried under high vacuum overnight. All the analytical data are in agreement with the literature. [11]
^{1}H NMR (300 MHz, $CDCl_3$) δ 8.24 (d, *J* = 7.7 Hz, 2H), 7.74 (m, 8H), 7.51 (m, 2H), 7.35 (d, *J* = 8.1, 2H), 7.24 (dd, *J* = 6.3, 2.5 Hz, 4H), 7.10 (m, 8H), 6.84 (t, *J* = 7.3 Hz, 4H), 6.64 (t, *J* = 7.7 Hz, 2 H).

4.5 Synthesis of Aldehydes starting materials

4-(benzyloxy)butan-1-ol

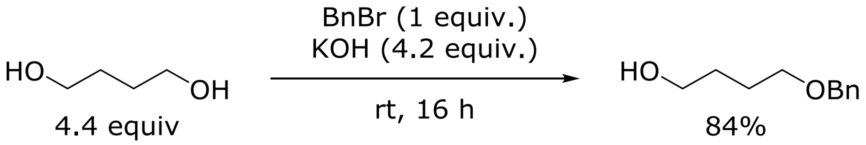

Into a two-necked 10 mL flask under nitrogen atmosphere, 700 mg of KOH (4.2 equiv., 24.8 mmol) were dissolved into 2.2 mL of 1,4-butandiole (4.4 equiv., 26 mmol). Then, 800 μL of benzyl bromide was added in four portions over 1 hour. The reaction mixture was stirred at room temperature overnight. After this time, the reaction was quenched with 4 mL of H_2O and extracted with 4 mL (2 times) of Et_2O. The combined organic phases were dried with Na_2SO_4 and the solvent was removed under reduced pressure. The reaction crude was purified with a flash column chromatography on silica gel (CH_2Cl_2-AcOEt 8:2) to afford the desired product as a transparent oil in 84 % yield (890 mg). All analytical data are in agreement with the literature. [12]
^{1}H NMR (300 MHz, $CDCl_3$) δ 7.41 – 7.29 (m, 5H), 4.52 (s, 2H), 3.63 (t, *J* = 5.7 Hz, 2H), 3.52 (t, *J* = 5.7 Hz, 2H), 2.40 (bs, 1H), 1.80 – 1.59 (m, 4H). **R_f** = 0.3 (CH_2Cl_2-EtOAc 1:1).

General procedure D: Synthesis of aldehydes of PIDA oxidation.

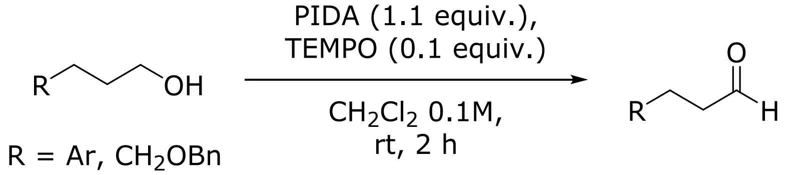

Into a one-necked flask, 6.1 mmol of alcohol were dissolved in 6 mL of dry CH_2Cl_2 (0.1M). Then, TEMPO (96 mg, 0.6 mmol, 0.1 equiv.) and PIDA (iodobenzene diacetate, 2.1 g, 6.6 mmol, 1.1equiv.) were added. The reaction mixture was stirred for 2 hours at room temperature. After that time, the reaction was diluted 25 mL of CH_2Cl_2 and quenched with 25 mL of a s.s. $Na_2S_2O_3$. The aqueous phase was extracted with CH_2Cl_2 (3 x 15 mL). The combined organic layers were washed with s.s. $NaHCO_3$ (40 mL), brine (40 mL), dried over Na_2SO_4 and concentrated in vacuo. The resulting residue was purified by flash column chromatography on silica gel.

3-(3,4-dimethoxyphenyl)propanal (1c)

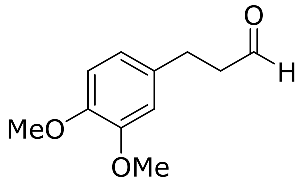

Prepared according to the general procedure D. The desired product was obtained as a transparent liquid in 60% yield after flash column chromatography (Hexane:EtOAc 9:1 → 85:15). All the analytical data are in agreement with the literature. [13]

^{1}H NMR (300 MHz, $CDCl_3$) δ 9.82 (t, *J* = 1.3 Hz, 1H), 6.84 – 6.69 (m, 3H), 3.87 (s, 3H), 3.85 (s, 3H), 2.91 (t, *J* = 7.8 Hz, 2H), 2.77 (t, *J* = 7.5 Hz, 2H).

4-(benzyloxy)butanal (1f)

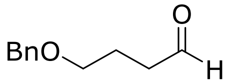

Prepared according to the general procedure D. The desired product was obtained as a transparent liquid in 64% yield (602 mg) after flash column chromatography (Hexane:EtOAc 98:2 → 8:2). All the analytical data are in agreement with the literature. [14]

^{1}H NMR (300 MHz, $CDCl_3$) δ 9.78 (s, 1H), 7.41 – 7.22 (m, 5H), 4.49 (s, 2H), 3.51 (t, *J* = 6.1 Hz, 2H), 2.55 (td, *J* = 7.1, 1.6 Hz, 2H), 1.95 (p, *J* = 6.8 Hz, 2H). **R_f** = 0.2 (Hexane-EtOAc 9:1).

10-undecenal (1e)

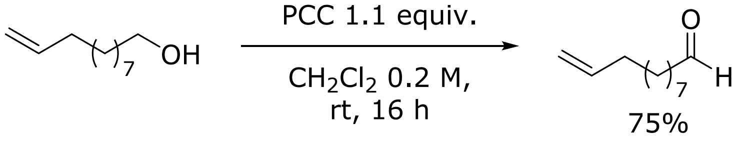

Into a Schlenk 250 ml flask under nitrogen atmosphere, 4 mL of 10-undecanolo (20 mmol, 1 equiv.) was dissolved into 100 mL of dry CH_2Cl_2 (0.2 M). Then, 4.7 g of PCC (22 mmol, 1.1 equiv.) were added and the reaction mixture was stirred at room temperature for 16 hours. After this time, the reaction mixture was filtered on a celite bed. The filtrate was evaporated under reduced pressure and purified by flash column chromatography on silica gel (Hex: AcOEt 98:2 -> 95:5) leading to the desired product as a transparent liquid in 75% yield (2.5 g). All analytical data are in agreement with the literature.[15]

^{1}H NMR (300 MHz, $CDCl_3$) δ 9.76 (s, 1H), 5.85-5.76 (m, 1H), 5.02-4.90 (m, 2 H), 2.41 (td, *J* = 7.2Hz, *J* = 1.7 Hz, 2H), 2.04 (m, 2H), 1.61 (m, 2H), 1.39-1.30 (m, 8H) ppm. **R_f** = 0.68 (Hexane-EtOAc 9:1).

2-naphthoyl chloride

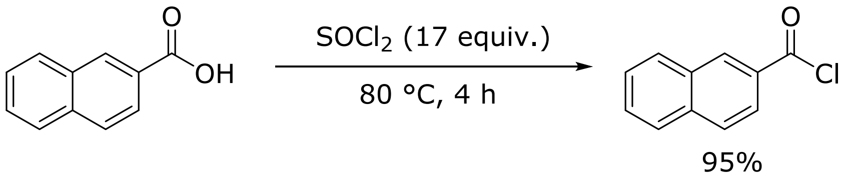

Into a one necked flask equipped with a condenser, 1 g of 2-naphthoic acid (5.8 mmol, 1 equiv.) was dissolved into 7.5 mL of $SOCl_2$ (103 mmol, 17 equiv.). The reaction mixture was stirred at 80 °C with an oil bath for 4 hours. After this time, the excess of $SOCl_2$ was removed under reduced pressure. The purity of the compound was confirmed by GC-MS.

4.6 Synthesis of the racemic 2-(2-Oxo-pyrrolidin-1-yl)-butyraldehyde **3aa**

1-(1-hydroxybutan-2-yl)pyrrolidin-2-one (4aa)

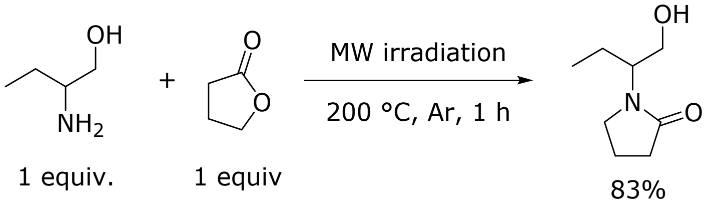

A solution of γ-butyrolactone (1.47 g, 17 mmol, 1 equiv.) and 2-amino-1-butanol (1.45 g, 17 mmol, 1 equiv.) was degassed with argon for 15 minutes. Then, the solution was transferred into a sealed vial under an inert atmosphere and subsequently heated up to 200 °C in a microwave reactor equipped with precise temperature control (Discover, CEM Corporation) for 1 hour. Reaction progress was monitored via TLC. The resulting crude mixture was purified by column chromatography on silica gel (CH_2Cl_2:MeOH = 96:4), yielding the target compound as a yellowish liquid in 83% yield (2.22 g). All the analytical data are in agreement with the literature.[16]

^{1}H NMR (300 MHz, $CDCl_3$) δ 5.84 (bs, 1H), 3.98 – 3.83 (m, 1H), 3.72 (dt, *J* = 11.5, 3.3 Hz, 1H), 3.60 (dd, *J* = 11.6, 8.3 Hz, 1H), 3.49 – 3.37 (m, 1H), 3.37 – 3.23 (m, 1H), 2.44 (td, *J* = 8.3, 2.5 Hz, 2H), 2.22 – 2.10 (m, 2H), 1.68 – 1.43 (m, 2H), 0.90 (td, *J* = 7.2, 2.5 Hz, 3H). **^{13}C NMR** (75 MHz, $CDCl_3$) δ = 128.7, 63.1, 56.3, 44.1, 42.3, 31.7, 21.2, 18.4, 10.8. **MS (APCI+) m/z:** $[M + H]^+$ Calcd for $C_8H_{16}NO_2$ 158.12; found 158.4.

2-(2-Oxo-pyrrolidin-1-yl)-butyraldehyde (3aa)

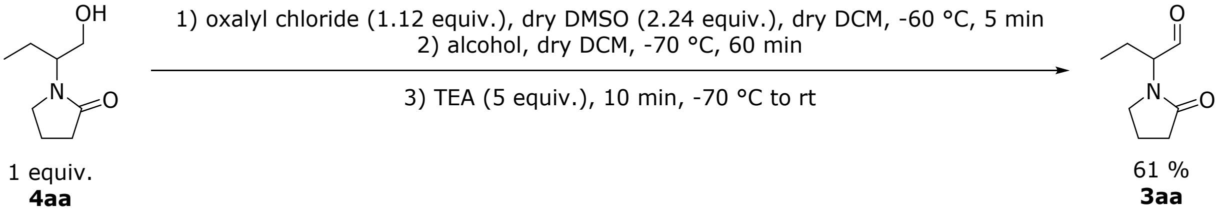

Into a 100 mL three-necked flask, 1.3 mL of oxalyl chloride (15 mmol, 1.12 equiv.) were dissolved in 31 mL of dry CH_2Cl_2 (0.48 M) and the solution was cooled to –60 °C with an acetone + dry ice bath. Then, a solution containing 2.15 mL of dry DMSO (30 mmol, 2.24 equiv.) and 7 mL of dry CH_2Cl_2 was added dropwise, and the mixture was stirred for 5 minutes at –60 °C. Subsequently, a solution of 2.125 g of **4aa** (13.5 mmol, 1 equiv.) in 9.7 mL of dry CH_2Cl_2 was introduced dropwise at –70 °C (temperature kept with an acetone + dry ice bath), and the reaction mixture was stirred for 60 minutes. Finally, 9.13 mL of TEA (68 mmol, 5 equiv.) were added, and the reaction mixture was stirred for additional 10 minutes. After this time, the reaction mixture was allowed to warm up to room temperature and quenched with 43 mL of water. It was then extracted with 70 mL of CH_2Cl_2, washed with 40 mL of HCl 1% aq and 40 mL of Na_2CO_3 5% aq. The organic layer was dried over anhydrous Na_2SO_4 and concentrated under reduced pressure. The desired product was obtained as a brown liquid in 61% yield (1.285 g).

^{1}H NMR (300 MHz, $CDCl_3$) δ 9.56 (s, 1H), 4.54 (dd, *J* = 10.8, 4.9 Hz, 1H), 3.35 (dtd, *J* = 23.6, 9.5, 6.4 Hz, 2H), 2.53 – 2.37 (m, 2H), 2.20 – 1.94 (m, 3H), 1.60 (dtd, *J* = 14.3, 7.2, 3.3 Hz, 1H), 0.98 (t, *J* = 7.4 Hz, 3H). **^{13}C NMR** (75 MHz, $CDCl_3$) δ 198.7, 175.9, 61.8, 44.0, 30.3, 18.8, 18.1, 10.3. **HRMS (ESI + TOF) m/z:** $[M + H]^+$ Calcd for $C_8H_{15}NO_2$ 156.1025; found 156.1022.

5 Screening of the reaction conditions

General Procedure E: asymmetric α addition of Lactams to aldehydes

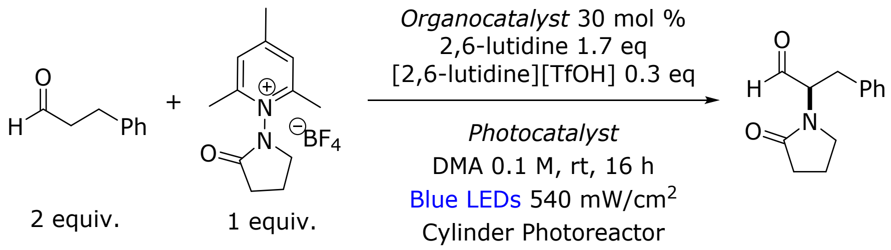

The organocatalyst (0.3 equiv.) the *N*-radical precursor **2** (88 mg, 1 equiv.), [2,6- lutidine][TfOH] (23mg, 0.075 mmol, 0.3 equiv.) were added in a 10 ml microwave vial closed with an aluminium/PTFE septum and three cycles vacuum-nitrogen were performed. 3 mL of DMA, hydrocinnamaldehyde **1d** (80 µl, 2 equiv.) and 60 µL of 2,6-lutidine (1.7 equiv., 0.5 mmol) were introduced through syringes. Three freeze-pump-thaw cycles have been performed to remove all the gases dissolved in the liquids. The reaction mixture was stirred at room temperature, for 15 h under Blue light irradiation (540 mW/cm^2) with the cylinder photoreactor (LEDs specification in Chapter 2.2). After this time, the reaction mixture was concentrated ad reduced pressure and purified by flash chromatography on silica gel (Hexane-AcOEt 2:8). The desired product was obtained as a yellow oil.

^{1}H NMR (300 MHz, $CDCl_3$) δ 9.66 (s, 1H), 4.73 (dd, *J* = 10.5 Hz, *J* = 5.6, 1H), 3.35 (dd, *J* = 14.7 Hz, *J* = 5.7 Hz, 1H), 3.30 – 3.22 (m, 1H), 3.15 (ddd, *J* = 9.1 Hz, *J* = 8.0 Hz, *J* = 5.8 Hz, 1H), 2.98 (dd, *J* = 14.6 Hz, *J* = 10.5 Hz, 1H), 2.49 – 2.21 (m, 2H), 2.10 – 1.82 (m, 2H). **^{13}C NMR** (75 MHz, $CDCl_3$) δ = 198.5, 176.0, 135.4, 132.0, 125.0, 118.7, 62.1, 45.7, 32.3, 30.6, 18.6. **HRMS (ESI +TOF) m/z:** $[M + Na]^+$ Calcd for $C_{13}H_{15}NO_2Na$ 240.1000; found 240.1002. **R_f** = 0.2 (Hexane-EtOAc 2:8), stained with $KMnO_4$.

-First, we test a few different organocatalysts and photocatalysts in the asymmetric addition of *N*-lactam radicals to hydrocinnamaldehyde.

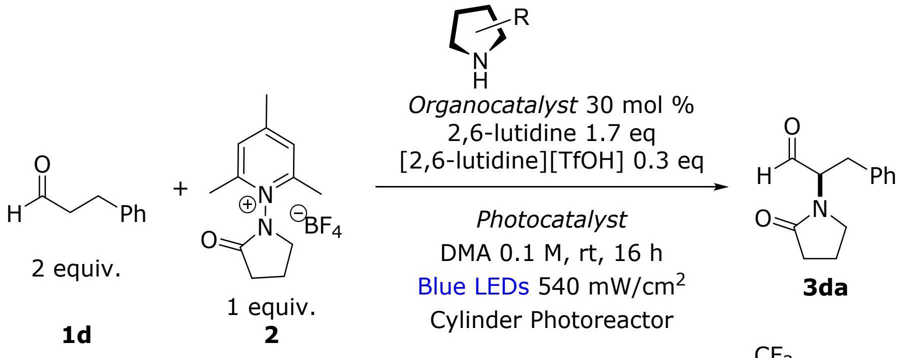

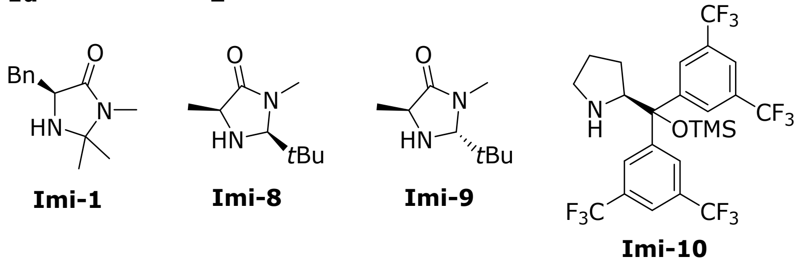

Entry	Organocatalyst	Photocatalyst (mol %)	3da Yield (%)

**1**	**Imi-1**	4CzIPN (2)	60
**2**	**Imi-1**	Ir(ppy)$_3$ (1)	50
**3**	**Imi-8**	4CzIPN (2)	12
**4**	**Imi-9**	4CzIPN (2)	20
**5**	**Imi-10**	4CzIPN (2)	-

Table S4 *First test of the asymmetric addition of lactams on aldehydes*

<u>General Procedure F</u>: asymmetric α addition of Lactams to aldehydes followed by an *in-situ* reduction.

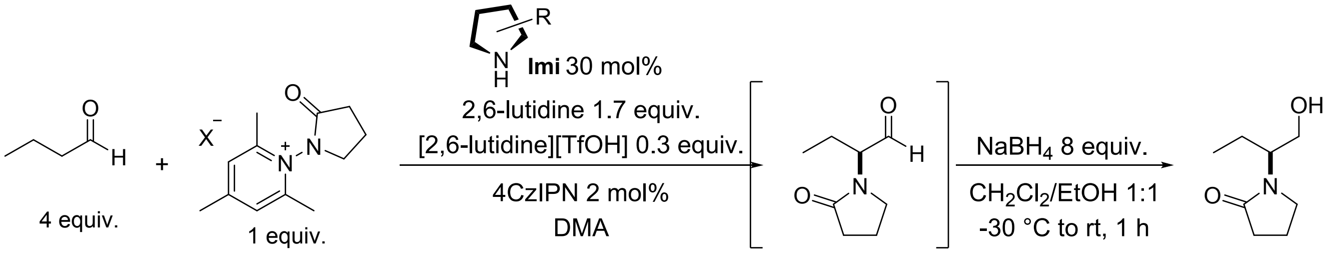

1a **2** **3aa** **4aa**

Into a 7 ml vial, the nitrogen radical precursor **2** (1 equiv., 0.3 mmol), 4.8 mg of 4-CzIPN (2 mol%), the organocatalyst (30 mol%) and 24 mg of [2,6-lutidine][TfOH] (0.3 equiv., 0.09 mmol) were introduced. The vial was sealed with a septum cap and three nitrogen-vacuum cycles were done. DMA, 60 μL of 2,6-lutidine (1.7 equiv., 0.5 mmol) were introduced through syringes. Three freeze-pump-thaw cycles have been performed to remove all the gases dissolved in the liquids. Then, the degassed daily freshly distilled butyric aldehyde (4 equiv., 1.2 mmol) was added, and the reaction mixture was irradiated for 16 hours with Blue LEDs. After this time, the reaction mixture was poured into a suspension of 8 equiv. of $NaBH_4$ in 2 mL of EtOH-CH_2Cl_2 (1: 1) at -30 °C (temperature kept with an acetone/dry ice bath). The reaction was stirred for 15 minutes at -30 °C, then the bath was allowed to warm up to room temperature in 30 minutes.
After 30 minutes, the reaction was quenched with a slow addition of a 0.5 M solution of citric acid in MeOH (1 mL). After that all the volatiles were removed under reduced pressure and crude was purified by flash column chromatography on silica gel CH_2Cl_2-MeOH 98-2 -> 95-5.

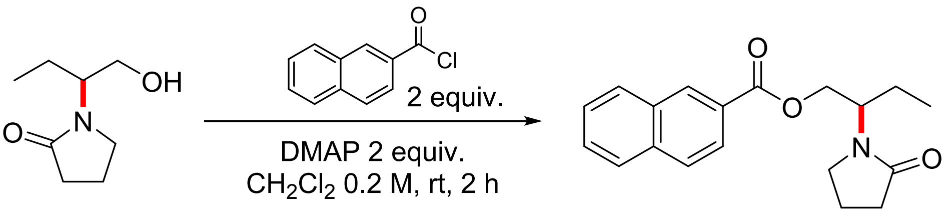

The desired alcohol **4aa** (1 equiv., previously obtained), DMAP (2 equiv.) and 2-naphthoyl chloride (2 equiv.) were introduced in a 3 ml vial and dissolved in dry CH_2Cl_2 (0.2 M). The vial was filled with nitrogen and the reaction was stirred for 2 h at room temperature. After this time, 1 ml of supersaturated aqueous $NaHCO_3$ was introduced into the vial and the phases were separated. The organic phase was dried with $NaHCO_3$ and the solvent was removed under reduced pressure. The reaction crude was purified by chromatographic column on silica gel.

-Counterion screening

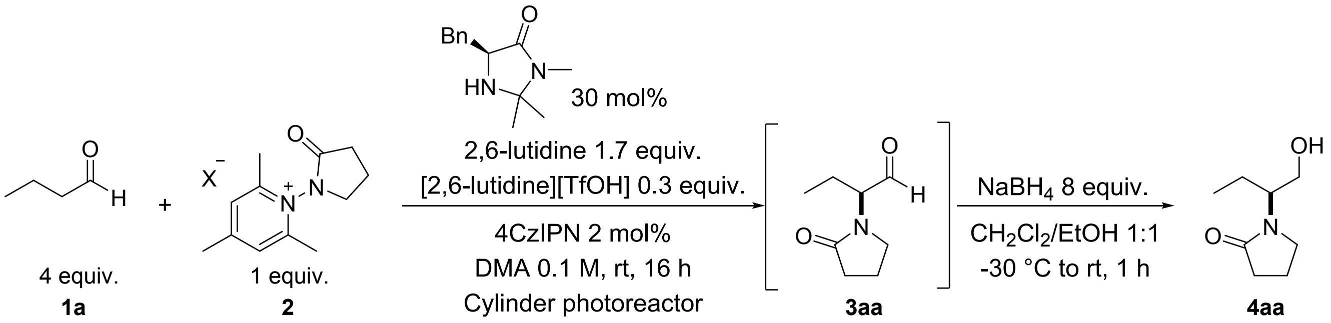

Entry	Counterion	4aa Yield (%)	4aa *e.e.* (%)*
**1**	BF_4^-	47	83
**2**	TfO^-	57	89

Table S5 *Counterion screening, *calculated on the corresponding 2-naphthoyl ester.*

-light source screening

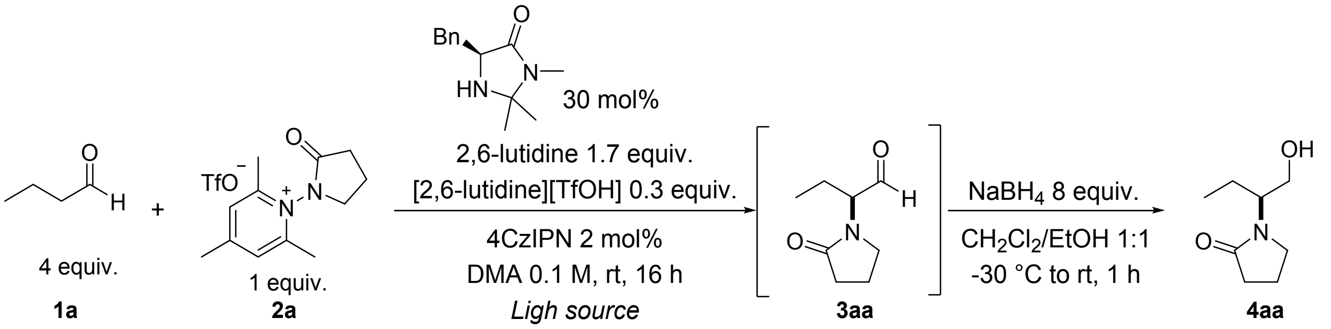

Entry	Photoreactor type	λ (nm)	Power	4aa Yield (%)
**1**	Cylinder photoreactor	460	540 mW/cm^2	57
**2**	Kessil Lamp	456	39 W	40
**3**	Plate Photoreactor	451	573 mW	55

Table S6 *Light source screening, the Photoreactors are described in Chapter 2.*

-Organocatalyst screening

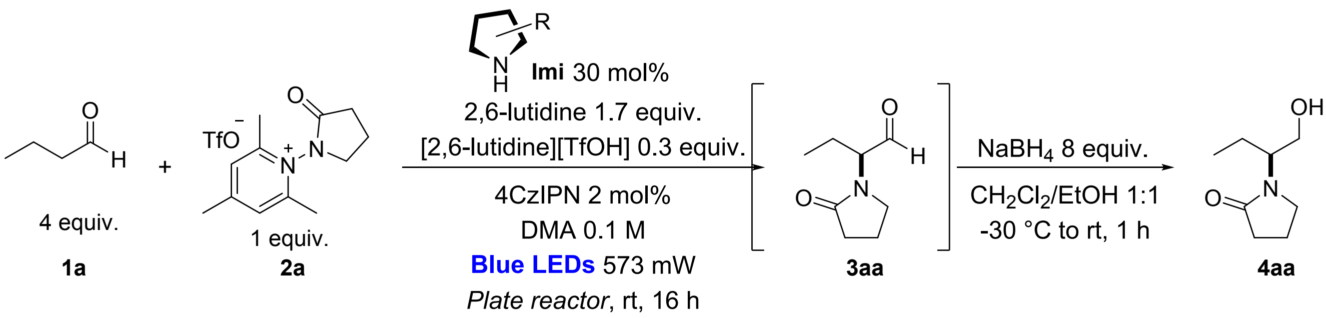

Entry	Organocatalyst	4aa Yield (%)	4aa *e.e.* (%)
**1**	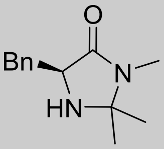 **Imi-1**	55	89
**2**	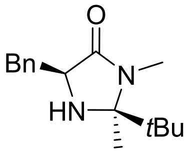 **Imi-2**	0	-
**3**	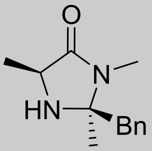 **Imi-3**	40	74
**4**	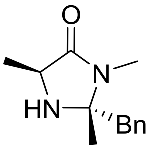 **Imi-4**	21	46
**5**	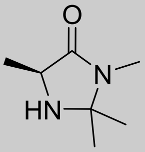 **Imi-5**	54	62
**6**	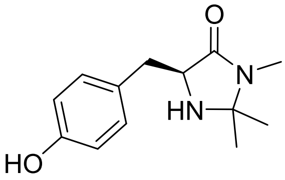 **Imi-6**	50	89
**7**	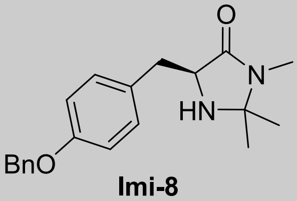 **Imi-8**	45	88

Table S7 *Organocatalyst screening*

-Concentration screening

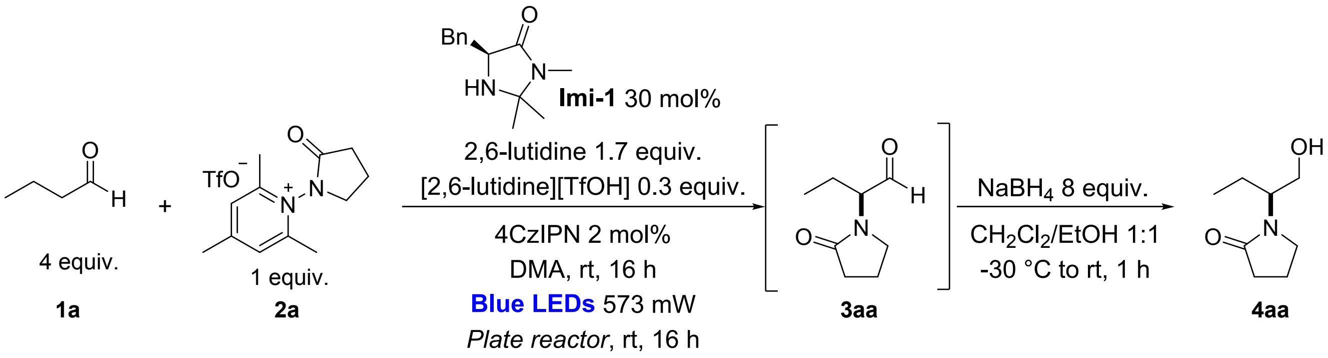

Entry	DMA (M)	4aa Yield (%)
**1**	0.05	24
**2**	0.1	55
**3**	0.2	70

Table S8 *Concentration screening.*

6 Substrate scope

General Procedure G: Photocatalytic asymmetric addition of lactam radicals to aldehydes followed by an *in-situ* reduction.

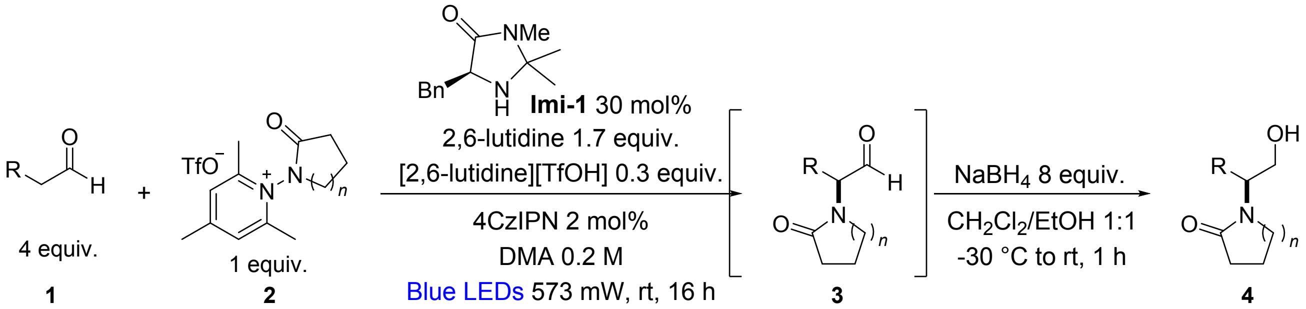

Into a 7 ml vial, the nitrogen the nitrogen radical precursor **2** (1 equiv., 0.3 mmol), 4.8 mg of 4-CzIPN (2 mol%), the first-generation MacMillan catalyst **Imi-1** (30 mol%) and 24 mg of [2,6-lutidine][TfOH] (0.3 equiv., 0.09 mmol) were introduced. The vial was sealed with a septum cap and three nitrogen-vacuum cycles were done. 1.5 ml of DMA (0.2 M), 60 µL of 2,6-lutidine (1.7 equiv., 0.5 mmol) and the aldehyde **1** (4 equiv., 1.2 mmol) were introduced through syringes. Three freeze-pump-thaw cycles have been performed to remove all the gases dissolved in the liquids. The reaction mixture was irradiated for 16 hours with Blue LEDs (450 nm, 573 mW) using the plate photoreactor. After this time, the reaction mixture was poured into a suspension of 8 equiv. of $NaBH_4$ in 2 mL of EtOH-CH_2Cl_2 (1: 1) at -30 °C (temperature kept with an acetone/dry ice bath). The reaction was stirred for 15 minutes at -30 °C, then the bath was allowed to warm up to room temperature in 30 minutes. After 30 minutes, the reaction was quenched with a slow addition of a 0.5 M solution of citric acid in MeOH (1 mL). After that all the volatiles were removed under reduced pressure and crude was purified by flash column chromatography on silica gel.

(*S*)-1-(1-hydroxybutan-2-yl)pyrrolidin-2-one (4aa)

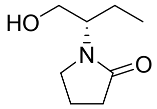

Prepared according to general procedure G. Butanal **1a** must be daily and freshly distilled before running the reaction otherwise a yield drop was observed. The crude mixture was purified by flash column chromatography on silica gel (CH_2Cl_2 -> CH_2Cl_2-MeOH 96:4) afford the desired product as yellow oil in 70 % yield (33 mg). All the analytical data are in agreement with the literature.[16]

^{1}H NMR (300 MHz, $CDCl_3$) δ 3.88 (dd, *J* = 11.9, 6.4 Hz, 1H), 3.74 (d, *J* = 11.4 Hz, 1H), 3.64 (t, *J* = 7.6 Hz, 1H), 3.44 (dd, *J* = 9.5, 7.1 Hz, 1H), 3.39 – 3.30 (m, 1H), 2.62 (bs, 1H), 2.45 (t, *J* = 8.1 Hz, 2H), 2.06 (q, *J* = 8.7 Hz, 2H), 1.55 (ddd, *J* = 22.6, 14.3, 6.9 Hz, 2H), 0.92 (t, *J* = 7.4 Hz, 3H). **^{13}C NMR** (75 MHz, $CDCl_3$) δ 176.2, 62.4, 55.3, 43.2, 31.3, 20.9, 18.0, 10.4. **MS (APCI+) m/z:** $[M + H]^+$ Calcd for $C_8H_{16}NO_2$ 158.12; found 158.4. **$[\alpha]_D^{27}$** = -10.72 (*c* 1.00, MeOH).

(*S*)-1-(1-hydroxy-3-methylbutan-2-yl)pyrrolidin-2-one (4ba)

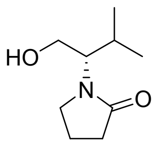

Prepared according to general procedure G. The crude mixture was purified by flash column chromatography on silica gel (CH_2Cl_2 -> CH_2Cl_2-MeOH 98:2) afford the desired product as yellow oil in 53 % yield (26.8 mg).

^{1}H NMR (300 MHz, $CDCl_3$) δ 3.82 (d, *J* = 9.0 Hz, 1H), 3.62 (d, *J* = 7.0 Hz, 2H), 3.47 (t, *J* = 8.3 Hz, 1H), 3.38-3.30 (m, 1H), 2.42 (d, *J* = 8.7 Hz, 2H), 2.10 – 1.98 (m, 2H), 1.94 – 1.85 (m, 1H), 0.96 (d, *J* = 6.8, 3H), 0.85 (d, *J* = 6.8, 3H). **^{13}C NMR** (75 MHz, $CDCl_3$) δ 177.3, 61.6, 60.9, 45.1, 31.6, 27.0, 20.1, 19.8, 18.5. **HRMS (ESI + TOF) m/z:** $[M + Na]^+$ Calcd for $C_9H_{17}NO_2Na$ 194.1157; found 194.1152. $[\alpha]_D^{25}$ = -10.30 (*c* 1.64, MeOH).

(*S*)-1-(1-(3,4-dimethoxyphenyl)-3-hydroxypropan-2-yl)pyrrolidin-2-one (4ca)

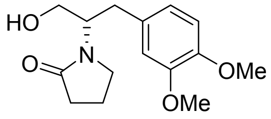

Prepared according to general procedure G. The crude mixture was purified by flash column chromatography on silica gel (CH_2Cl_2 -> CH_2Cl_2-MeOH 95:5) afford the desired product as yellow oil in 48% yield (40 mg).

^{1}H NMR (400 MHz, $CDCl_3$) δ 6.81 – 6.69 (m, 3H), 4.15 – 4.00 (m, 1H), 3.85 (d, *J* = 2.0 Hz, 6H), 3.82 – 3.62 (m, 2H), 3.33 – 3.12 (m, 2H), 2.87 (d, *J* = 7.9 Hz, 2H), 2.43 – 2.26 (m, 2H), 1.98 – 1.81 (m, 2H). **^{13}C NMR** (101 MHz, $CDCl_3$) δ 176.5, 149.0, 147.8, 130.5, 121.0, 112.1, 111.3, 63.3, 57.1, 56.06, 56.02, 46.5, 34.0, 31.7, 18.7. **HRMS (ESI+ TOF) m/z:** $[M + Na]^+$ Calcd for $C_{15}H_{21}NO_4Na$ 302.1368; found 302.1368. **R_f** = 0.35 (CH_2Cl_2-MeOH 95:5). $[\alpha]_D^{27}$ = -19.25 (*c* 0.42, MeOH).

(*S*)-1-(1-hydroxy-3-phenylpropan-2-yl)pyrrolidin-2-one (4da)

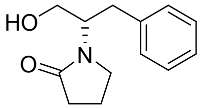

Prepared according to general procedure G. The crude mixture was purified by flash column chromatography on silica gel (AcOEt -> AcOEt-MeOH 9:1) afford the desired product as yellow oil in 58% yield (38 mg) with 90% *e.e.* All the analytical data are in agreement with the literature.[17]

^{1}H NMR (300 MHz, $CDCl_3$) δ 7.37 – 7.19 (m, 5H), 4.13 – 3.99 (m, 1H), 3.90 – 3.68 (m, 2H), 3.38 – 3.11 (m, 2H), 2.99 (dd, *J* = 7.8, 4.6 Hz, 2H), 2.37 (dt, *J* = 7.6, 3.5 Hz 2H), 2.06 – 1.82 (m, 2H). **^{13}C NMR** (75 MHz, $CDCl_3$) δ 176.5, 129.0, 128.7, 126.7, 63.5, 57.6, 46.9, 34.5, 31.7, 29.8, 18.7 **HRMS (ESI+ TOF) m/z:** $[M + Na]^+$ Calcd for $C_{13}H_{17}NO_2Na$ 242.1157; found 242.1151. **HPLC** (AD, isopropanol/*n*-hexane = 5/95, flow rate = 0.9 mL/min, l = 254 nm) tR = 24.1 min (minor), 27.0 min (major). $[\alpha]_D^{26}$ = -13.02 (*c* 1.08, MeOH).

(*S*)-1-(1-hydroxyundec-10-en-2-yl)pyrrolidin-2-one (4ea)

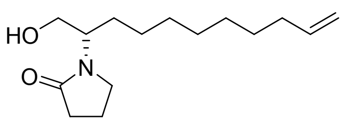

Prepared according to general procedure G. The crude mixture was purified by flash column chromatography on silica gel (CH_2Cl_2 -> CH_2Cl_2-MeOH 98:2) afford the desired product as yellow oil in 60% yield (45.5 mg).

^{1}H NMR (400 MHz, $CDCl_3$) δ 5.79 (ddt, *J* = 16.9, 10.2, 6.6 Hz, 1H), 5.03 – 4.87 (m, 2H), 4.03 – 3.95 (m, 1H), 3.68 (dd, *J* = 11.5, 4.0 Hz, 1H), 3.56 (dd, *J* = 11.6, 8.3 Hz, 1H), 3.42 – 3.36 (m, 1H), 3.33 – 3.27 (m, 1H), 2.41 (t, *J* = 8.1 Hz, 2H), 2.05 – 1.98 (m, 4H), 1.54 – 1.44 (m, 2H), 1.39 – 1.31 (m, 5H), 1.30 – 1.20 (m, 5H).**^{13}C NMR** (101 MHz, $CDCl_3$) δ 176.6, 139.2, 114.2, 63.4, 54.6, 44.1, 33.8, 31.7, 29.5, 29.4, 29.1, 28.9, 28.1, 26.2, 18.4. **HRMS (ESI+ TOF) m/z:** $[M + Na]^+$ Calcd for $C_{15}H_{27}NO_2Na$ 276.1939; found 276.1939. **R_f** = 0.62 (CH_2Cl_2). $[\alpha]_D^{27}$ = -3.99 (*c* 0.89, MeOH).

(S) 4-(benzyloxy)-1-hydroxybutan-2-yl)pyrrolidine-2-one (4fa)

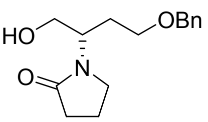

Prepared according to general procedure G. The crude mixture was purified by flash column chromatography on silica gel (CH_2Cl_2 -> CH_2Cl_2-MeOH 97:3) afford the desired product as yellow oil in 53% yield (42 mg) with 92% *e.e.*

^{1}H NMR (300 MHz, $CDCl_3$) δ 7.43 – 7.24 (m, 5H), 4.50 (s, 2H), 4.11-3.99 (m, 1H), 3.82 – 3.62 (m, 2H), 3.62 – 3.31 (m, 4H), 2.51 – 2.34 (m, 2H), 2.10 – 1.81 (m, 4H). **^{13}C NMR** (75 MHz, $CDCl_3$) δ 176.4, 138.1, 128.5, 127.9, 127.8, 73.4, 67.4, 63.7, 53.2, 45.8, 31.7, 28.4, 18.6. **HRMS (ESI+ TOF) m/z:** $[M + Na]^+$ Calcd for $C_{15}H_{21}NO_3Na$ 286.1419; found 286.1424. **HPLC** (AD, isopropanol/*n*-hexane 5/95, flow rate = 0.9 mL/min, l = 254 nm) tR = 39.7 min (minor), 45.3 min (major). **$[\alpha]_D^{28}$** = -8.52 (*c* 1.73, MeOH).

(*S*)-1-(1-hydroxybutan-2-yl)piperidin-2-one (4ab)

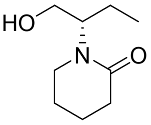

Prepared according to general procedure G. Butanal must be daily and freshly distilled before running the reaction otherwise a yield drop was observed. The crude mixture was purified by flash column chromatography on silica gel (CH_2Cl_2 -> CH_2Cl_2-MeOH 97:3) afford the desired product as yellow oil in 40 % yield (20.5 mg).

^{1}H NMR (300 MHz, $CDCl_3$) δ 4.32 – 4.14 (m, 1H), 3.69 (dd, *J* = 11.6, 3.9 Hz, 1H), 3.60 (dd, *J* = 11.6, 8.2 Hz, 1H), 3.29 – 3.05 (m, 2H), 2.45 – 2.37 (m, 2H), 1.87 – 1.70 (m, 4H), 1.63 – 1.48 (m, 2H), 0.89 (t, *J* = 7.4 Hz, 3H). **^{13}C NMR** (75 MHz, $CDCl_3$) δ 172.0, 63.3, 59.4, 44.2, 32.6, 23.3, 20.8, 20.7, 10.9. **HRMS (ESI+ TOF) m/z:** $[M + Na]^+$ Calcd for $C_9H_{17}NO_2Na$ 194.1157; found 194.1154. **$[\alpha]_D^{28}$** = -2.86 (*c* 1.35, MeOH).

(*S*)-1-(1-hydroxybutan-2-yl)azepan-2-one (4ac)

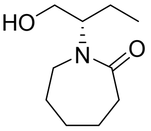

Prepared according to general procedure G. Butanal must be daily and freshly distilled before running the reaction otherwise a yield drop was observed. The crude mixture was purified by flash column chromatography on silica gel (CH_2Cl_2-MeOH 99:1 -> CH_2Cl_2-MeOH 95:5) afford the desired product as yellow oil in 11 % yield (6 mg).

^{1}H NMR (300 MHz, $CDCl_3$) δ 4.49 – 4.29 (m, 1H), 3.69 (dd, *J* = 11.5, 4.1 Hz, 1H), 3.52 (dd, *J* = 11.6, 8.8 Hz, 1H), 3.32 – 3.18 (m, 2H), 2.62 – 2.41 (m, 2H), 1.93 – 1.57 (m, 6H), 1.59 – 1.37 (m, 2H), 0.90 (t, *J* = 7.4 Hz, 3H). **^{13}C NMR** (75 MHz, $CDCl_3$) δ 178.4, 73.0, 63.7, 59.0, 44.6, 37.7, 30.0, 28.8, 23.5, 21.5, 11.3, 10.9. **HRMS (ESI+ TOF) m/z:** $[M + Na]^+$ Calcd for $C_{10}H_{19}NO_2Na$ 208.1313; found 208.1308.

<u>General Procedure H</u>: Synthesis of the 2-naphthoyl ester

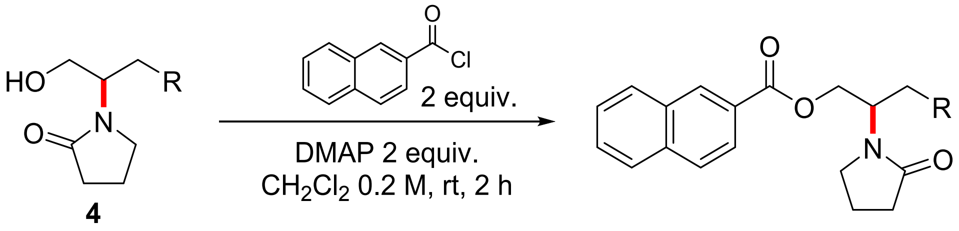

The desired alcohol **4** (1 equiv., obtained following the previous general procedure C), DMAP (2 equiv.) and 2-naphthoyl chloride (2 equiv.) were introduced in a 3 ml vial and dissolved in dry CH_2Cl_2 (0.2 M). The vial was filled with nitrogen and the reaction was stirred for 2 h at room temperature. After this time, 1 ml of supersaturated aqueous $NaHCO_3$ was introduced into the vial and the

solvents were removed under reduced pressure. The reaction crude was purified by chromatographic column on silica gel.

(S)-2-(2-oxopyrrolidin-1-yl)butyl 2-naphthoate

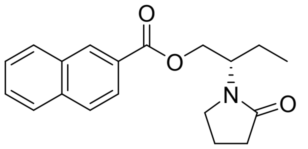

Prepared according to general procedure H on 0.16 mmol scale. The crude mixture was purified by flash column chromatography on silica gel (Hexane-AcOEt 7:3 -> Hexane-AcOEt 1:1) to afford the desired product as white solid in 52 % yield (26 mg) with 89% *e.e.*

^{1}H NMR (300 MHz, $CDCl_3$): δ 8.58 (s, 1H), 7.99 (dd, *J* = 15.4, 2H), 7.88 (d, *J* = 8.5 Hz, 2H), 7.65 – 7.49 (m, 2H), 4.59 – 4.46 (m, 2H), 4.35 (t, *J* = 7.8 Hz, 1H), 3.51 – 3.31 (m, 2H), 2.39 (hept, *J* = 8.3 Hz, *J* = 7.8 Hz, 2H), 2.01 (p, *J* = 7.5 Hz, 2H), 1.71 – 1.62 (m, 2H), 0.98 (t, *J* = 7.4 Hz, 3H). **^{13}C NMR** (75 MHz, $CDCl_3$) δ = 175.9, 166.6, 135.7, 131.4, 129.6, 128.5, 127.9, 126.8, 125.2, 64.6, 51.9, 43.0, 31.4, 21.8, 18.6, 10.7. **HRMS (ESI+ TOF) m/z:** $[M + Na]^+$ Calcd for $C_{19}H_{21}NO_3Na$ 334.1419; found 334.1416. **R_f** = 0.39 (Hexane-AcOEt 1:1). **HPLC** (Cellulose-1 5u Phenomenex, isopropanol/*n*-hexane 10/90, flow rate = 0.9 mL/min, l = 254 nm) tR = 31.9 min (minor), 43.0min (major). **$[\alpha]_D^{26}$** = -18.30 (*c* 0.86, MeOH). **M.p.** 67 °C-70 °C.

(S)-3-methyl-2-(2-oxopyrrolidin-1-yl)butyl 2-naphthoate

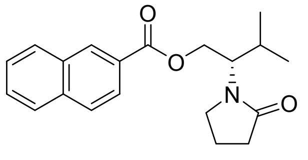

Prepared according to general procedure H on 0.18 mmol scale. The crude mixture was purified by flash column chromatography on silica gel (Hexane-AcOEt 7:3) to afford the desired product as white solid in 43% yield (24 mg) with 91% *e.e.*

^{1}H NMR (300 MHz, $CDCl_3$) δ 8.56 (s, 1H), 7.97 (dd, *J* = 12.2, 8.2 Hz, 2H), 7.87 (d, *J* = 8.5 Hz, 2H), 7.65 – 7.48 (m, 2H), 4.58 (dd, *J* = 11.7, 8.7 Hz, 1H), 4.45 (dd, *J* = 11.7, 3.6 Hz, 1H), 2.51 – 2.25 (m, 1H), 3.52 – 3.31 (m, 2H), 2.51 – 2.25 (m, 2H), 1.99 (dd, *J* = 9.8, 5.1 Hz, 3H), 1.09 (d, *J* = 6.6 Hz, 3H), 0.95 (d, *J* = 6.6 Hz, 3H). **^{13}C NMR** (75 MHz, $CDCl_3$) δ 175.8, 166.5, 135.7, 132.6, 131.3, 129.5, 128.5, 128.4, 127.8, 127.1, 126.8, 125.1, 63.5, 56.3, 43.6, 31.3, 27.5, 20.0, 19.8, 18.7. **HRMS (ESI+ TOF) m/z:** $[M + Na]^+$ Calcd for $C_{20}H_{23}NO_3Na$ 348.1576; found 348.1573. **R_f** = 0.15 (Hexane-AcOEt 7:3). **HPLC** (Cellulose-1 5u Phenomenex, isopropanol/*n*-hexane 10/90, flow rate = 0.9 mL/min, l = 254 nm) tR = 19.4 min (major), 23.3 min (minor). **$[\alpha]_D^{26}$** = -43.27 (*c* 1.04, MeOH). **M.p.** 113 °C-115 °C.

(*S*)-3-(3,4-dimethoxyphenyl)-2-(2-oxopyrrolidin-1-yl)propyl 1 naphthoate

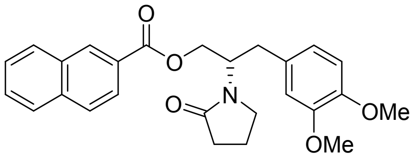

Prepared according to general procedure H on 0.04 mmol scale. The crude mixture was purified by flash column chromatography on silica gel (Hexane-AcOEt 7:3 -> Hexane-AcOEt 3:7) to afford the desired product as yellow oil in 39% yield (6.5 mg) with 91% *e.e.*

^{1}H NMR (300 MHz, $CDCl_3$) δ 8.65 – 8.51 (m, 1H), 8.08 – 7.96 (m, 1H), 7.89 (d, *J* = 8.6 Hz, 2H), 7.58 (dqd, *J* = 8.2, 6.9, 1.5 Hz, 2H), 6.79 (d, *J* = 3.6 Hz, 3H), 4.86 (qd, *J* = 7.9, 4.2 Hz, 1H), 4.60 (dd, *J* = 11.5, 8.1 Hz, 1H), 4.38 (dd, *J* = 11.5, 4.3 Hz, 1H), 3.86 (d, *J* = 4.4 Hz, 6H), 3.48 – 3.30 (m, 2H), 3.07 – 2.87 (m, 2H), 2.30 (t, *J* = 8.5 Hz, 2H), 1.99 – 1.83 (m, 2H). **^{13}C NMR** (75 MHz, $CDCl_3$) δ 175.6, 166.5, 149.1, 147.9, 135.7, 132.6, 131.4, 129.5, 129.4, 128.5, 128.4, 127.9, 127.0, 126.8, 125.2, 121.0, 111.8, 111.3, 64.2, 56.0, 55.9, 51.4, 44.0, 34.7, 31.3, 18.5. **HRMS (ESI+ TOF) m/z:** $[M + Na]^+$ Calcd for $C_{26}H_{27}NO_5Na$ 456.1787; found 456.1788. **R_f** = 0.16 (Hexane-AcOEt 1:1). **HPLC** (Cellulose-1 5u Phenomenex, ethanol/*n*-hexane 10/90, flow rate = 0.9 mL/min, l= 254 nm) tR = 38.7 min (major), isomer 2: 46.591 min (minor). **$[\alpha]_D^{26}$** = -36.09 (*c* 0.14, MeOH).

(*S*)-2-(2-oxopyrrolidin-1-yl)undec-10-en-1-yl 2-naphthoate

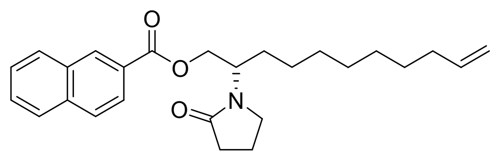

Prepared according to general procedure H on 0.23 mmol scale. The crude mixture was purified by flash column chromatography on silica gel (Hexane-AcOEt 7:3 -> Hexane-AcOEt 6:4) to afford the desired product as yellow oil in 39% yield (36 mg) with 90% *e.e.*

^{1}H NMR (300 MHz, $CDCl_3$) δ 8.57 (s, 1H), 8.01 (dd, *J* = 8.6, 1.7 Hz, 2H), 7.95 (d, *J* = 7.2 Hz, 1H), 7.87 (d, *J* = 8.7 Hz, 1H), 7.56 (p, *J* = 7.0 Hz, 2H), 5.80 (ddt, *J* = 16.9, 10.1, 6.7 Hz, 1H), 5.11 – 4.82 (m, 3H), 4.65 – 4.44 (m, 3H), 4.30 (dd, *J* = 10.8, 3.7 Hz, 1H), 3.49 – 3.29 (m, 2H), 2.38 (h, *J* = 8.9 Hz, 2H), 2.01 (p, *J* = 6.9 Hz, 5H), 1.60 (q, *J* = 6.5 Hz, 2H), 1.44 – 1.22 (m, 5H). **^{13}C NMR** (75 MHz, $CDCl_3$) δ 175.6, 166.4, 139.1, 135.6, 132.5, 131.2, 129.4, 128.36, 128.30, 127.7, 127.0, 126.6, 125.1, 114.1, 64.5, 50.8, 50.2, 42.8, 33.7, 31.3, 30.8, 28.9, 28.8, 28.4, 26.0, 18.4. **HRMS (ESI+ TOF) m/z:** $[M + Na]^+$ Calcd for $C_{26}H_{33}NO_3Na$ 430.2358; found 430.2360. **R_f** = 0.31 (Hexane-AcOEt 1:1). **HPLC** (Cellulose-1 5u Phenomenex, isopropanol/*n*-hexane 10/90, flow rate = 0.9 mL/min, l = 254 nm) tR = 20.4 min (minor), 26.0 min (major). **$[\alpha]_D^{26}$** = -23.66 (*c* 0.1, MeOH).

(*S*)-2-(2-oxopiperidin-1-yl)butyl 2-naphthoate

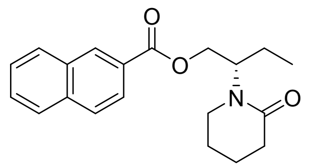

Prepared according to general procedure H on 0.06 mmol scale. The crude mixture was purified by flash column chromatography on silica gel (Hexane-AcOEt 7:3) to afford the desired product as deliquescent solid in 63% yield (10 mg) with 86% *e.e.*

^{1}H NMR (300 MHz, $CDCl_3$) δ 8.58 (d, *J* = 1.6 Hz, 1H), 8.02 (dd, *J* = 8.7, 1.7 Hz, 1H), 7.96 (d, *J* = 7.8 Hz, 1H), 7.88 (d, *J* = 8.4 Hz, 2H), 7.65 – 7.49 (m, 2H), 5.02 (p, *J* = 7.8 Hz, 1H), 4.57 (dd, *J* = 11.5, 8.7 Hz, 1H), 3.24 (hept, *J* = 6.1 Hz, 2H), 2.55 – 2.26 (m, 2H), 1.83 – 1.57 (m, 2H), 0.98 (t, *J* = 7.4 Hz, 3H). **^{13}C NMR** (75 MHz, $CDCl_3$) δ 170.8, 166.6, 135.7, 132.6, 131.3, 129.6, 128.46, 128.41, 127.9, 127.3, 126.8, 125.3, 64.3, 53.4, 42.5, 32.7, 23.4, 21.2, 21.0, 10.7. **HRMS (ESI+ TOF) m/z:** $[M + Na]^+$ Calcd for $C_{20}H_{23}NO_3Na$ 348.1576: found 348.1573. **R_f** = 0.15 (Hexane-AcOEt 1:1). **HPLC** (Cellulose-1 5u Phenomenex, isopropanol/*n*-hexane 10/90, 0.9 mL/min, l = 254 nm) tR = 28.8 min (major), 32.0 min (minor). **$[\alpha]_D^{26}$** = -37.40 (*c* 0.26, MeOH).

(*S*)-2-(2-oxoazepan-1-yl)butyl 2-naphthoate

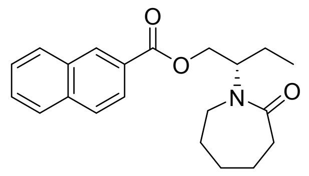

Prepared according to general procedure H on 0.03 mmol scale. The crude mixture was purified by flash column chromatography on silica gel (Hexane-AcOEt 7:3) to afford the desired product as yellow oil in 73% yield (6.3 mg) with 74% *e.e.*

^{1}H NMR (300 MHz, $CDCl_3$) δ 8.59 (s, 1H), 8.03 (dd, *J* = 8.6, 1.7 Hz, 1H), 7.96 (d, *J* = 7.9 Hz, 1H), 7.88 (d, *J* = 8.3 Hz, 2H), 7.72 – 7.48 (m, 2H), 5.08 – 4.91 (m, 1H), 4.48 – 4.27 (m, 2H), 3.38 – 3.29 (m, 2H), 2.64 – 2.54 (m, 2H), 1.75 – 1.52 (m, 8H), 0.98 (t, *J* = 7.4 Hz, 3H). **^{13}C NMR** (75 MHz, $CDCl_3$) δ 177.4, 165.4, 136.1, 133.0, 131.8, 130.0, 128.9, 128.8, 128.3, 127.7, 127.2, 125.7, 100.5, 65.5, 54.4, 44.1, 38.2, 30.5, 30.2, 29.7, 24.1 22.4, 11.3. **HRMS (ESI+ TOF) m/z:** $[M + Na]^+$ Calcd for $C_{21}H_{25}NO_3Na$ 362.1732; found 362.1736. **R_f** = 0.13 (Hexane-AcOEt 7:3). **HPLC** (Cellulose-1 5u Phenomenex, isopropanol/*n*-hexane 10/90, flow rate = 0.9 mL/min, l = 254 nm) tR = 17.3 min (minor), 19.1 min (major). **$[\alpha]_D^{26}$** = -18.58 (*c* 0.1, MeOH).

General Procedure I: Photocatalytic addition of lactam radicals to aldehydes followed by an *in-situ* reduction: Racemates synthesis.

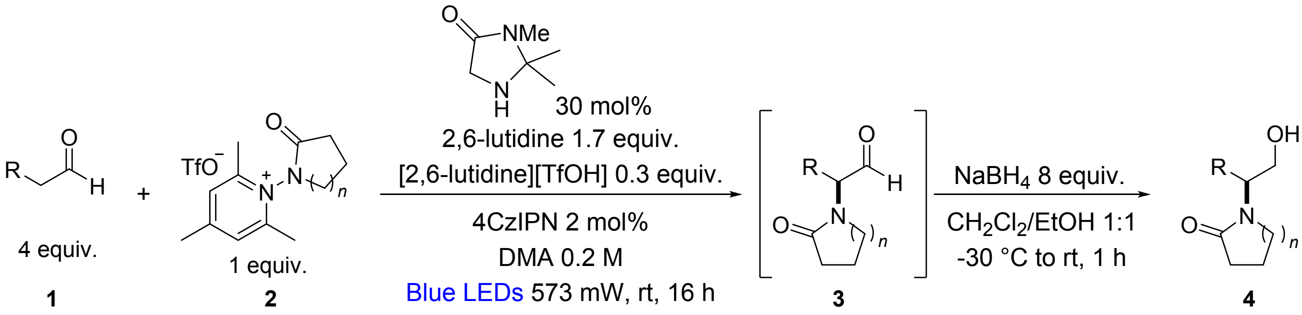

Into a 7 ml vial, the nitrogen the nitrogen radical precursor **2** (1 equiv., 0.3 mmol), 4.8 mg of 4-CzIPN (2 mol%), the organocatalyst (30 mol%) and 24 mg of [2,6-lutidine][TfOH] (0.3 equiv., 0.09 mmol) were introduced. The vial was sealed with a septum cap and three nitrogen-vacuum cycles were done. 1.5 ml of DMA (0.2 M), 60 µL of 2,6-lutidine (1.7 equiv., 0.5 mmol) and the aldehyde**1** (4 equiv., 1.2 mmol) were introduced through syringes. Three freeze-pump-thaw cycles have been performed to remove all the gases dissolved in the liquids. The reaction mixture was irradiated for 16 hours with Blue LEDs (450 nm, 573 mW) using the plate photoreactor. After this time, the reaction mixture was poured into a suspension of 8 equiv. of $NaBH_4$ in 2 mL of EtOH-CH_2Cl_2 (1: 1) at -30 °C (temperature kept with an acetone/dry ice bath). The reaction was stirred for 15 minutes at -30 °C, then the bath was allowed to warm up to room temperature in 30 minutes.
After 30 minutes, the reaction was quenched with a slowly addition of a 0.5 M solution of citric acid in MeOH (1 mL). After that all the volatiles were removed under reduced pressure and crude was purified by flash column chromatography on silica gel.

7 Unsuccessful results

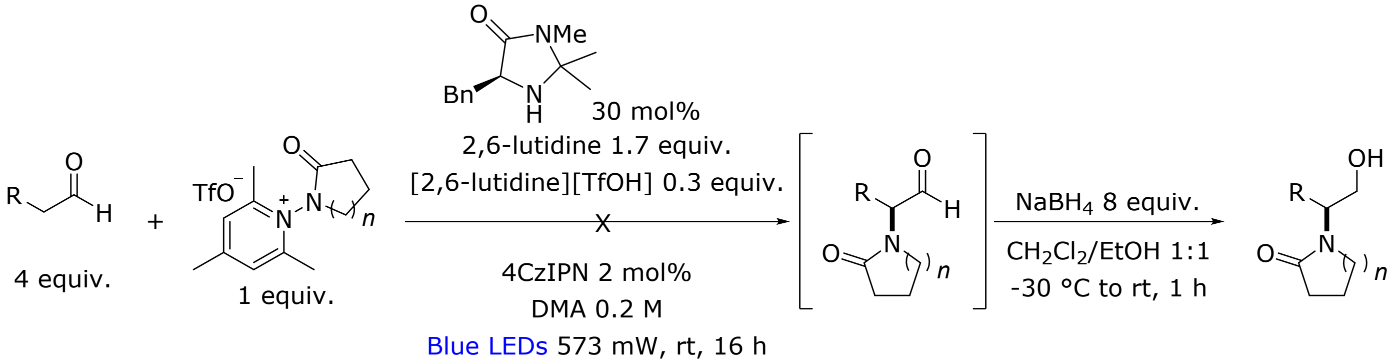

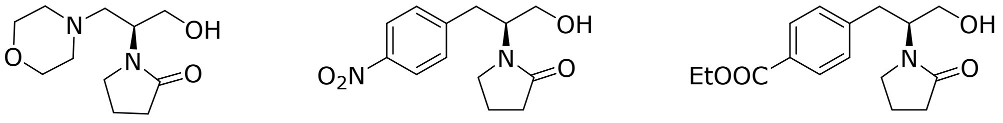

0% 0% 0%

8 Batch Telescoped Synthesis of Levetiracetam

8.1 Approach A

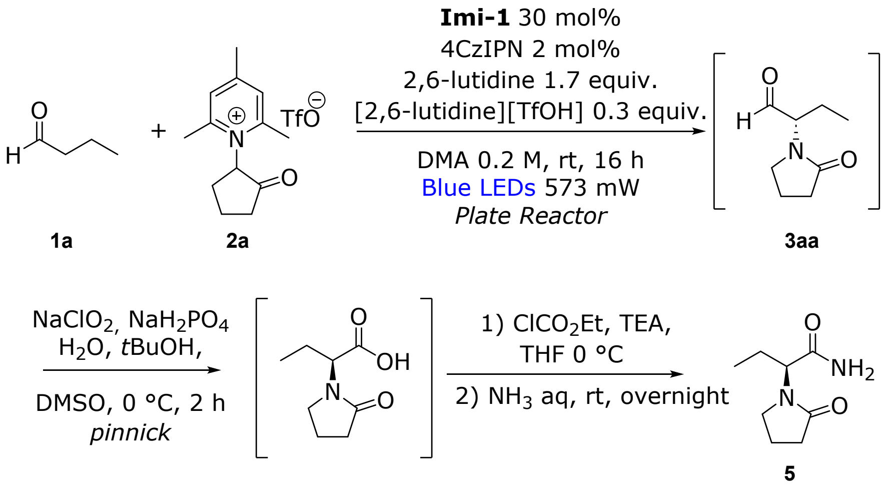

First-generation MacMillan catalyst **Imi-1** (0.54 mmol, 118 mg, 0.3 equiv.) the *N*-radical precursor **2a** (1.8 mmol, 638 mg, 1 equiv.), [2,6- lutidine][TfOH] (139 mg, 0.54 mmol, 0.3 equiv.), 4CzIPN (8 mg, 2 mol%) were added in three 7 mL vials, closed with an aluminium/PTFE septum and three cycles vacuum-nitrogen were performed. 9 mL of DMA (0.2 M) was added and then 2,6-lutidine (3.06 mmol, 365 µl, 1.7 equiv.) was added through syringe. Three freeze-pump-thaw cycles have been performed to remove all the gases dissolved in the liquids. Then, freshly distilled butyraldehyde **1a** (7.2 mmol, 650 µl, 4 equiv.) has been withdrawn with a syringe and added in the vial. The reaction mixture was stirred at room temperature, for 16 h under Blue light irradiation (573 mW) employing the Plate reactor. After this time, the reaction mixture was poured dropwise into a 250 ml flask charged with NaH_2PO_4 (72 mmol, 8.64 g, 40 equiv.), $NaClO_2$ (72 mmol, 6.5 g, 10 equiv.), *t*BuOH (22 mL, 0.33 M), DMSO (5.1 mL, 72 mmol, 40 equiv.) and water (48 mL, 0.15 M). The vial has been dipped in an ice bath at 0 °C for 30 minutes and then at room temperature for 2 h. The reaction was worked up adding NaOH 10% until pH 8. The aqueous phase has been extracted with EtOAc: the first organic phase was discharged, while the aqueous one was reacidified again by addition of HCl 1 M until pH 2. Extraction with EtOAc was performed (100 ml x 3). The second organic phase was dried over Na_2SO_4 and concentrated. A 50 mL two-necked flask, under nitrogen atmosphere has been charged with the reaction crude resulting from the previous work up. Dry THF (18 mL, 0.4 M) and NEt_3 (8.6 mmol, 1.2 mL, 4.8 equiv.) were added and the flask has been dipped in an ice bath at 0 °C. Then, ethyl chloroformate (7.9 mmol, 760 µL, 4.4 equiv.) was added through syringe and the reaction mixture was stirred under nitrogen at 0 °C (ice bath) for 1 h. After this time, NH_3 aq 30% (7.9 mmol, 1.23 ml, 4.4 equiv.) was added: the reaction was stirred at room temperature overnight. The day after, solid K_2CO_3 (1.5 equiv) was added. The reaction has been filtrated on cotton to remove solids and washed with EtOAc. The crude was concentrated and purified by column chromatography (CH_2Cl_2 -> CH_2Cl_2: MeOH 95:5). Levetiracetam **5** was isolated in 5% yield (16mg) with 80 % *e.e.* over three synthetic steps. All the analytical data are in agreement with literature.[16]
^{1}H NMR (300 MHz, $CDCl_3$) δ 6.42 (s, 1H), 5.71 (s, 1H), 4.45 (dd, *J* = 9.0, 6.8 Hz, 1H), 3.53 – 3.29 (m, 2H), 2.51 – 2.31 (m, 2H), 2.14 – 1.83 (m, 3H), 1.66 (ddt, *J* = 14.5, 9.1, 7.3 Hz, 1H), 0.89 (t, *J* = 7.4 Hz, 3H). **^{13}C NMR** (75 MHz, $CDCl_3$) δ 176.1, 172.4, 56.1, 43.9, 31.1, 21.0, 18.2, 10.8. **MS (APCI+) m/z:** $[M+H]^+$ Calcd for $C_8H_{14}N_2O_2$ 171.11; found 171.10. **R_f** = 0.18 (CH_2Cl_2:MeOH 95:5). **HPLC:** (OD-H,

isopropanol/*n*-hexane 10/90, flow rate = 1 mL/min, l = 254 nm) tR = 13.6 min (minor), 17.3 min (major).

8.2 Approach B

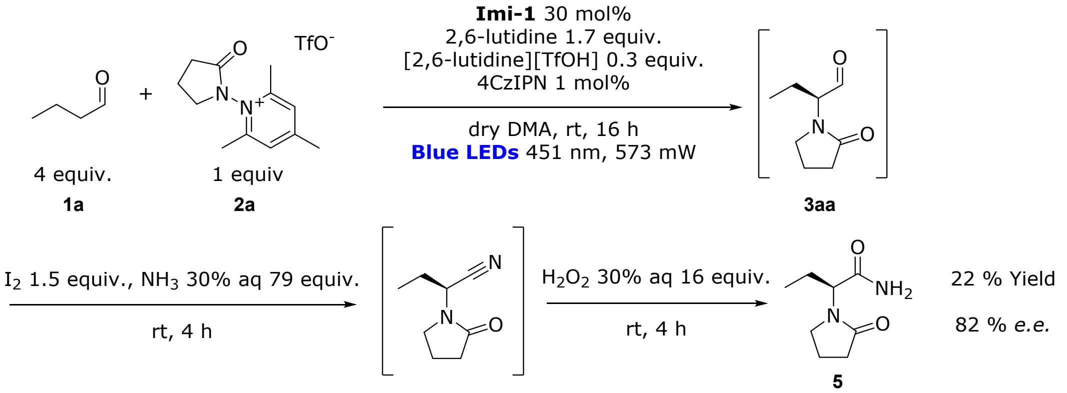

First-generation MacMillan catalyst **Imi-1** (0.54 mmol, 118 mg, 0.3 equiv.) the *N*-radical precursor **2a** (1.8 mmol, 638 mg, 1 equiv.), [2,6- lutidine][TfOH] (139 mg, 0.54 mmol, 0.3 equiv.), 4CzIPN (8 mg, 2 mol%) were added in three 7 mL vials, closed with an aluminium/PTFE septum and three cycles vacuum-nitrogen were performed. 9 mL of DMA (0.2 M) was added and then 2,6-lutidine (3.06 mmol, 365 µl, 1.7 equiv.) was added through syringe. Three freeze-pump-thaw cycles have been performed to remove all the gases dissolved in the liquids. Then, freshly distilled butyraldehyde **1a** (7.2 mmol, 650 µl, 4 equiv.) has been withdrawn with a syringe and added in the vial. The reaction mixture was stirred at room temperature, for 16 h under Blue light irradiation (573 mW) employing the Plate reactor. After this time, the vial caps were removed, the fractions combined, and the reaction mixture was subjected to high vacuum for 30 minutes to remove the excess butanal. 30% aqueous NH_3 (9 mL, 142 mmol, 79 equiv.) was added to the reaction mixture under stirring at room temperature. Then, iodine (685 mg, 2.7 mmol, 1.5 equiv.) was introduced, and the mixture was stirred for 4 hours at room temperature. After this period, H_2O_2 30% aq (2.94 mL, 29 mmol, 16 equiv.) was added dropwise, and the system was left under stirring for an additional 4 hours at room temperature. After this time, an excess of Na_2SO_3 was added to quench the reaction and the solvent was removed by rotary evaporation.

The crude product was dissolved in CH_2Cl_2, filtered through cotton to remove solid impurities, and subsequently washed with additional CH_2Cl_2. The filtrate was concentrated and purified by column chromatography on silica gel (CH_2Cl_2 → CH_2Cl_2:MeOH 95:5). Levetiracetam **5** was obtained in 22% yield (67.3 mg) with an enantiomeric excess of 82%, in a single continuous flow telescoped process. All analytical data were consistent with those reported in the literature.[16]

^{1}H NMR (300 MHz, $CDCl_3$) δ 6.42 (s, 1H), 5.71 (s, 1H), 4.45 (dd, *J* = 9.0, 6.8 Hz, 1H), 3.53 – 3.29 (m, 2H), 2.51 – 2.31 (m, 2H), 2.14 – 1.83 (m, 3H), 1.66 (ddt, *J* = 14.5, 9.1, 7.3 Hz, 1H), 0.89 (t, *J* = 7.4 Hz, 3H). **^{13}C NMR** (75 MHz, $CDCl_3$) δ 176.1, 172.4, 56.1, 43.9, 31.1, 21.0, 18.2, 10.8. **MS (APCI+) m/z:** $[M+H]^+$ Calcd for $C_8H_{14}N_2O_2$ 171.11; found 171.10. **R_f** = 0.18 (CH_2Cl_2:MeOH 95:5). **HPLC:** (OD-H, isopropanol/*n*-hexane 10/90, flow rate = 1 mL/min, l = 254 nm) tR = 13.6 min (minor), 17.3 min (major).

9 γ-*N* lactam addition to butyraldehyde in flow conditions

Butanal **1a** was selected as model substrate to study the optimization of γ-*N* lactam radical addition to *in situ* generated enamines under flow conditions.

The reaction mixture was prepared according to the General Procedure J.
When employing **PR-1**, the mixture is charged in a SGE syringe under nitrogen atmosphere, and a proper flow rate is maintained using a syringe pump. When employing **PR-2**, the mixture is stored under inert atmosphere and directly pumped from the volumetric flask using a *Syrris ASIA Syringe Pump*. The whole coil reactor is filled with the reaction mixture. The first reactor volume is discharged, then 1500 μl are collected and reduced to the corresponding alcohol according to the general procedure E.

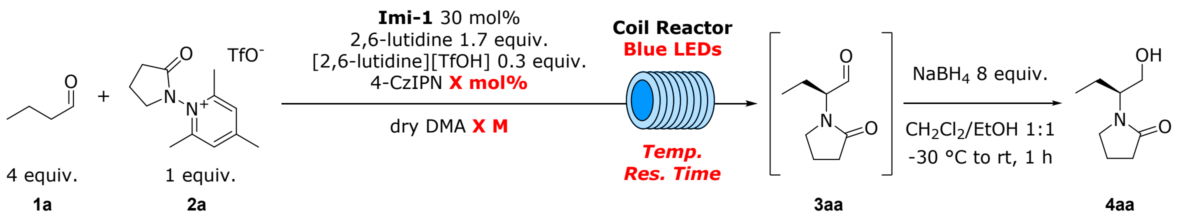

Entry	Photoreactor (Coil Reactor)	λ (nm)	T* (°C)	4-CzIPN (mol %)	2a [M]	Flow Rate (uL/min)	Residence Time (min)	4aa Yield (%)
1	PR-1 (CR-P-1)	460	R.T.	1	0.2	38	15	42
2		460	R.T.	1	0.2	19	30	53
3		460	R.T.	1	0.2	9.5	60	62
4		460	R.T.	1	0.1	19	30	43
5	PR-2 (CR-P-2)	450	20	2	0.1	33.3	30	25
6		450	20	2	0.1	16.7	60	22
7		450	40	2	0.1	33.3	30	15
8		450	20	2	0.2	33.3	30	44
9		450	20	1	0.2	66.7	15	19
10		450	20	1	0.2	33.3	30	45
11		450	20	1	0.2	16.7	60	60
12		450	20	1	0.5	33.3	30	40
13		420	20	1	0.2	33.3	30	27

*As previously mentioned, accurate temperature control is not achievable in **PR-1**, whereas it is possible in **PR-2**.
Table S9 *Optimization of the synthetic methodology under continuous flow conditions.*

The enantiomeric excess was evaluated on the corresponding naphthoyl derivative, and it was found to be constant and equal to the one achieved in batch conditions (89%).

10 Continuous Flow Telescoped Synthesis of Levetiracetam

The following reaction scheme describes the proposed synthetic approach for the continuous flow telescoped synthesis of Levetiracetam **5**.

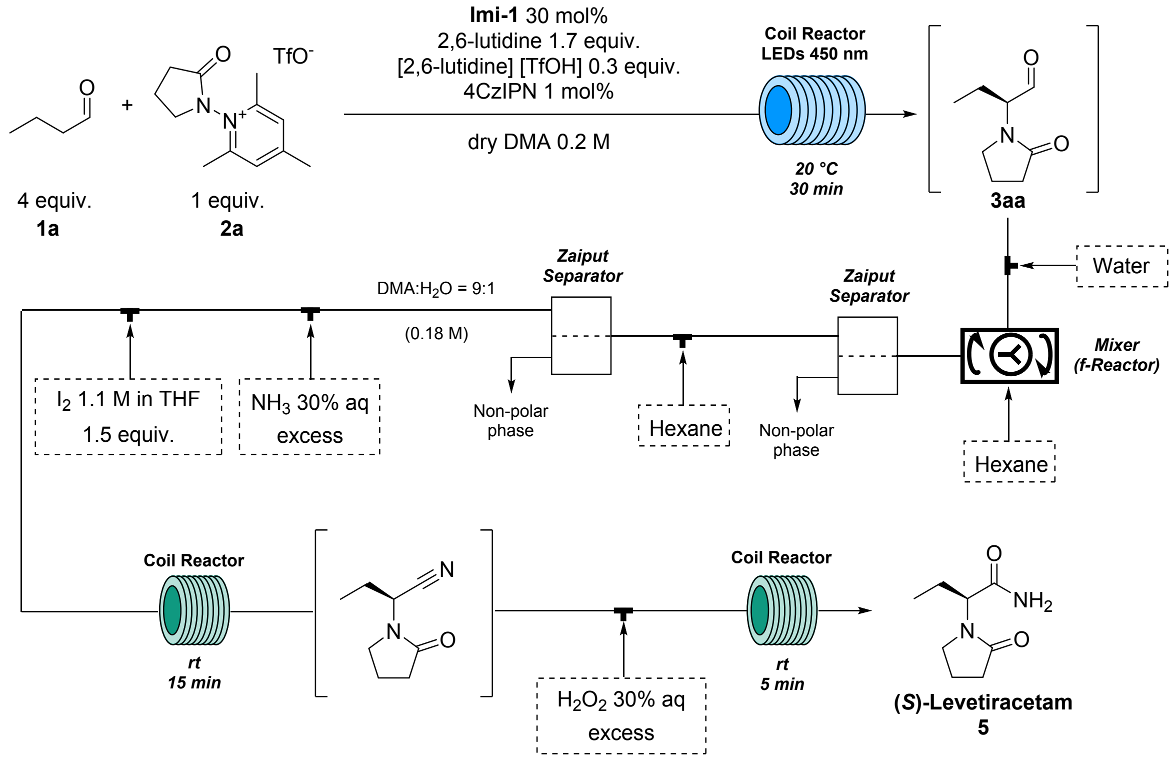

The optimization of the continuous flow telescoped synthesis of Levetiracetam **5** is divided in four stages: the first one is the photocatalytic step, which enables the direct, enantioselective, α-functionalization of butanal **1a** with the γ-lactam *N* radical. The second stage is focused on the removal of the excess of butanal **1a** using an *in-line* extraction system. The 2-(2-Oxo-pyrrolidin-1-yl)-butyraldehyde **3aa** is then oxidized in the third stage, yielding the corresponding nitrile, and finally in the fourth stage it is converted in the corresponding amide, which represents the target compound **5**.

10.1 Photocatalytic step

The reaction conditions outlined in Table S9 entry 10 were selected as optimal outcome to be employed in the continuous telescoped process. The 30 minutes residence time was selected in order to provide the best compromise between the overall yield and process productivity. Then, Syrris ASIA Premium System was employed because this device is commercially available and it enables a more reliable configuration, a greater scalability and an increased operational simplicity compared to a homemade system.

10.2 Extraction step

The second step is focused on the removal of the excess of butanal **1a** using an *in-line* extraction system.

A potential method for separating the two aldehydes is to take advantages of their different polarity and solubility in organic solvents, as we cannot rely on their different reactivity. Initially, the liquid-

liquid separation was studied in Batch in order to outline the best solvents. With the optimal conditions in hand, we then transfer the procedure in flow condition.

10.2.1 Batch extraction optimization

We first looked for a solvent able to form a biphasic mixture with DMA. The initial tests demonstrated that the most common organic solvents form a single-phase system with DMA. However, by adding a certain amount of water, DMA becomes polar enough to form a biphasic system, as it is shown in table S10.

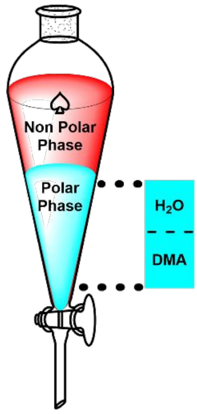

Solvent	% of H_2O in the Polar Phase needed
CH_2Cl_2	Single – Phase with DMA
AcOEt	Single – Phase with DMA
Hexane	> 5%
Et_2O	> 15%
MTBE	> 15%
iPr_2O	> 10%

Table S10 *biphasic system formation between DMA-H_2O and various organic solvents.*

To assess the outcome of the extraction, ^{1}H-NMR spectroscopy was employed as the analytical technique using Magritek Benchtop NMR, which offers solvent suppression functionalities. This is possible because the chemical shift of the two aldehydic proton differs by a few ppm, as shown in Figure S6.

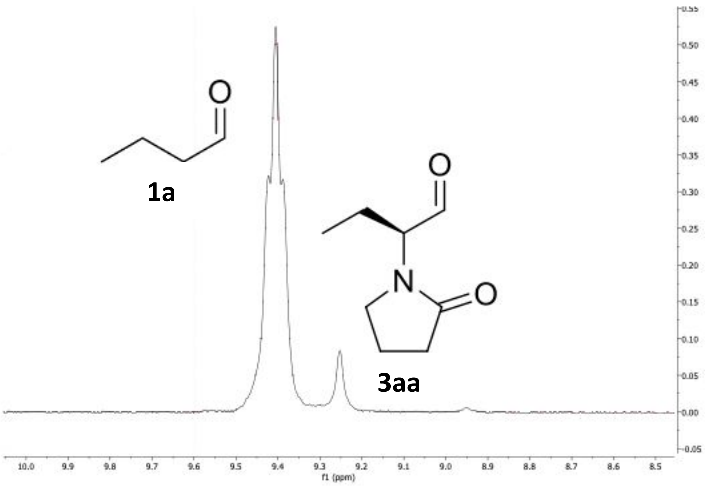

Figure S7 *Detail of the ^{1}H-NMR spectrum of [2-(2-Oxo-pyrrolidin-1-yl)-butyraldehyde] = 0.2 M, [butanal] = 0.6 M mixture.*

After having identified the solvents able to form a biphasic system with DMA, we tested them in the selective extraction of butyric aldehyde. 3 ml of a 2-(2-Oxo-pyrrolidin-1-yl)-butyraldehyde **3aa** (0.2 M) and butanal **1a** (0.6 M) mixture in DMA:H_2O = 95:5 was extracted with 3 ml of the corresponding organic solvent (Table S11).

Solvent	Presence of compound 3aa in the Non-Polar Phase
Hexane	No
Et_2O	Yes

**MTBE**	Yes
***i*Pr$_2$O**	Yes

Table S11 *Studies on the use of various organic solvents as extraction media.*

Hexane was selected as the optimal extraction solvent.
First, we determined the influence of the water content in the polar phase on the extraction outcome.
Ten solutions containing [2-(2-Oxo-pyrrolidin-1-yl)-butyraldehyde **3aa**] = 0.2 M and [butyraldehyde **1a**] = 0.6 M were made in DMA. Increasing amounts of H_2O (from 5% to 50% v/v respect to the total volume) were added, and each solution was extracted with an equal volume of hexane. The NMR spectra of the Polar (DMA + H_2O) and Non-Polar (hexane) phases were recorded. As expected, only butyraldehyde **1a** was detected in the Non-Polar phase.
In the DMA–H_2O phase, varying amounts of butyraldehyde **1a** and product **3aa** were observed. The spectra are shown in Figure S7, where the signal corresponding to butyraldehyde **1a** (left peak) was kept constant, while the signal of **3aa** (right peak) varied depending on the extraction efficiency.

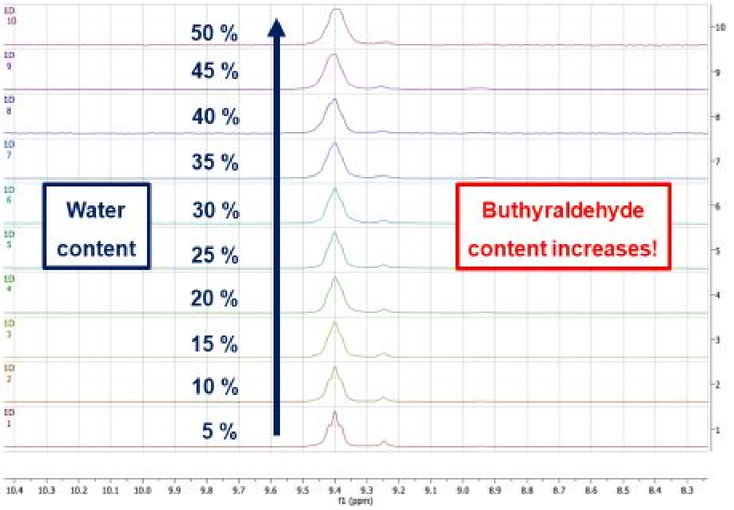

Figure S8 *Influence of the water content on the extraction efficiency.*

As shown in the figure S7, by increasing the amount of water in the DMA a worst separation was achieved, so the optimal amount of water is the minimum required to ensure the formation of a biphasic system: 5% v/v of H_2O on the total volume.
Once the optimal water content was identified, the next step was to determine the minimum amount of hexane required to complete the extraction in two stages. 3 ml of a 2-(2-Oxo-pyrrolidin-1-yl)-butyraldehyde **3aa** (0.2 M) and butanal **1a** (0.6 M) mixture in DMA:H_2O = 95:5 was extracted with increasing amount of hexane as depicted in Table S12.

**Hexane (ml)**	**Butyraldehyde 1a in the Polar Phase**	3aa **in the Non-Polar Phase**
**3 + 3**	Present in a greater amount respect to **3aa**	Absent
**15 +15**	Equimolar amount respect to **3aa**	Absent

**30 + 30**	Absent	Absent
**45 + 45**	Absent	Absent

Table S12 *Study on the amount of hexane required to selectively extract butyric aldehyde.*

Based on the results presented in Table S12, the optimal batch procedure for performing the extraction was successfully defined as it follows:
"In DMA, an amount of water equal to 5% (V/V respect to the final obtained solution) is added. A double extraction is performed: in each of them, an amount of hexane equal to 10 times the DMA+H_2O *volume is used."*

10.2.2 In-flow extraction optimization

Once the optimal batch procedure was defined, the extraction was studied under continuous flow conditions. According to the batch-optimized protocol, the extraction can be carried out using the following setup.

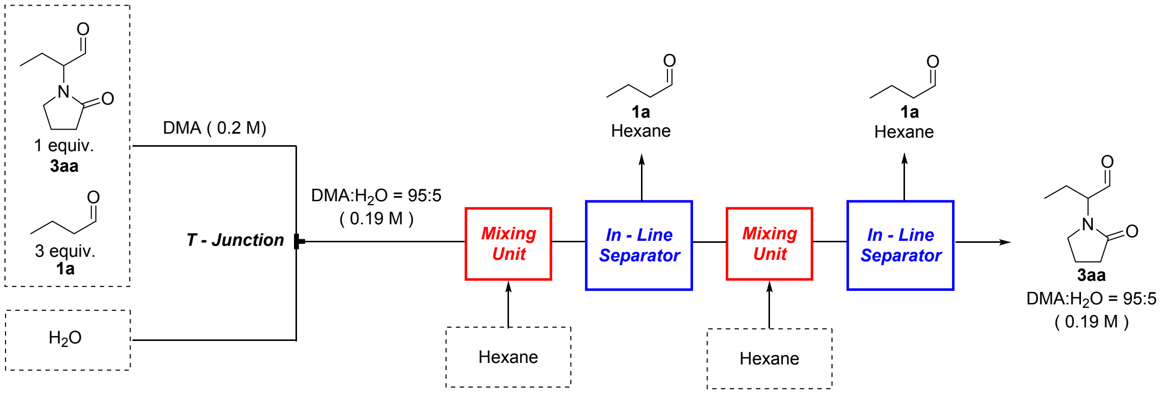

Scheme S2 *General scheme for the in-flow butyric aldehyde extraction.*

To optimize the extraction, different mixing units and in-line separators were evaluated. Specifically, for mixing, both a T-junction and the Asynt f-Reactor were tested. For in-line separation, two devices were considered: the SEP-10 separator from Zaiput Flow Technologies, equipped with an OB-900-S10 membrane, and the Asia FLLEX (Flow Liquid Liquid Extraction) module integrated into the Syrris ASIA Premium System.

The targeted separation was successfully adapted to continuous flow conditions employing the Asynt f-Reactor followed by the Zaiput separator for the first extraction, then the second volume of hexane was delivered through a T-junction and the mixture was separated with a second Zaiput separator as depicted in scheme S3. The connection coils between the first mixer and the Zaiput separator, and between the second T-junction and the second Zaiput unit, are identified as **CR-E-1**. The water content in the final polar phase was increased to 10%, as the greater polarity difference between the two phases ensures highly consistent and reproducible operation of the **SEP-10** separator.

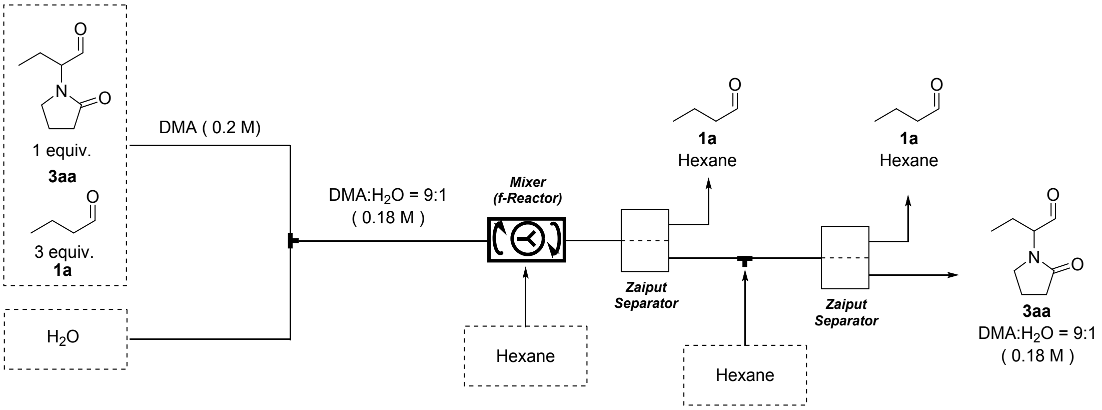

Scheme S3 *Final procedure for the in-flow butyraldehyde extraction.*

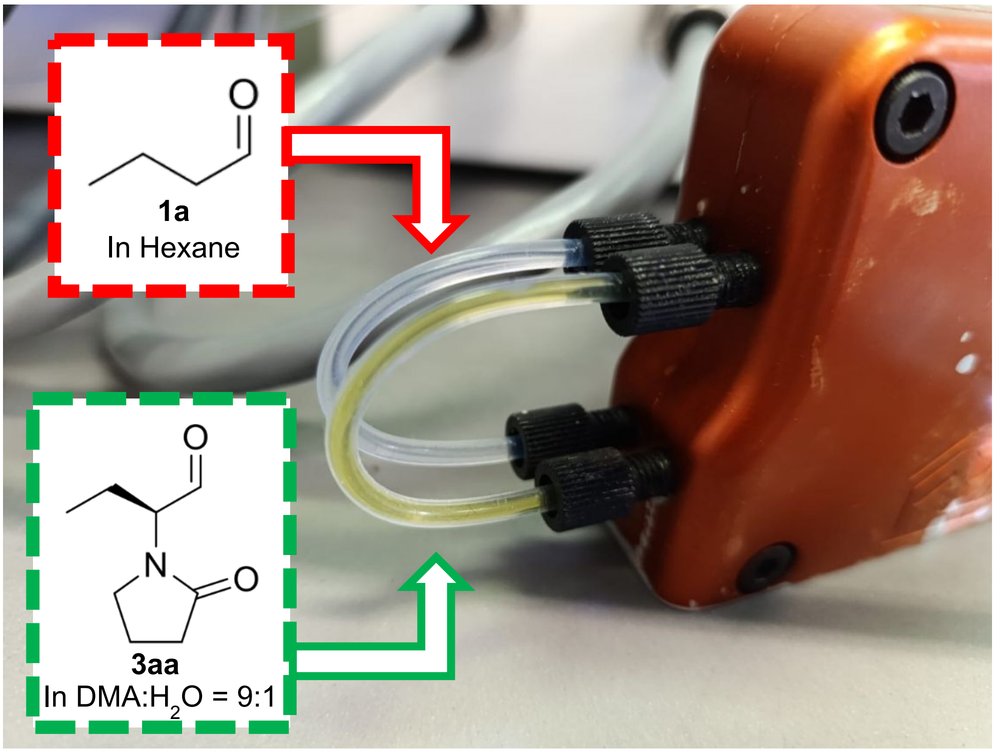

Figure S9 *Detail of the SEP-10 Zaiput Separator.*

10.3 Oxidation step

Following a batch literature procedure[18], we adapted the oxidation of aldehyde **3aa** to Levetiracetam **5** in continuous flow.
The study was divided into two phases: the optimization of the initial oxidation step to the nitrile, followed by the optimization of the subsequent conversion to the desired amide **5**. Tests were carried out using racemic 2-(2-oxo-pyrrolidin-1-yl)-butyraldehyde **3aa** as the starting aldehyde to minimize material waste.
CAUTION: Iodine is known to react with aqueous ammonia under certain conditions to form a black powder of $NI_3 \cdot NH_3$. When dry, this compound is highly sensitive, and it can explode upon exposure to shock, heat, or light. Although no incidents occurred during the handling of reagents in this study, it is strongly advised to avoid using excess amounts of reagents.[19]

*10.3.1 Aldehyde **3aa** Oxidation to Nitrile under continuous Flow Conditions*

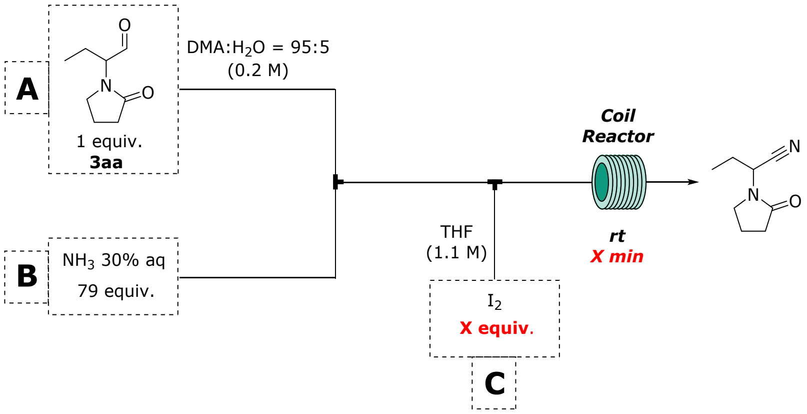

The aldehyde **3aa** (2 mmol, 310.4 mg, 1 equiv.) was dissolved in 10 ml of a DMA:H_2O = 95:5 mixture and the solution was loaded into a SGE syringe (*Syringe A*). A separate SGE syringe (*Syringe B*) was charged with 10 mL of 30% aqueous NH_3 solution (158 mmol, 79 equiv.). Finally, in a third SGE syringe (*Syringe C*), a solution of iodine (417.4 mg, 1 mmol, equiv. changing according to the operative flow rate) in THF (1.5 mL) was loaded.
The reaction was carried out under continuous flow conditions using various coil reactors, as outlined in Table S13. Appropriate residence times were established by adjusting the flow rates via syringe pump; the correspondence between residence time and flow rate is detailed in Table S13. While the molar flow rates of the aldehyde **3aa**: ammonia (*Syringe A*:*Syringe B*) were maintained in the ratio 1:79, the molar flow rate of iodine was varied to evaluate the effect of different equivalents, in accordance with the flow rate-equivalent relationship depicted in Table S13.
The collected fractions (1.0 ml) were quenched by adding 200 µl of a saturated $Na_2S_2O_3$ saturated solution, and the solvent was removed by rotary evaporation.
The reaction conversion was assessed via ^{1}H-NMR spectroscopy.

Entry	Coil Reactor	I_2 (equiv.)	Flow Rate (µL/min) A	B	C	t_R (min)	Conversion* (%)
1	CR-O-1	1.1	41	41	8.2	15	50
2		1.1	21	21	4.2	30	67
3		1.1	14	14	2.8	45	50
4	CR-O-2	1.3	37	37	8.7	15	85
5		1.3	18	18	4.3	30	89
6		1.3	12	12	2.9	45	90
7		1.5	36	36	9.9	15	> 99
8		1.5	18	18	4.9	30	> 99
9		1.5	12	12	3.3	45	> 99

*Reaction conversion determined by the ratio of starting material to product peaks by ^{1}H-NMR spectroscopy.

Table S13 *Aldehyde **3aa** Oxidation to Nitrile under continuous Flow Conditions*

*10.3.2 Aldehyde **3aa** Oxidation to Levetiracetam **5** under continuous Flow Conditions*

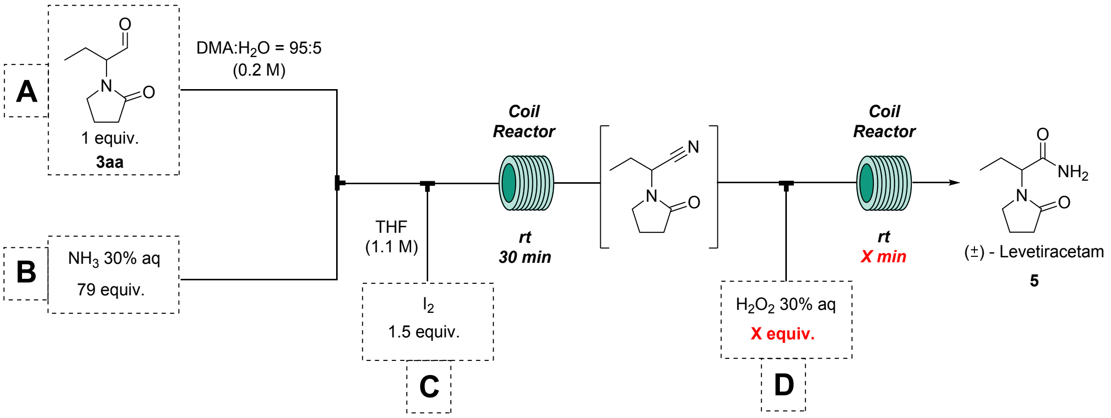

The aldehyde **3aa** (2 mmol, 310.4 mg, 1 equiv.) was dissolved in 10 ml of a DMA:H_2O = 95:5 mixture and the solution was loaded into a SGE syringe (*Syringe A*). A separate SGE syringe (*Syringe B*) was charged with 10 mL of 30% aqueous NH_3 solution (158 mmol, 79 equiv.). In a third SGE syringe (*Syringe C*), a solution of iodine (417.4 mg, 1 mmol, 1.5 equiv.) in THF (1.5 mL) was loaded. The reaction was carried out under continuous flow conditions using the conditions outlined in Entry 8 Table S13.

The reaction outcome was connected to different coil reactors placed in series (Table S14) and the further derivatization to Levetiracetam **5** was carried out. In a fourth SGE syringe (*Syringe D*), 1.5 ml of 30% aqueous H_2O_2 (14.7 mmol, equiv. changing according to the operative flow rate) were loaded.

Appropriate residence times were established by adjusting the flow rates via syringe pump; the correspondence between residence time and flow rate is detailed in Table S14. While the molar flow rates of the aldehyde:ammonia:iodine (*Syringe A*:*Syringe B*:*Syringe C*) were maintained in the ratio of 1:79:1.5, the molar flow rate of H_2O_2 was varied to evaluate the effect of different equivalents, in accordance with the flow rate-equivalent relationship depicted in Table S14.

The collected fractions (1.2 ml) were quenched by adding solid Na_2SO_3, and the solvent was removed by rotary evaporation. The reaction conversion was assessed via ^{1}H-NMR spectroscopy.

Entry	Coil Reactor	H_2O_2 (equiv.)	Flow Rate D (μL/min)	t_R (min)	Conversion* (%)
1		16	5.9	30	96
2	CR-O-4	25	9.2	30	94
3		34	12.6	30	97
4		16	5.9	15	90
5	CR-O-5	25	9.2	15	92
6		34	12.6	15	98
7		16	5.9	5	95
8	CR-O-5	25	9.2	5	95
9		34	12.6	5	98

*Reaction conversion determined by the ratio of starting material to product peaks by NMR spectroscopy.

Table S14 *Aldehyde **3aa** Oxidation to Levetiracetam **5** under continuous Flow Conditions*

The oxidation was succesfully adapted to continuous flow conditions, according to the following reaction scheme.

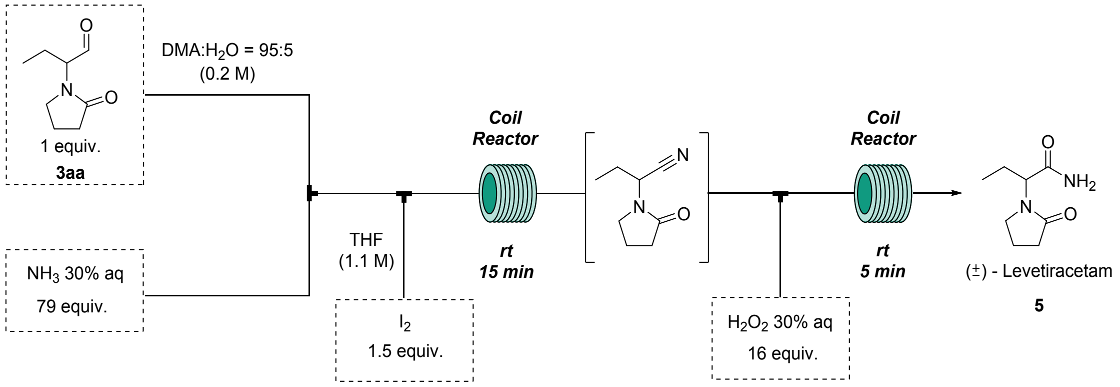

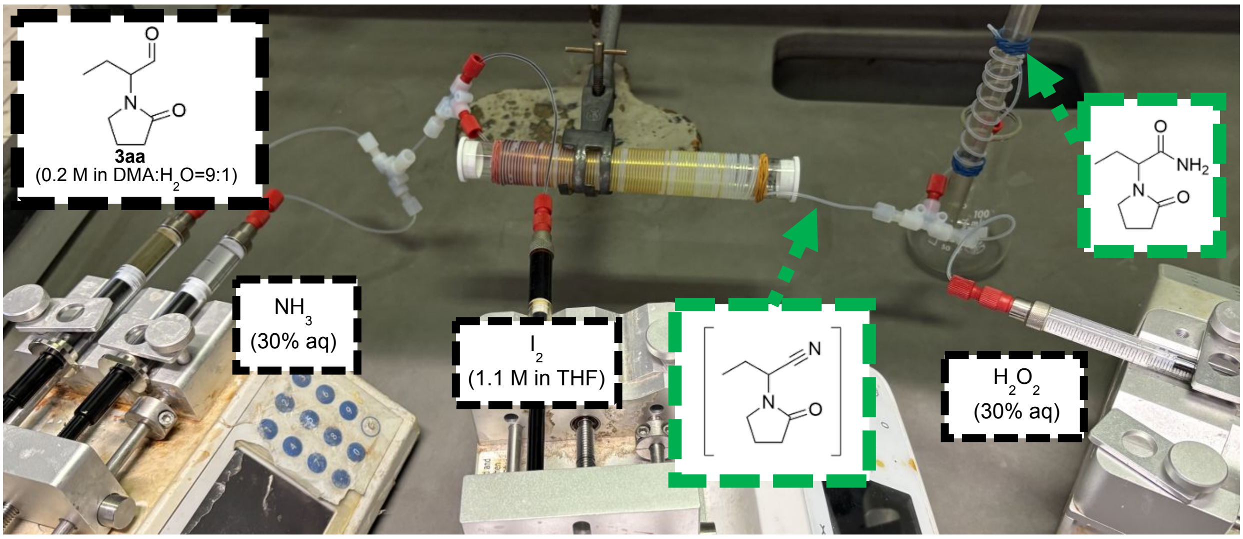

5

Figure S10 *Detail of the Continuous Flow Oxidation of 2-(2-Oxo-pyrrolidin-1-yl)-butyraldehyde **3aa** to racemic Levetiracetam **5**.*

10.4 Continuous Flow Telescoped Synthesis of Levetiracetam

With the data obtained from the previous studies in hand, it was possible to carry out the final continuous flow telescoped process. A residence time of 30 minutes was selected for the photochemical step (Table S9, Entry 10), in order to provide an optimal compromise between the overall yield and process productivity.

CAUTION: Iodine is known to react with aqueous ammonia under certain conditions to form a black powder of $NI_3 \cdot NH_3$. When dry, this compound is highly sensitive, and it might explode upon exposure to shock, heat, or light. Although no incidents occurred during the handling of reagents in this study, it is strongly advised to avoid using excess amounts of reagents.[19]

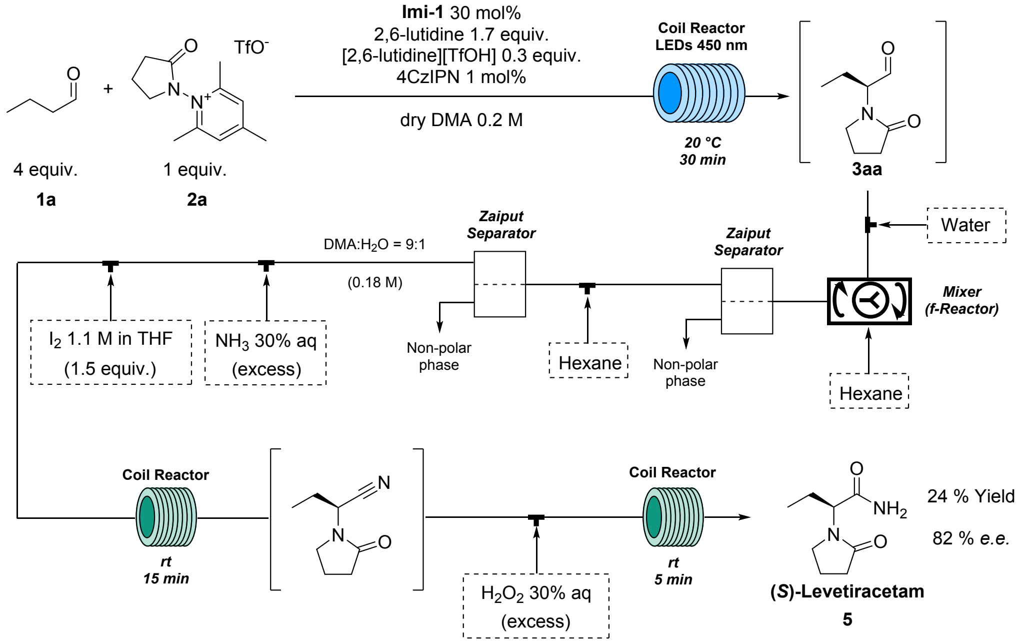

Into a 50 ml flask (*Flask A*) under inert atmosphere, the nitrogen radical precursor **2a** (1065 mg, 1 equiv., 3 mmol), 4-CzIPN (23.6mg, 1 mol%, 0.03 mmol), the organocatalyst **Imi-1** (196.5 mg, 30 mol%, 0.9 mmol) and [2,6-lutidine][TfOH] (231.5 mg, 0.3 equiv., 0.9 mmol) were introduced. The flask was sealed, and three nitrogen-vacuum cycles were done. DMA (15 ml, 0.2 M), 2,6-lutidine (583 ul, 1.7 equiv., 4.5 mmol) were introduced through syringes. Three freeze-pump-thaw cycles have been performed to remove all the gases dissolved in the liquids. Finally, the degassed daily freshly distilled butyric aldehyde **1a** (1068 μl, 4 equiv., 12 mmol) was added.

In four separate flasks, a sufficient amount of distilled water (5 ml) (*Flask B*), hexane (40 ml) (*Flask C*), 30% aqueous H_2O_2 (7 ml, 16 equiv.) (*Flask D*) and 30% aqueous NH_3 (15 ml, 79 equiv.) (*Flask E*) was introduced to supply the process for the entire duration. Finally, in a 25 ml SGE syringe (*Syringe F*), a solution of iodine (1.4 g, 1.5 equiv., 5.5 mmol) in THF (5 mL) was introduced.

The reaction was carried out under continuous flow conditions, using the Syrris ASIA Premium System to control the overall process and one external syringe pump. Size and characteristic of the different employed coil reactors are depicted in **CR-CSF-1** (Chapter 3 of the Supporting Information). Appropriate residence times were established by adjusting the flow rates through Syrris ASIA control system: all the employed flow rates are described in Table S15.

The outcoming flow was collected in a flask containing solid Na_2SO_3 to quench the reaction. 22 mL (corresponding to 1.6 mmol of limiting reagent **2a**) were collected and the solvent was removed by rotary evaporation.
The crude product was dissolved in CH_2Cl_2, filtered through cotton to remove solid impurities, and subsequently washed with additional CH_2Cl_2. The filtrate was concentrated and purified by column chromatography (CH_2Cl_2 → CH_2Cl_2:MeOH 95:5). Levetiracetam **5** was obtained in 24% yield (65.2 mg) with an enantiomeric excess of 82%, in a single continuous flow telescoped process. All analytical data were consistent with those reported in the literature.[16]
^{1}H NMR (300 MHz, $CDCl_3$) δ 6.42 (s, 1H), 5.71 (s, 1H), 4.45 (dd, *J* = 9.0, 6.8 Hz, 1H), 3.53 – 3.29 (m, 2H), 2.51 – 2.31 (m, 2H), 2.14 – 1.83 (m, 3H), 1.66 (ddt, *J* = 14.5, 9.1, 7.3 Hz, 1H), 0.89 (t, *J* = 7.4 Hz, 3H). **^{13}C NMR** (75 MHz, $CDCl_3$) δ 176.1, 172.4, 56.1, 43.9, 31.1, 21.0, 18.2, 10.8. **MS (APCI+) m/z:** $[M+H]^+$ Calcd for $C_8H_{14}N_2O_2$ 171.11; found 171.10. **R_f** = 0.18 (CH_2Cl_2:MeOH 95:5). **HPLC:** (OD-H, isopropanol/*n*-hexane 10/90, flow rate = 1 mL/min, l = 254 nm) tR = 13.6 min (minor), 17.3 min (major).

Coil Reactor	Flask	Content	Total Amount (ml)	Flow Rate (µL/min)	Molar Flow Rate (nmol/min)	Equiv.
	A	Reaction Mixture in DMA	15	35	7	1
	B	Water	5	5	-	-
**CR-CSF-1**	C	Hexane	40	400 + 400	-	-
	D	30% aq H_2O_2	7	11.4	112	16
	E	30% aq NH_3	15	35	553	79
	F	I_2 in THF	5	9.5	10	1.5

Table S15 *Operative details of the continuous flow telescoped process.*

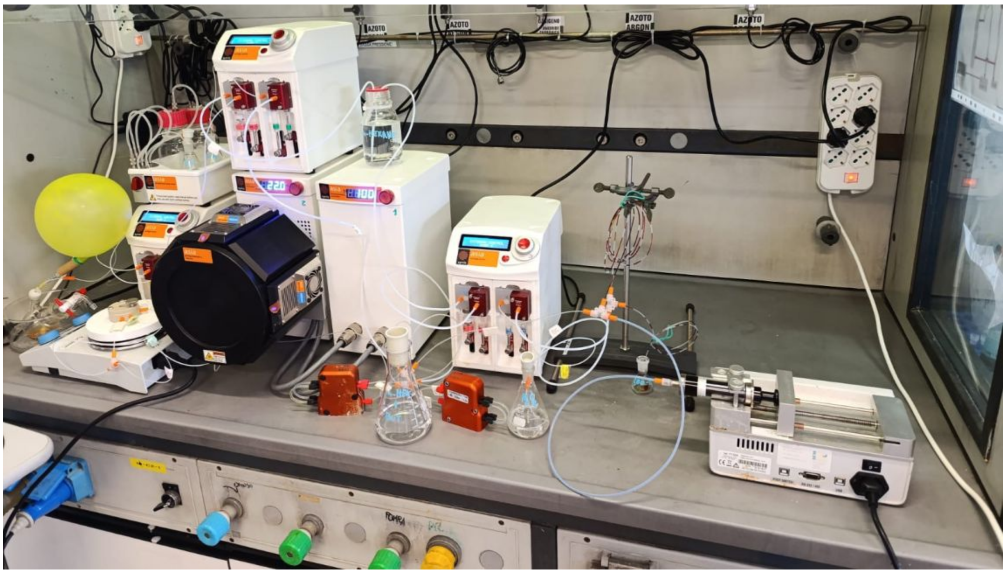

Figure S11 *Continuous Flow Telescoped Synthesis of Levetiracetam under work, with the Syrris ASIA Premium Syste.*

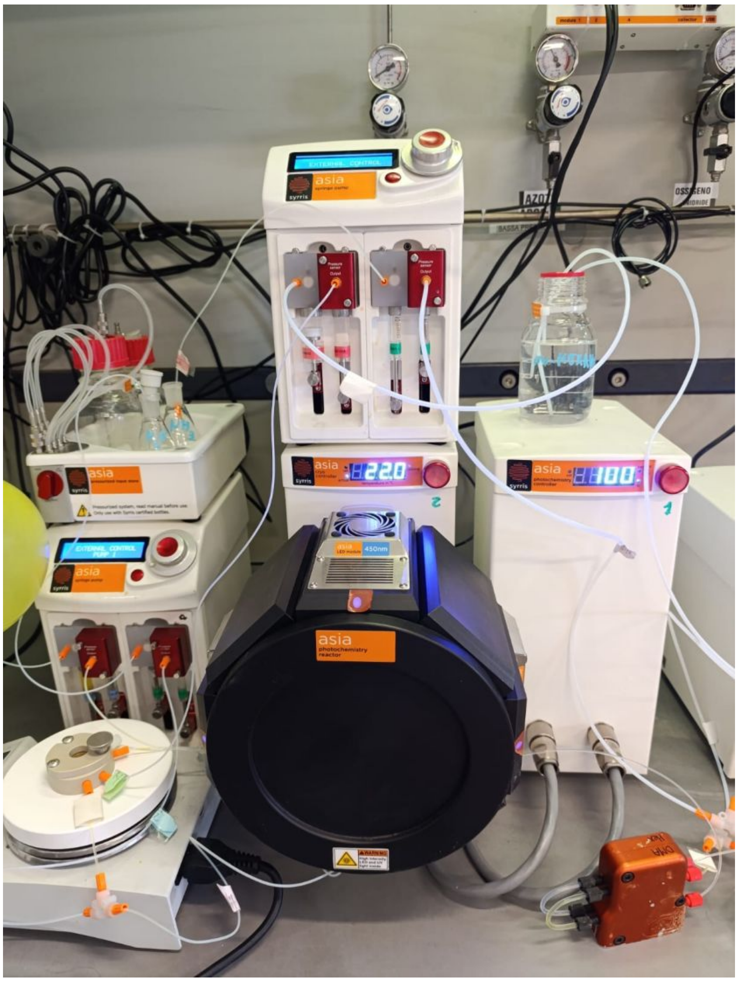

Figure S12 *Detail of the operative Syrris ASIA Photoreactor.*

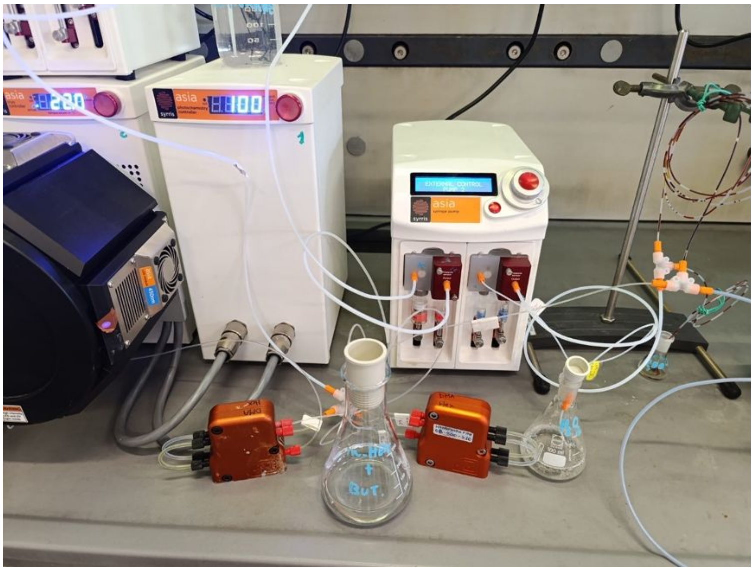

Figure S13 *Detail of the extraction stage.*

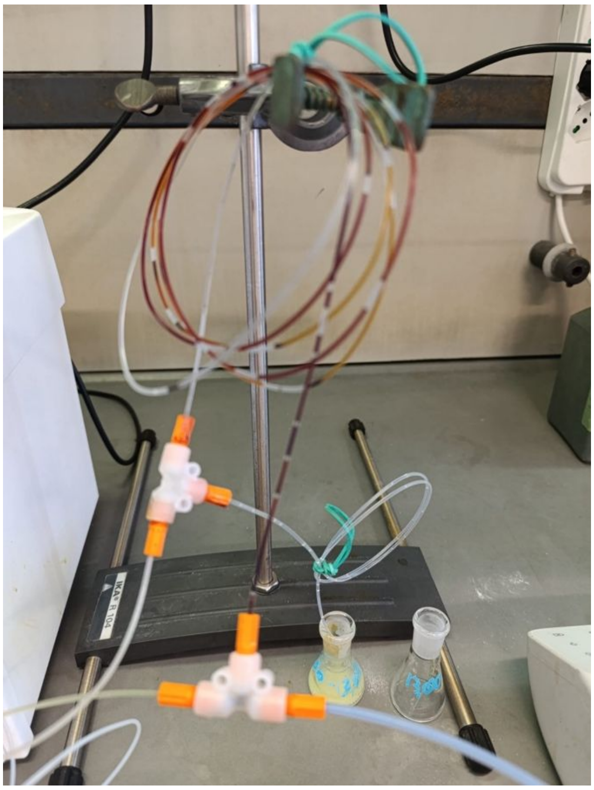

Figure S14 *Detail of the oxidation stage.*

10.5 Productivity and Space Time Yield Calculations

The performances of the experiments under continuous conditions were evaluated in terms of productivity and space-time yield and compared to the same values calculated for the batch reactions. Productivity is defined as the mmol of product per hour provided by the system:

$$Productivity = \frac{mmol\ limiting\ reagent\ \cdot yield}{time\ (h)}$$

The space-time yield (STY) instead, is defined as the mmol of product per hour, per unit of volume:

$$STY = \frac{mmol\ limiting\ reagent\ \cdot yield}{time\ (h)\ \cdot volume\ (ml)}$$

Time in batch condition: reaction time. Time in flow condition: time to flow the same mmol of limiting agent.

As shown in Table S16. Productivity and Space Time Yield for the enantioselective α addition of N lactam radicals to aldehydes were calculated.

Entry	5 Yield (%)	Productivity ($mmolh^{-1}$)	Rel. factor	STY ($mmolh^{-1}ml^{-1}$)	Rel. Factor
**Batch**	22	0.017	1	0.0079	1
**Flow**	24	0.1	6	0.037	5

Table S1 *Batch conditions: Approach B in Chapter 7.2. Flow Conditions: Continuous Flow Telescoped Synthesis in Chapter 9.4.*

11 Mechanistic Investigations

11.1 Stern-Volmer Analysis

SOLUTION A: 1.0 mg of 4CzIPN (1.27 μmol) were dissolved in 0.98 mL of DMA, then 150 μl of this solution were diluted with 20 ml of DMA in order to obtain a standard photocatalyst solution ~10 μM.

SOLUTION B: 141.1 mg of nitrogen radical precursor **2a** (0.398 mmol) were dissolved into 4 ml of Solution A. Then, after recording the photoluminescence and the lifetime spectra of solution A, an increasing amount of Solution B was added in order to study the changing of the quencher concentration vs the I_0/I and τ_0/τ.

Photoluminescence

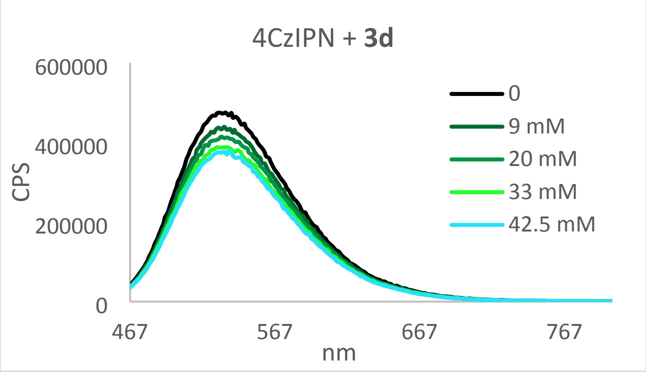

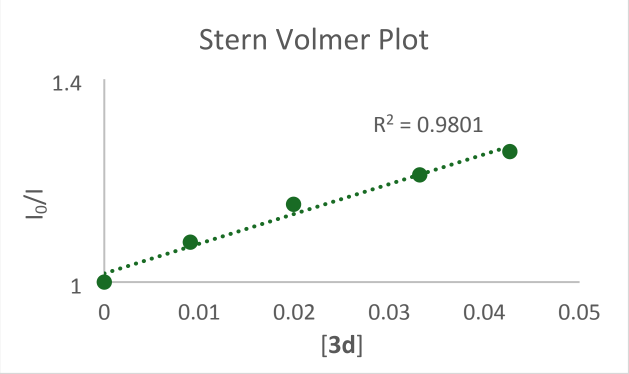

Lifetime

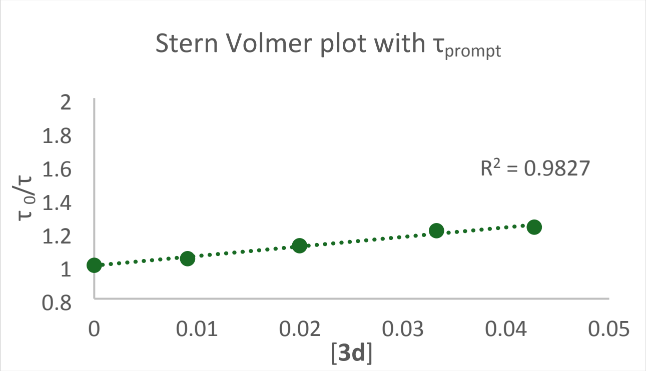

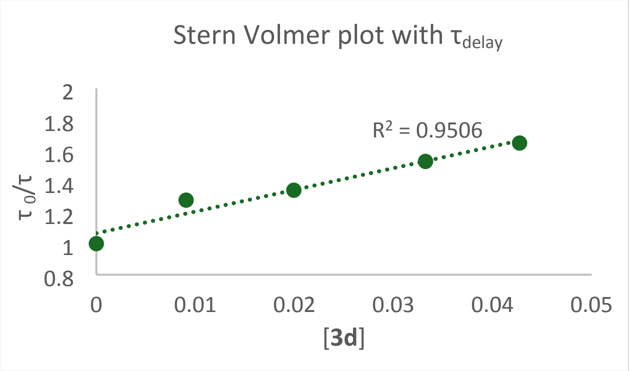

11.2 ^{1}H-NMR Enamine Formation Studies

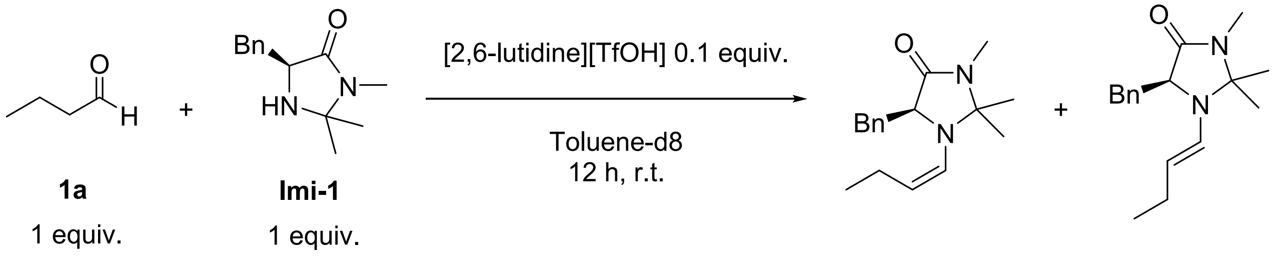

In a closed vial, **Imi-1** (65.5 mg, 1 equiv., 0.3 mmol), butyraldehyde **1a** (27 µL, 1 equiv., 0.3 mmol), and [2,6-lutidine][TfOH] (7.7 mg, 0.1 equiv., 0.03 mmol) were introduced, and dissolved in 0.5 mL of Toluene-*d8*. The reaction mixture was stirred at room temperature overnight.

After 12 hours, the ^{1}H-NMR of the crude reaction confirmed the enamine formation in a 20% molar ratio with respect to **Imi-1** catalyst. Both the two stereoisomers E and Z were detected in equal amount.

^1H NMR (400 MHz, $CDCl_3$);

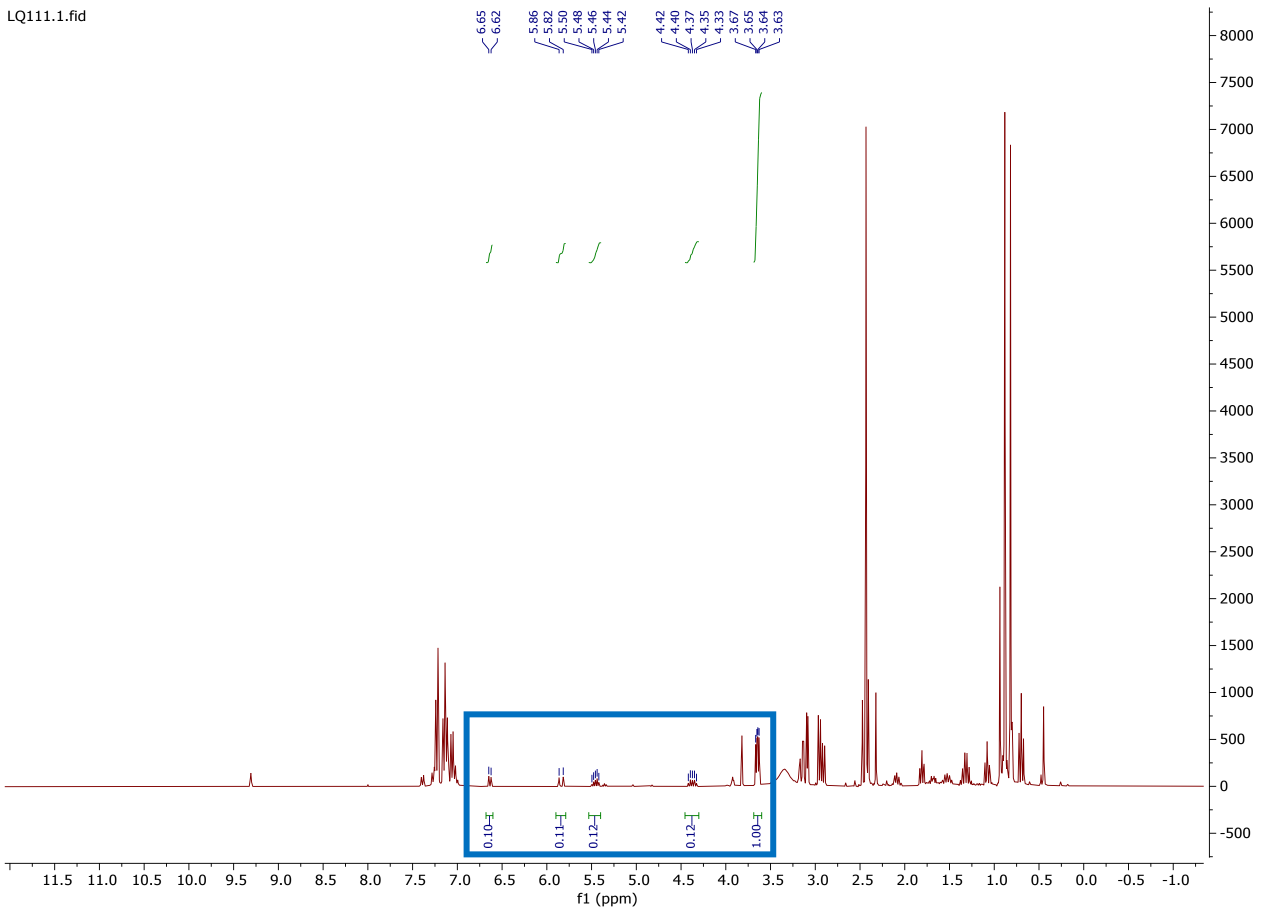

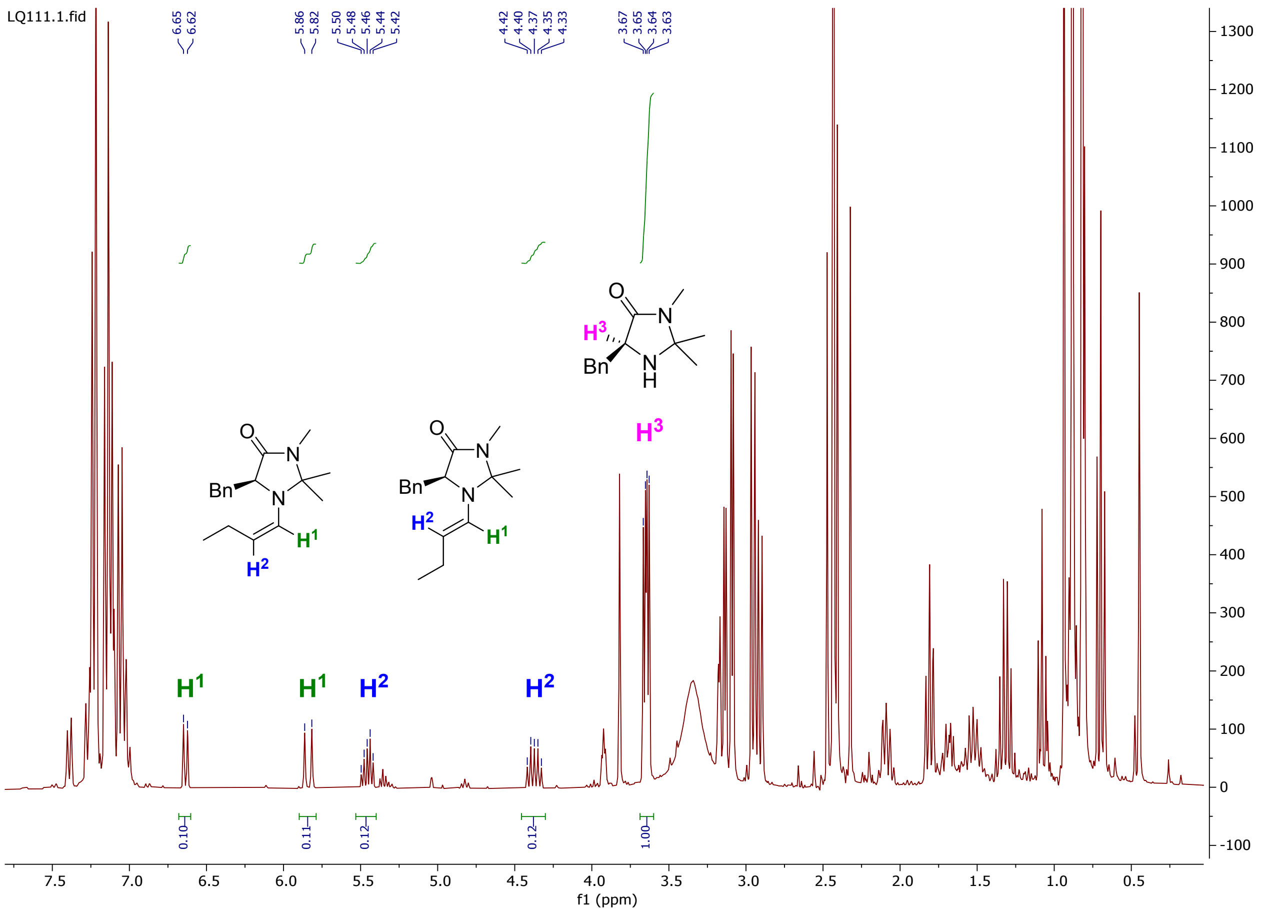

12 DFT Calculations

12.1 Computational details

Density functional theory (DFT) calculations were performed using the Gaussian 16 program and the results were produced with GaussView 6.0. For the calculation of global and local electrophilicity index, B3LYP functional was used and the geometries of studied radicals were optimized at the UB3LYP/6-311+G(d,p) level of theory, followed by frequency calculations at the same level. Each optimized radical geometry has been used to calculate the adiabatic energy of the corresponding cation and ion. The Hirshfield charges have also been calculated on each structure.

To extract the values of the Hirshfield charges, the .fchk file generated by Gaussian has been analysed in MultiWFN 3.3.9.

The Fukui indices were calculated with the following equations:

$$f^{+} = q(N) - q(N+1)$$

$$f^{-} = q(N-1) - q(N)$$

Where q(N) is the Hirshfield charge of the radical, q(N+1) is the anion, and q(N-1) is the cation.

12.2 Optimized Structures, Cartesian Coordinates, Fukui indices, and Energies

5-terms cycle

	Lactam$^+$	Lactam$^\cdot$	Lactam$^-$	
Electronic Energy (EE)	-285,65500	-286,03732	-286,11816	Hartree
EE + Zero-point Energy		-285,94170		Hartree
EE + Thermal Free Energy Correction		-285,97136		Hartree

	Coordinates			Hirshfield charges			Fukui indeces	
	X	Y	Z	q(N+1)	q(N)	q(N-1)	f^+	f^-
C	1,26462	-0,84251	0,10707	-0,05978	-0,00650	0,06688	0,05328	0,07337
C	-0,88234	-0,03792	-0,00597	0,06080	0,16063	0,22892	0,09984	0,06829
C	1,40771	0,67301	-0,18199	-0,07463	-0,04902	-0,01500	0,02561	0,03403
H	1,89775	-1,49043	-0,50691	-0,01397	0,04995	0,13273	0,06392	0,08279
H	1,53210	-1,07685	1,15282	-0,02642	0,05472	0,15502	0,08113	0,10030
H	2,20014	1,13815	0,40542	-0,00662	0,03743	0,07802	0,04406	0,04059
H	1,64348	0,82567	-1,23830	0,00371	0,03564	0,07992	0,03193	0,04427
N	-0,11868	-1,21288	-0,05760	-0,36194	-0,09235	0,14261	0,26959	0,23496
O	-2,09880	-0,05325	-0,05328	-0,44769	-0,23048	-0,02998	0,21721	0,20050
C	0,00520	1,20078	0,13929	-0,08184	-0,05434	-0,01768	0,02750	0,03667
H	-0,07709	1,55421	1,17312	0,00574	0,04664	0,08611	0,04090	0,03948
H	-0,34635	2,00527	-0,50707	0,00436	0,04810	0,09262	0,04374	0,04452

6-terms cycle

	$Lactam^+$	$Lactam^\cdot$	$Lactam^-$	
Electronic Energy (EE)	-324,99770	-325,36449	-325,41878	Hartree
EE + Zero-point Energy		-325,23924		Hartree
EE + Thermal Free Energy Correction		-325,27009		Hartree

	Coordinates			Hirshfield charges			Fukui indeces	
	X	Y	Z	q(N+1)	q(N)	q(N-1)	f^+	f^-
C	-0,88765	-1,31604	0,19377	-0,05148	-0,00171	0,06096	0,04977	0,06267
C	-1,75234	-0,05742	-0,07265	-0,07599	-0,03945	0,01836	0,03654	0,05781
C	-0,99886	1,21579	0,32075	-0,05584	-0,04148	-0,01488	0,01436	0,02659
C	0,38360	1,25801	-0,35934	-0,07967	-0,04990	-0,00153	0,02977	0,04837
H	-1,57750	2,10276	0,04771	0,00183	0,03840	0,09344	0,03658	0,05504
H	-2,01115	-0,02834	-1,13596	0,00164	0,03397	0,07227	0,03233	0,03829
H	-2,68721	-0,15573	0,48649	-0,01522	0,04075	0,09259	0,05597	0,05184
H	-0,74169	-1,42319	1,27998	-0,02203	0,04297	0,11404	0,06500	0,07107
H	-1,38470	-2,21567	-0,17295	-0,00702	0,04036	0,09072	0,04738	0,05036
H	0,26169	1,37878	-1,44171	0,00888	0,04243	0,07557	0,03356	0,03314
H	1,00716	2,07626	0,00417	-0,00475	0,04543	0,09757	0,05017	0,05215
H	-0,86786	1,24668	1,40838	0,00962	0,03270	0,06037	0,02308	0,02767
C	1,12486	-0,05014	-0,09903	0,09254	0,16648	0,21589	0,07395	0,04941
O	2,24868	-0,12369	0,35412	-0,40245	-0,27208	-0,14590	0,13037	0,12618
N	0,39916	-1,18475	-0,45859	-0,39813	-0,07853	0,17064	0,31960	0,24917

7-terms cycle

	$Lactam^+$	$Lactam^\cdot$	$Lactam^-$	
Electronic Energy (EE)	-364,33367	-364,68702	-364,73106	Hartree
EE + Zero-point Energy		-364,53305		Hartree
EE + Thermal Free Energy Correction		-364,56605		Hartree

	Coordinates			Hirshfield charges			Fukui indeces	
	X	Y	Z	q(N+1)	q(N)	q(N-1)	f^+	f^-
C	-0,4544	-1,62565	0,08018	-0,045869	-0,001164	0,055392	0,044705	0,056556
C	-1,76552	-0,80414	-0,007653	-0,076028	-0,038785	0,017115	0,037243	0,0559
C	-1,65725	0,646849	0,479214	-0,054787	-0,045988	-0,026375	0,008799	0,019613
C	-0,71068	1,534681	-0,351975	-0,058343	-0,046424	-0,028606	0,011919	0,017818
C	0,781972	1,378894	-0,022525	-0,074989	-0,049759	-0,021965	0,02523	0,027794
H	-0,15445	-1,7323	1,131442	-0,01725	0,034829	0,088087	0,052079	0,053258
H	-2,10953	-0,81447	-1,046848	0,000691	0,032755	0,067115	0,032064	0,03436
H	-1,35277	0,666404	1,533774	0,008969	0,02799	0,049664	0,019021	0,021674

H	-0,88078	1,343526	-1,417029	0,009432	0,028596	0,046374	0,019164	0,017778
H	-0,63969	-2,62002	-0,332929	-0,012935	0,043188	0,097332	0,056123	0,054144
H	-2,51484	-1,34108	0,583294	-0,023177	0,038364	0,093428	0,061541	0,055064
H	-2,66127	1,082203	0,446979	0,002679	0,034149	0,081254	0,03147	0,047105
H	-0,97239	2,583992	-0,185965	-0,00349	0,034227	0,069856	0,037717	0,035629
H	1,388477	1,971372	-0,716465	0,01177	0,049559	0,088259	0,037789	0,0387
H	1,002813	1,764841	0,97797	0,001747	0,04291	0,078377	0,041163	0,035467
C	1,35311	-0,03479	-0,055478	0,111864	0,17059	0,217878	0,058726	0,047288
O	2,441721	-0,31625	0,404778	-0,390724	-0,27728	-0,117891	0,113444	0,159389
N	0,582467	-0,9928	-0,706147	-0,387711	-0,077341	0,144988	0,31037	0,222329

12.3 Electrophilicity Calculation

To calculate the value of electrophilicity, the Electronic Energy (EE) has been used. Here are reported the equations to calculate the electrophilicity indices:

$$IP = Ionization\ Potential = E^{+} - E^{0}$$

$$EA = Electronic\ Affinity = E^{0} - E^{-}$$

$$\mu = Electronic\ Chemical\ Potential = -\frac{EA + IP}{2}$$

$$\eta = Chemical\ Hardness = \frac{IP - EA}{2}$$

$$\omega^{+} = Global\ Electrophilicity = \frac{\mu^{2}}{2\eta}$$

$$\omega_{X}^{+} = Local\ Electrophilicity\ on\ atom\ X = \omega^{+} f_{X}^{+}$$

	IP (eV)	EA (eV)	m (eV)	h (eV)	w^{+} (eV)	w_{N}^{+} (eV)
5- terms cycle	10,40341	2,199878	-6,30164	4,101764	4,840685	1,305
6- terms cycle	9,980977	1,477252	-5,72911	4,251862	3,859809	1,233591
7- terms cycle	9,615228	1,198308	-5,40677	4,20846	3,47314	1,077959

13 NMR Spectra

(2*S*,5*S*)-2-benzyl-2,3,5-trimethylimidazolin-4-one (Imi-3) ^{1}H NMR (400 MHz, $CDCl_3$); ^{13}C NMR (75 MHz, $CDCl_3$).

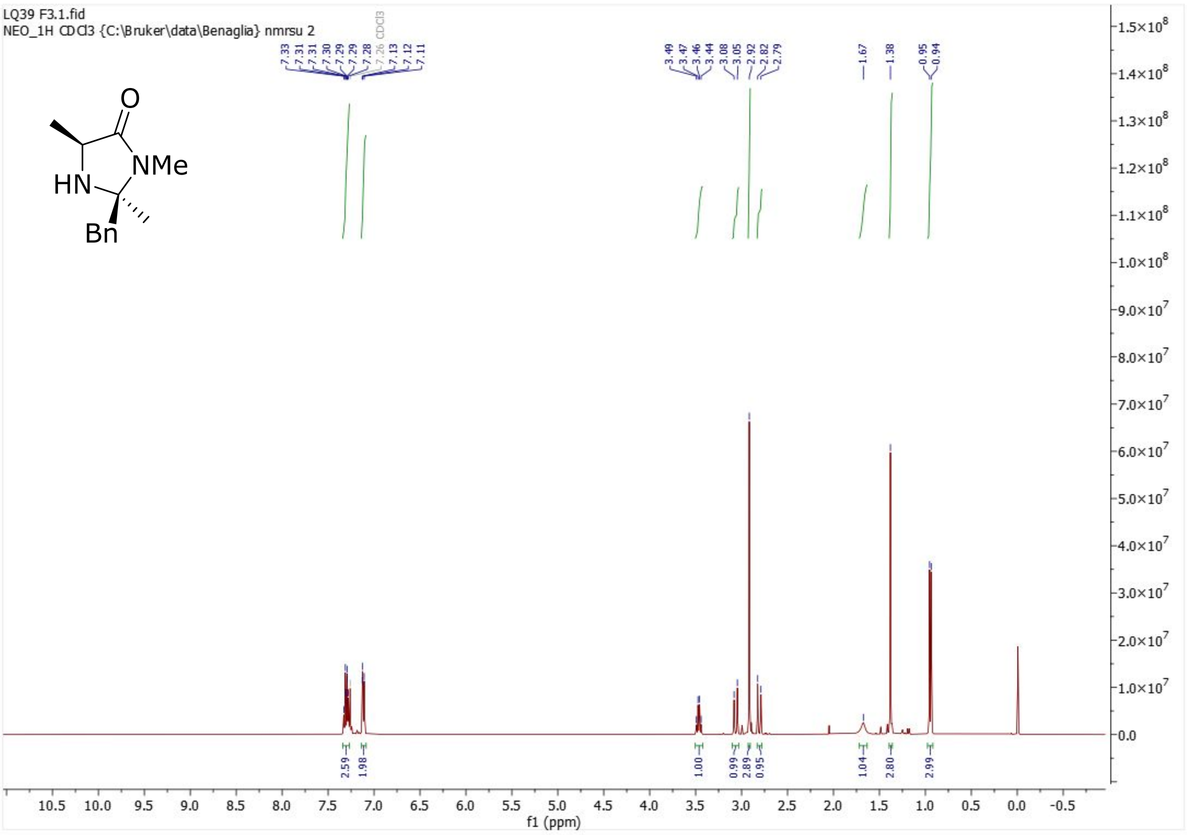

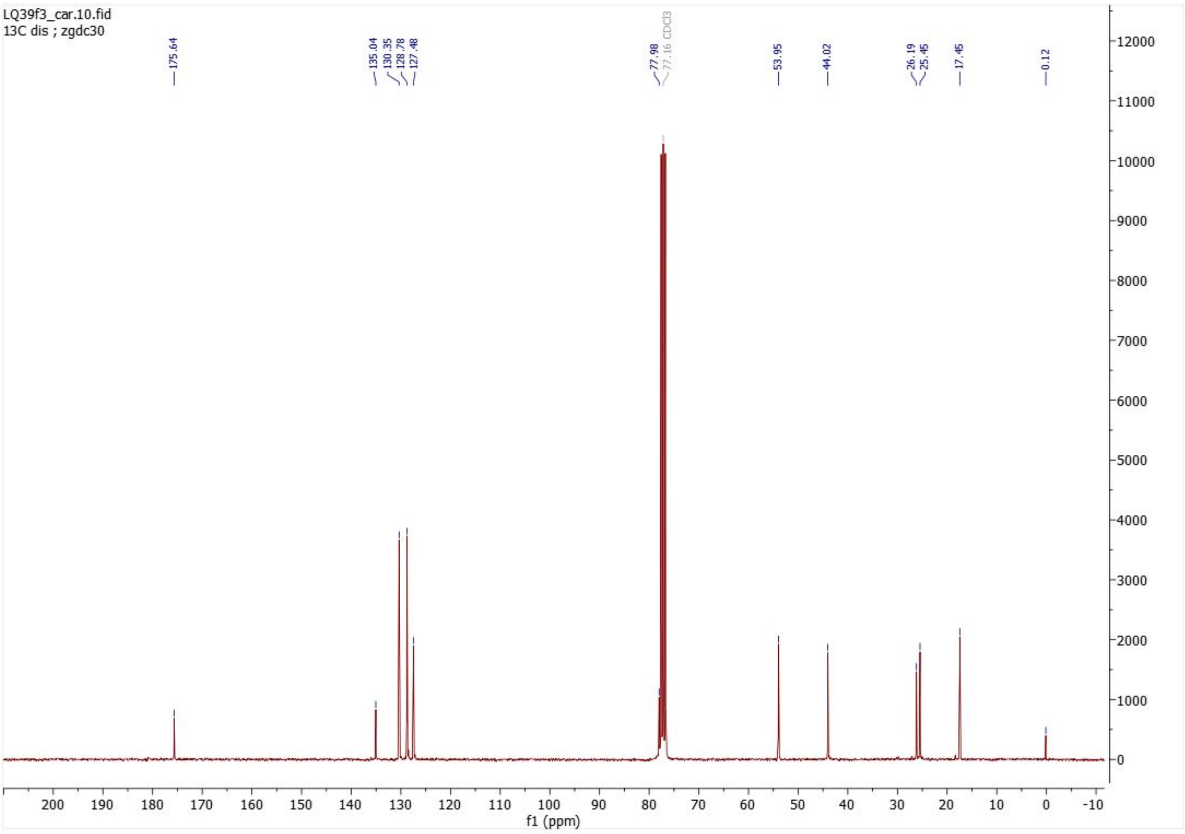

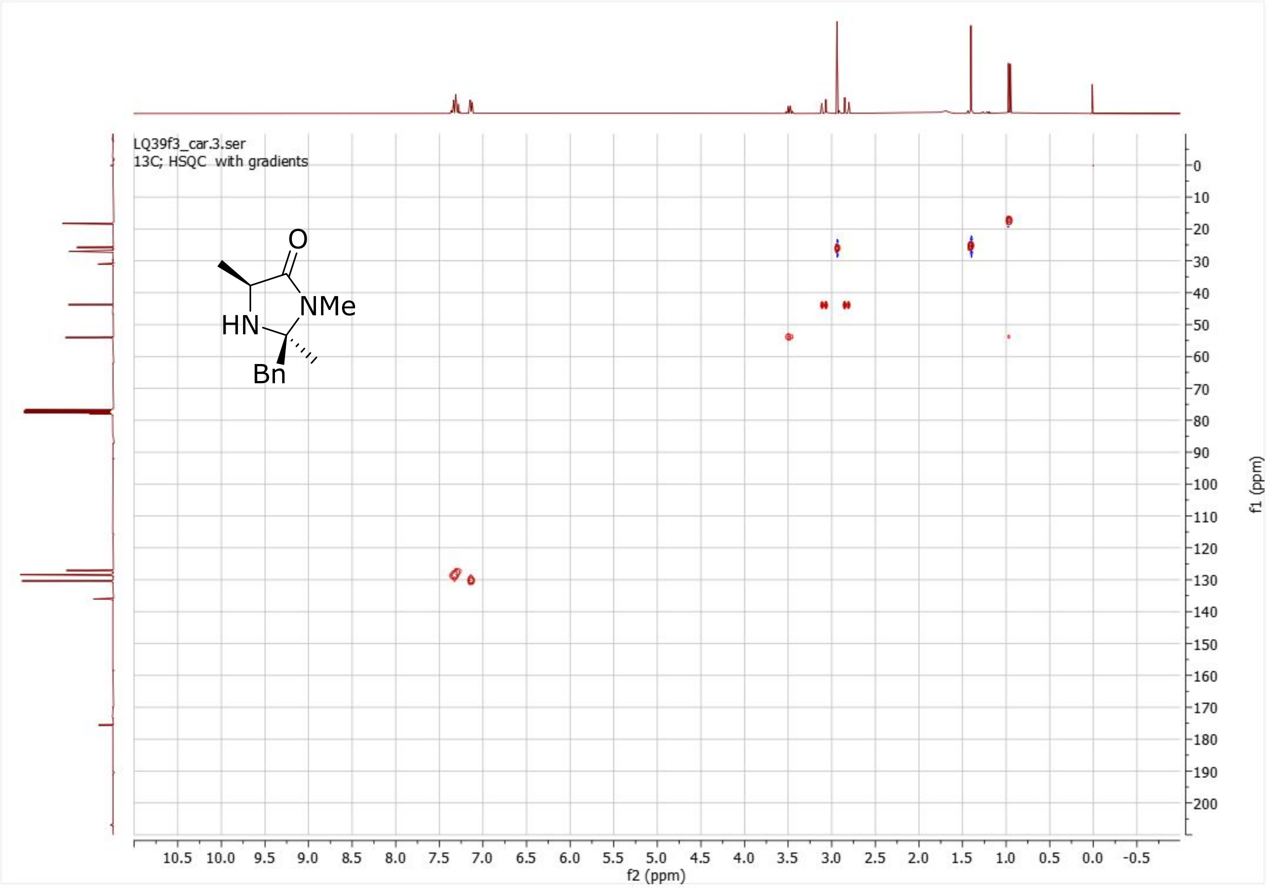

LQ39f3_car.3.ser
13C; HSQC with gradients
O
NMe
HN
Bn
f2 (ppm)
f1 (ppm)

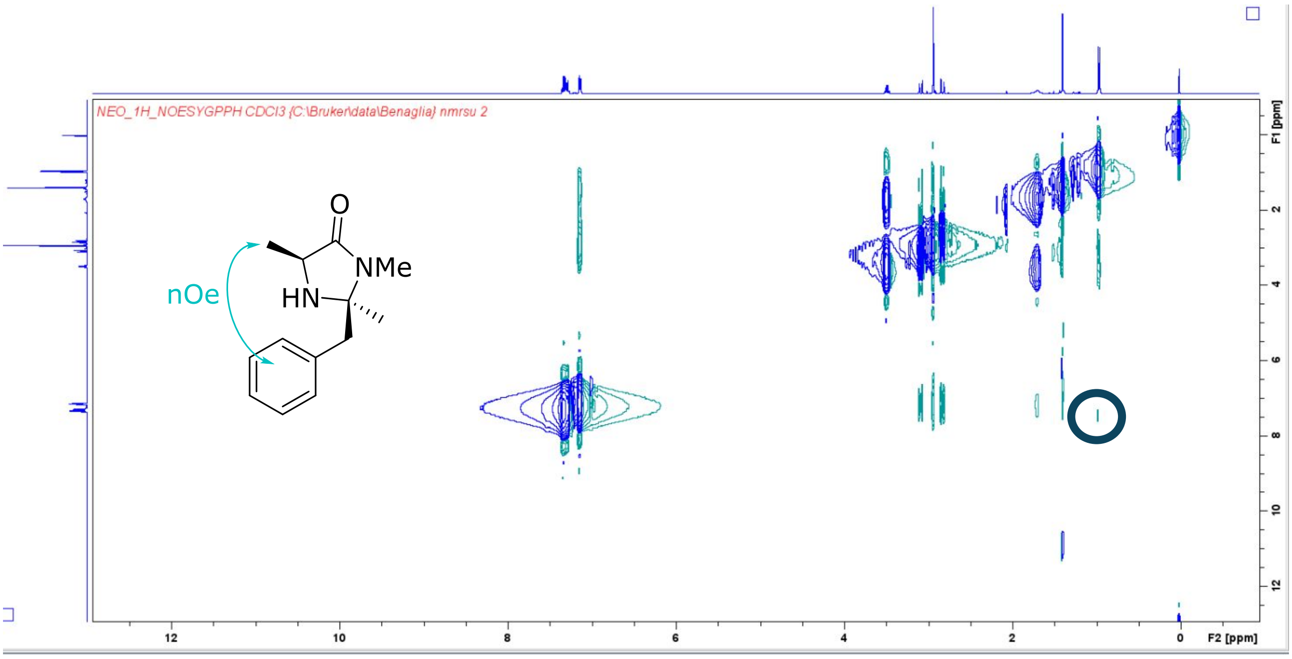

NEO_1H_NOESYGPPH CDCl3 {C:\Bruker\data\Benaglia} nmrsu 2
O
NMe
HN
nOe
F2 [ppm]
F1 [ppm]

(2*R*,5*S*)-2-benzyl-2,3,5-trimethylimidazolin-4-one (Imi-4) ^{1}H NMR (400 MHz, $CDCl_3$); ^{13}C NMR (75 MHz, $CDCl_3$).

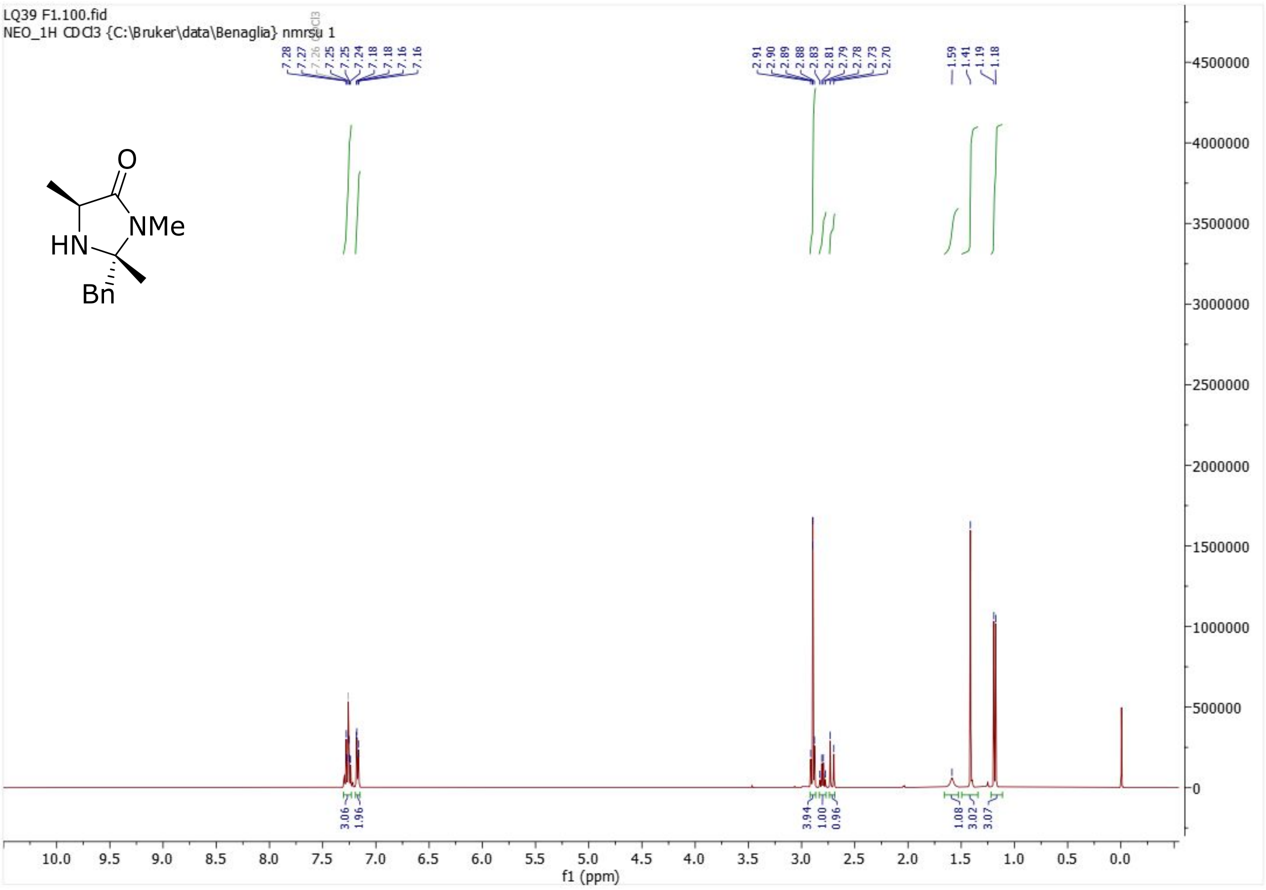

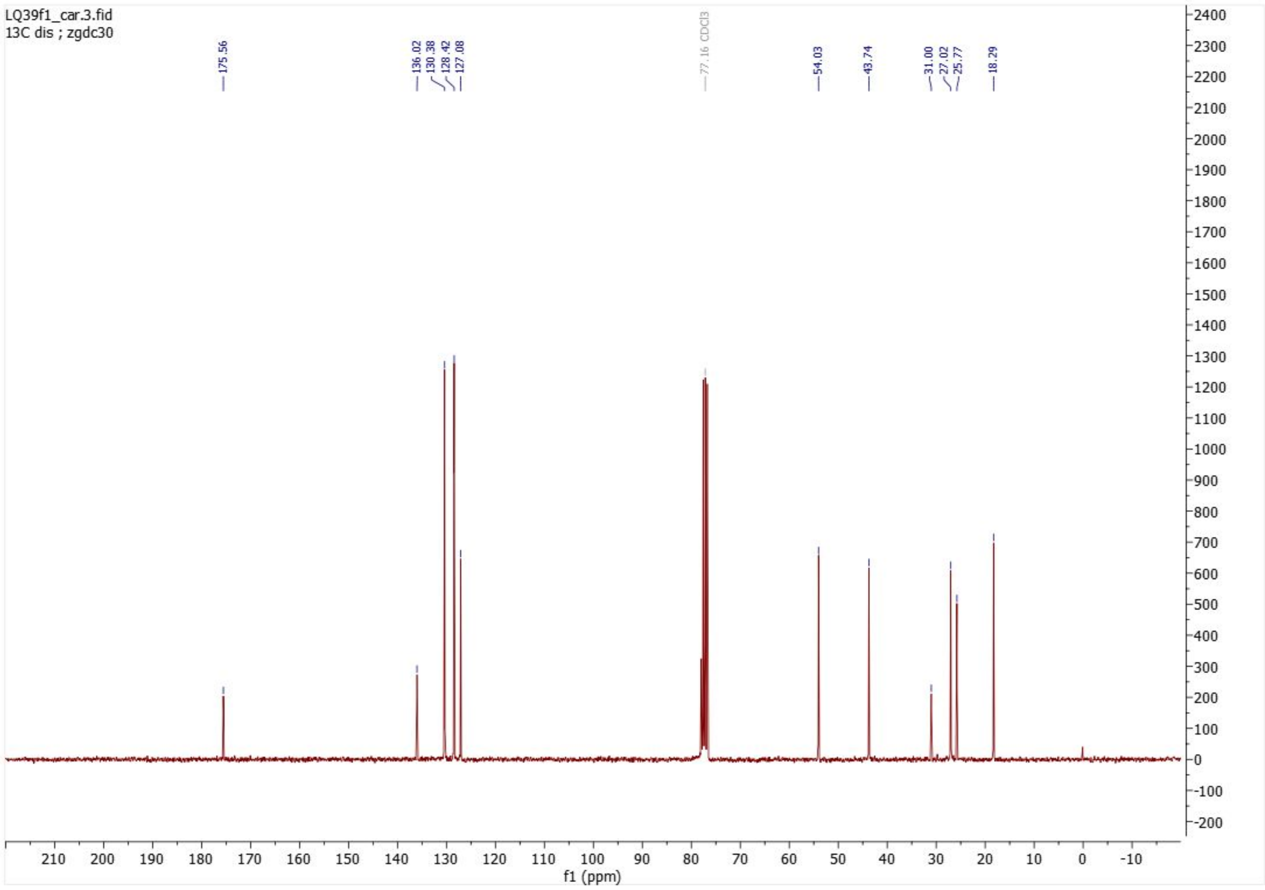

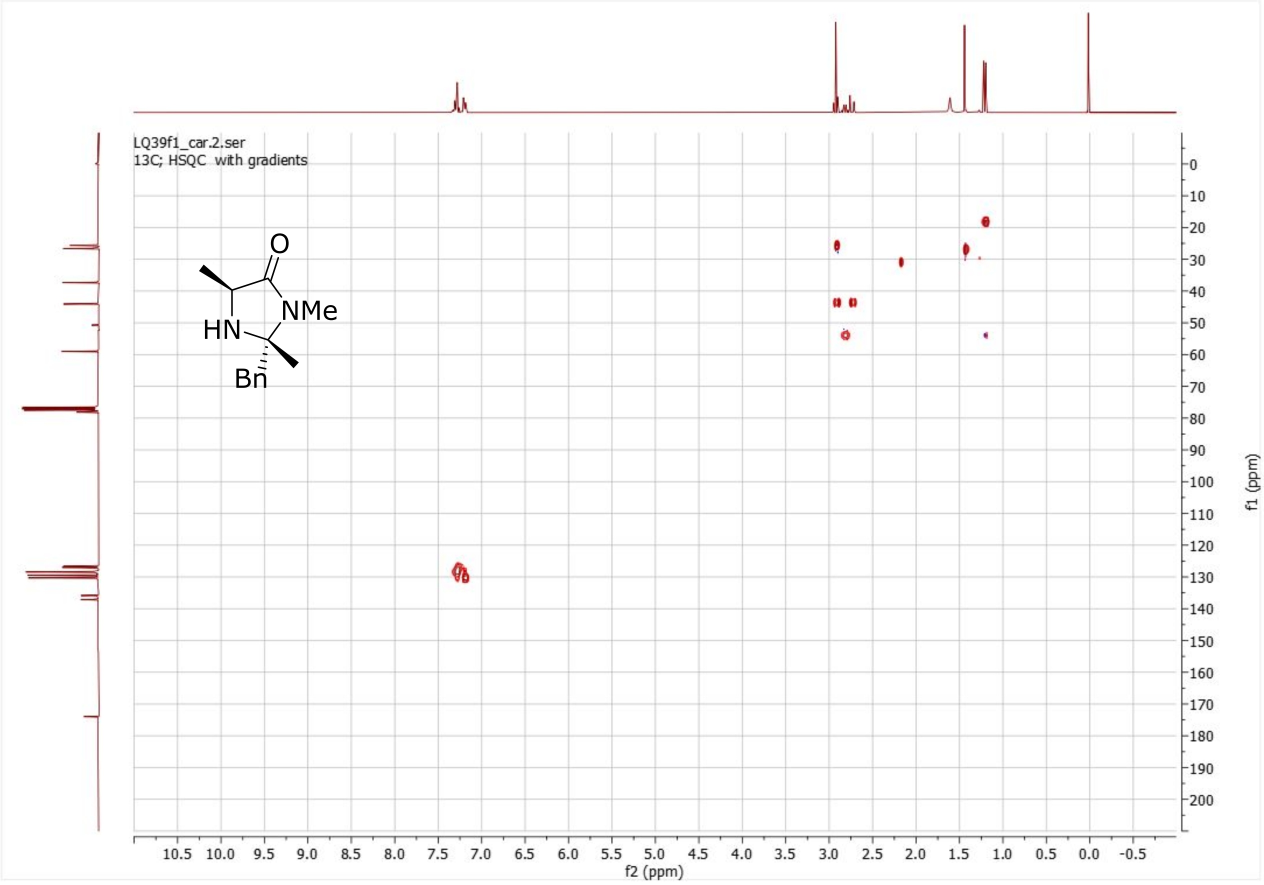
LQ39f1_car.2.ser
13C; HSQC with gradients
O
NMe
HN
Bn
f1 (ppm)
f2 (ppm)

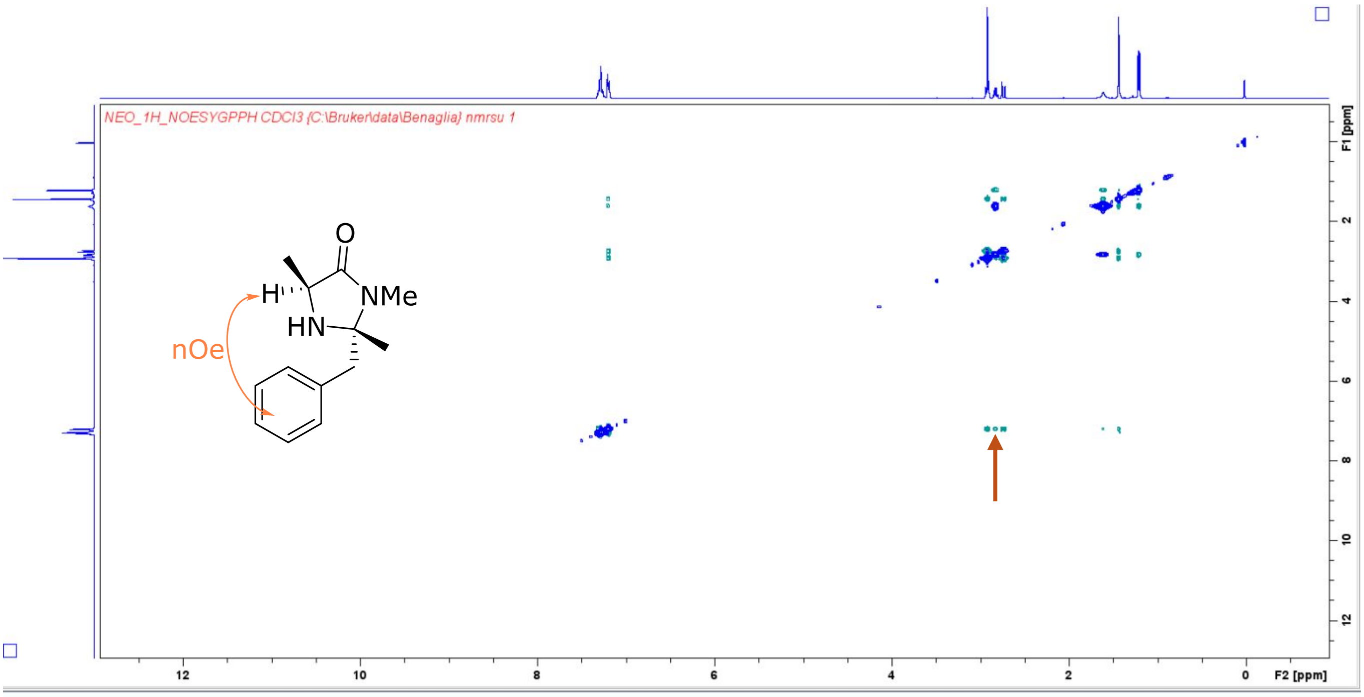
NEO_1H_NOESYGPPH CDCl3 {C:\Bruker\data\Benaglia} nmrsu 1
O
H
NMe
HN
nOe
F1 [ppm]
F2 [ppm]

(2*S*,5*S*)-2-(*tert*-butyl)-2,3,5-trimethylimidazolidin-4-one (Imi-7) ^{1}H NMR (300 MHz, $CDCl_3$); ^{13}C NMR (75 MHz, $CDCl_3$).

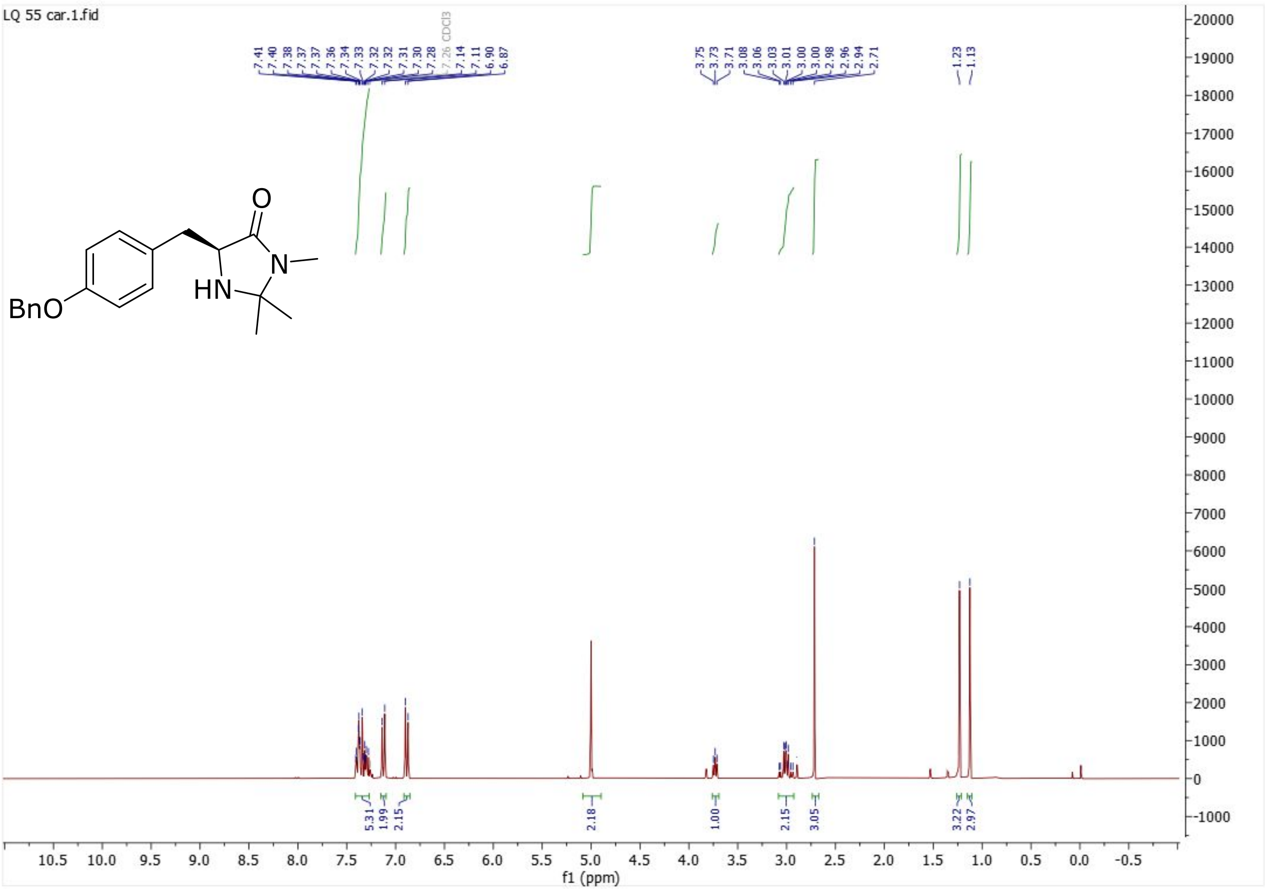

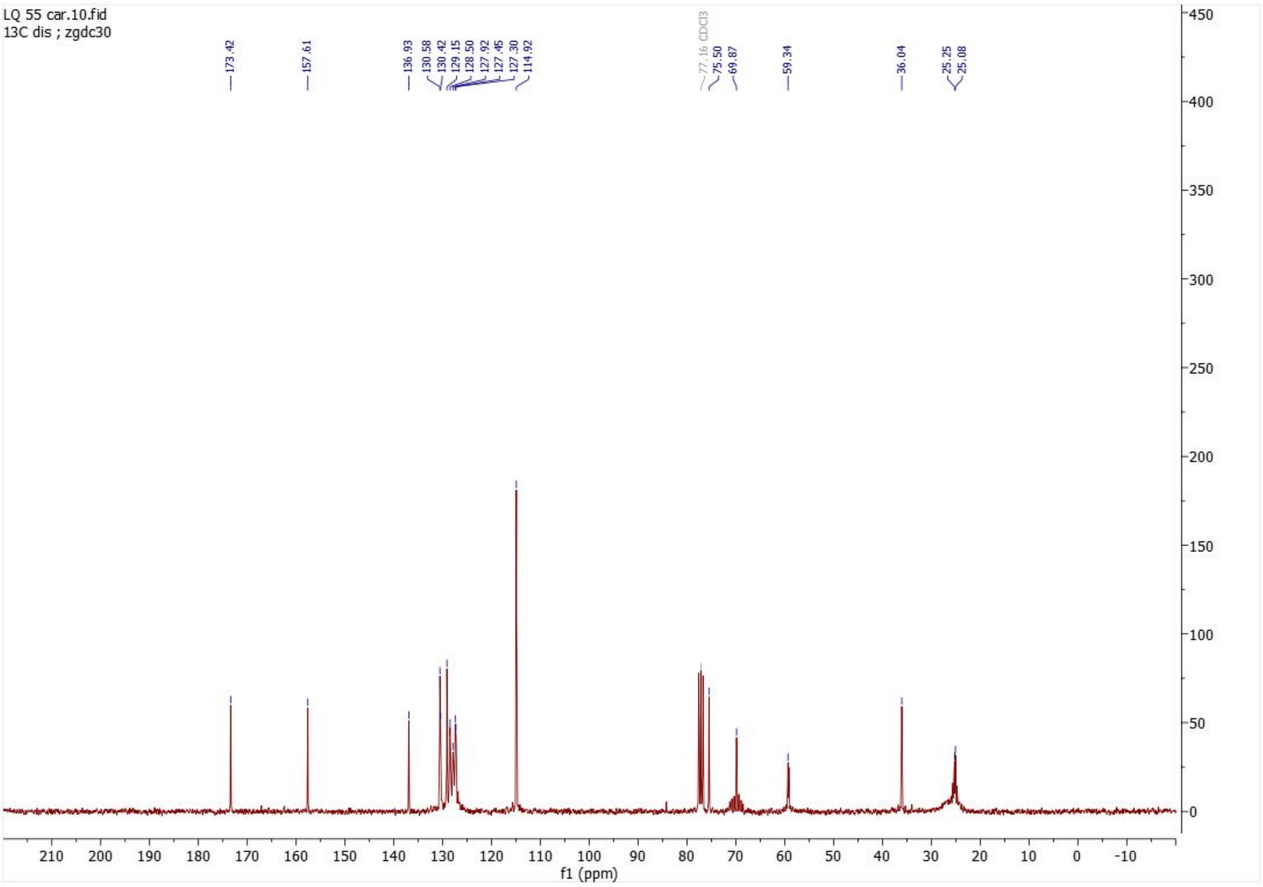

2-(2-Oxo-pyrrolidin-1-yl)-butyraldehyde (3aa) ^{1}H NMR (300 MHz, $CDCl_3$); ^{13}C NMR (75 MHz, $CDCl_3$).

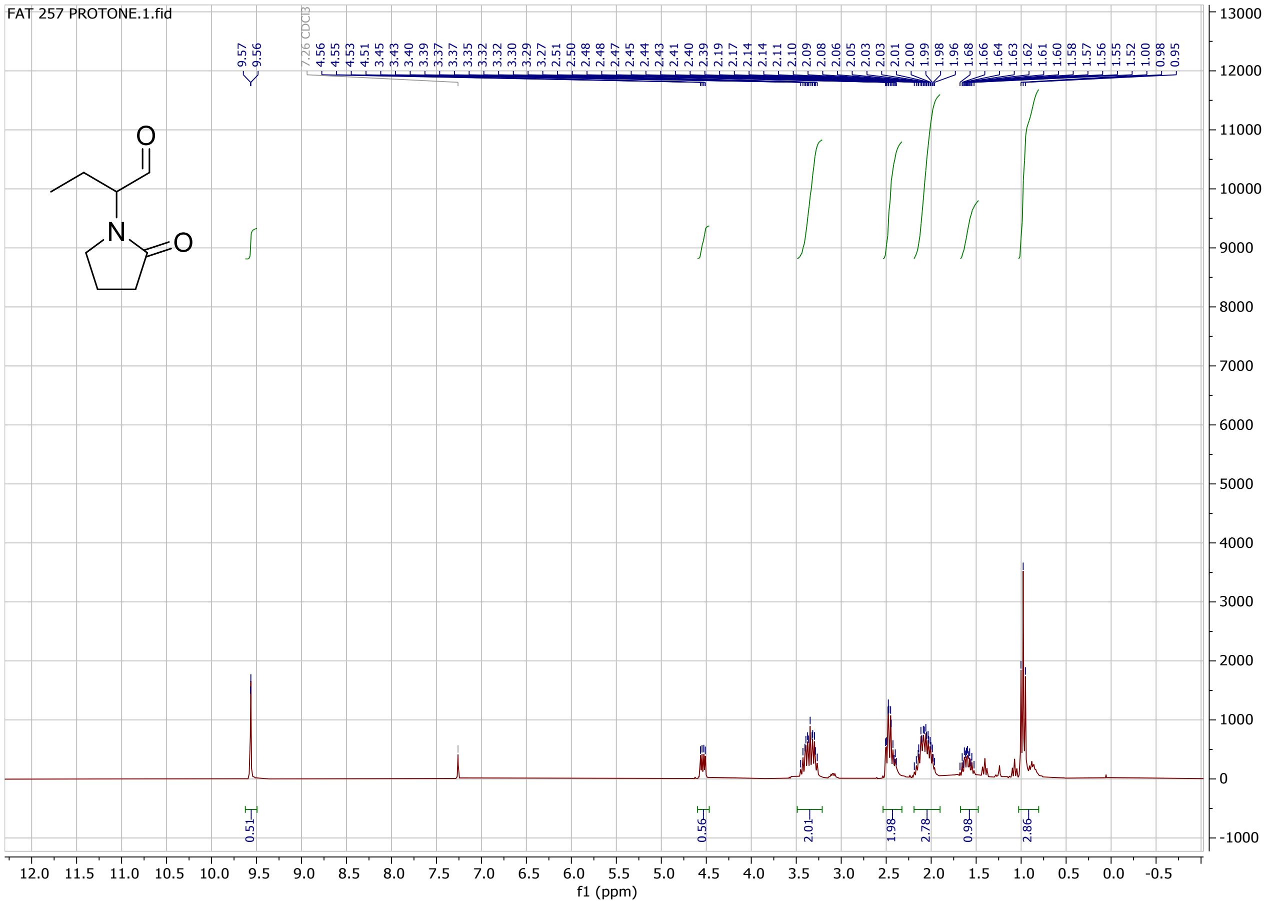

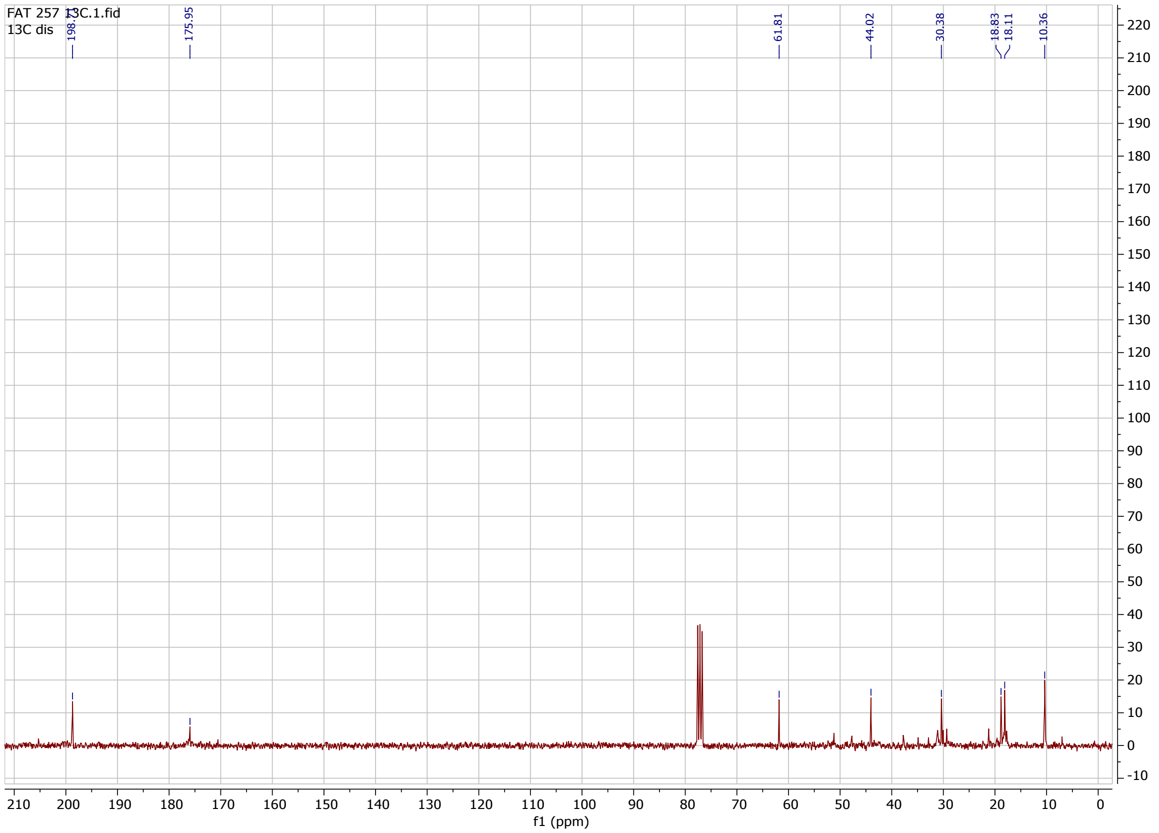

1-(1-oxo-3-phenylpropan-2-yl)pyrrolidin-2-one (3da) ^{1}H NMR (300 MHz, $CDCl_3$); ^{13}C NMR (75 MHz, $CDCl_3$).

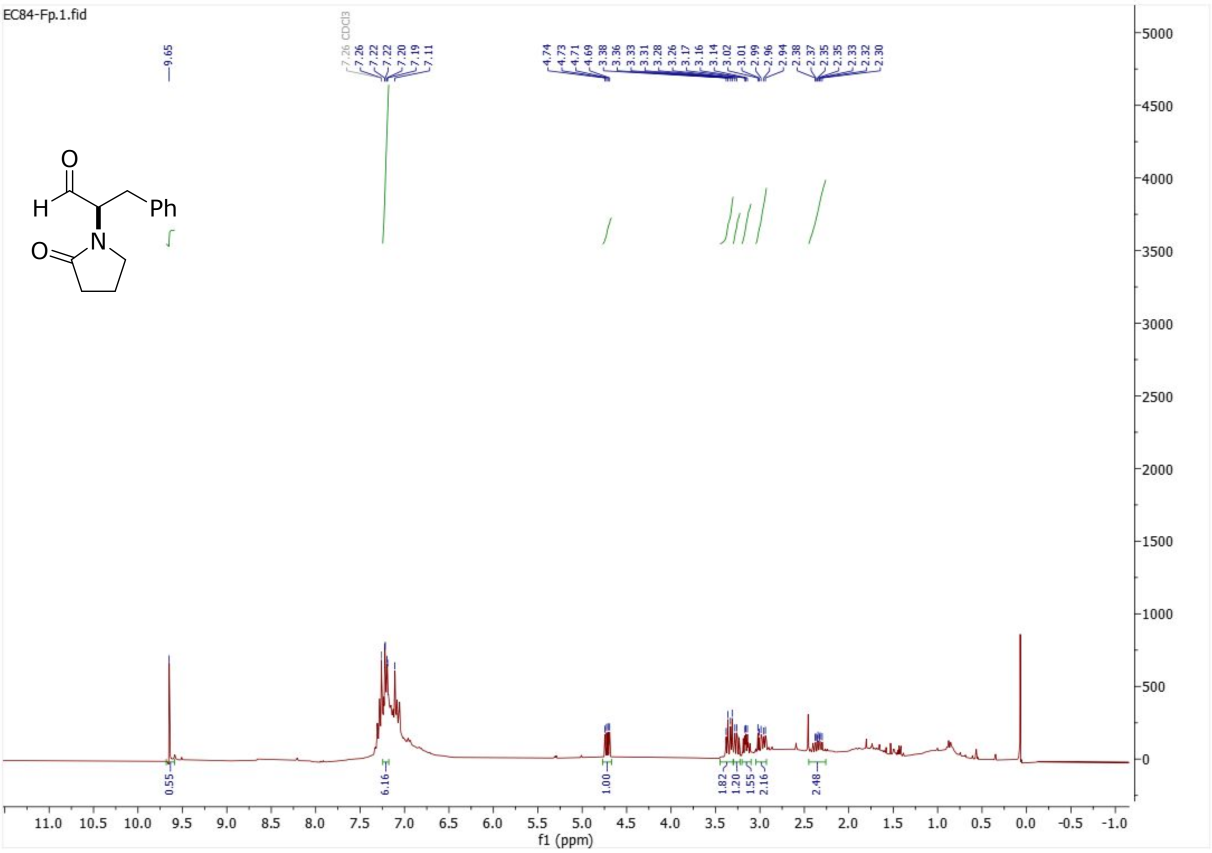

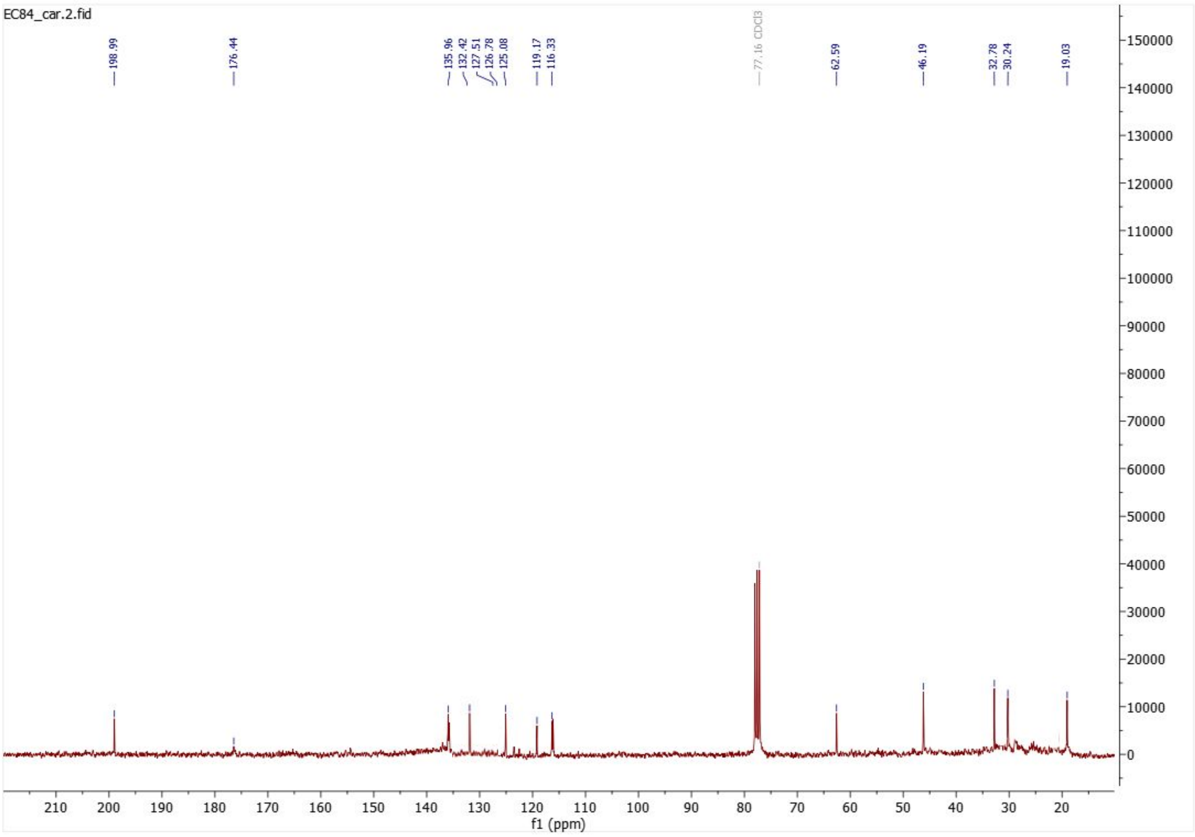

(*S*)-1-(1-hydroxybutan-2-yl)pyrrolidin-2-one (4aa) ^{1}H NMR (300 MHz, $CDCl_3$); ^{13}C NMR (75 MHz, $CDCl_3$).

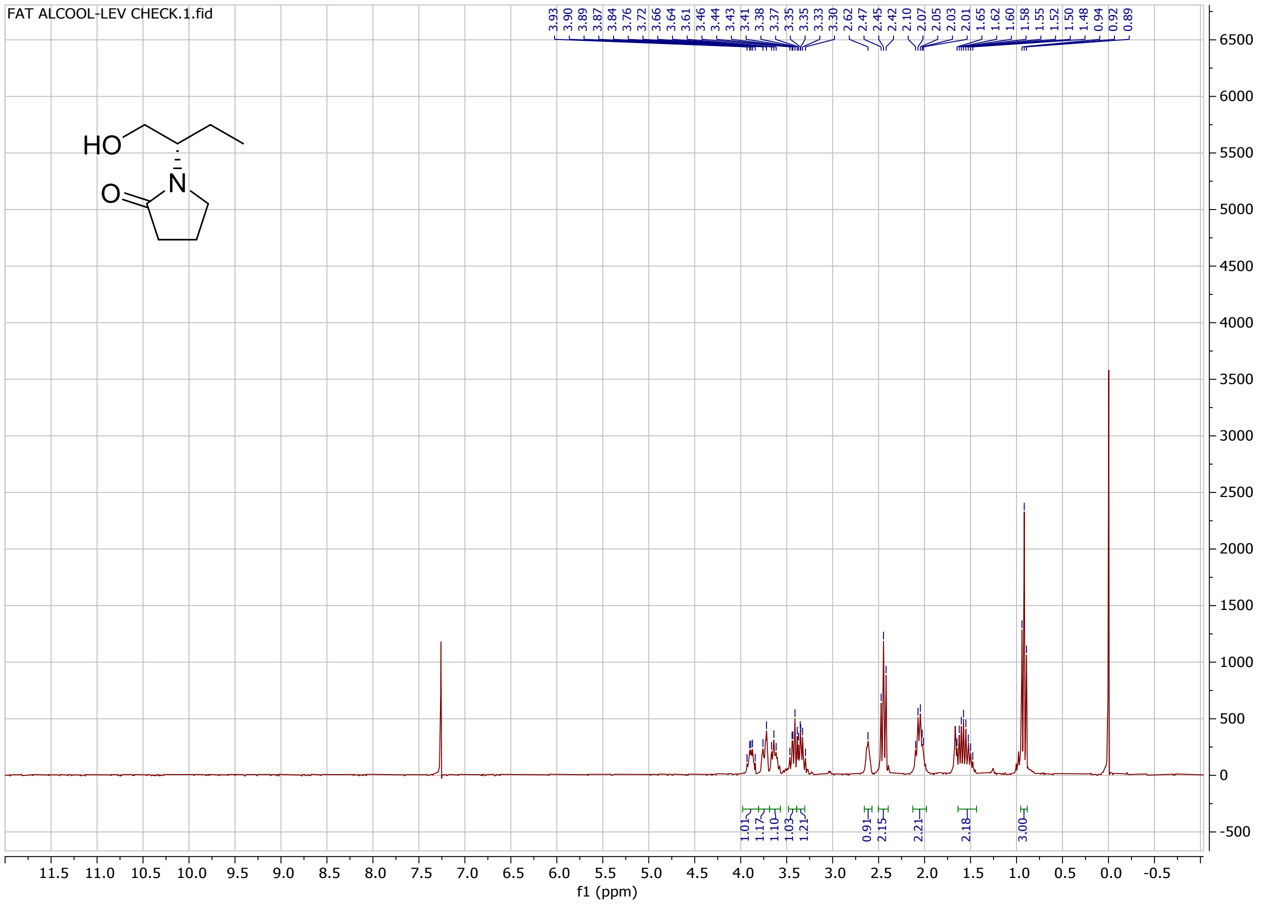

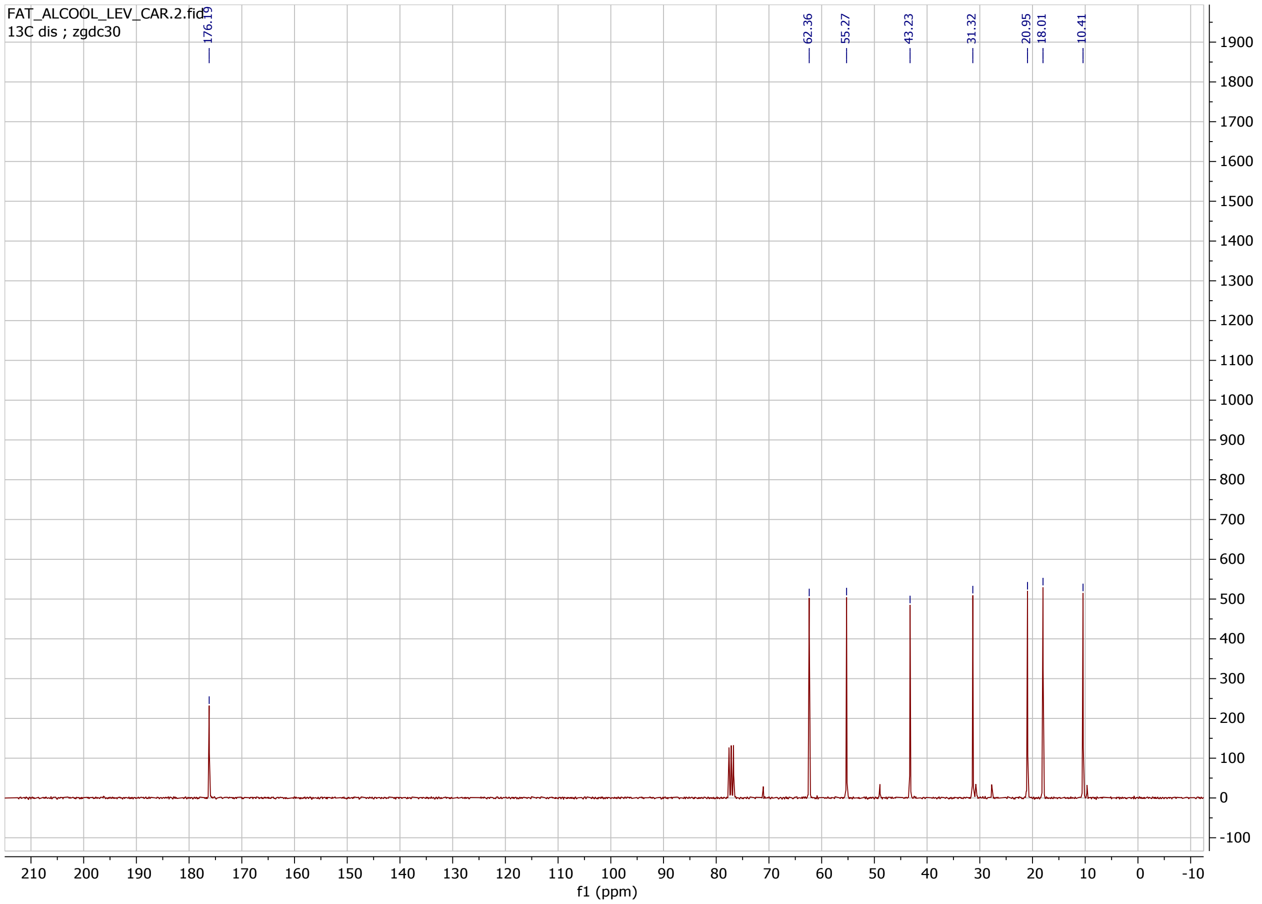

(S)-2-(2-oxopyrrolidin-1-yl)butyl 2-naphthoate ^{1}H NMR (300 MHz, $CDCl_3$); ^{13}C NMR (75 MHz, $CDCl_3$).

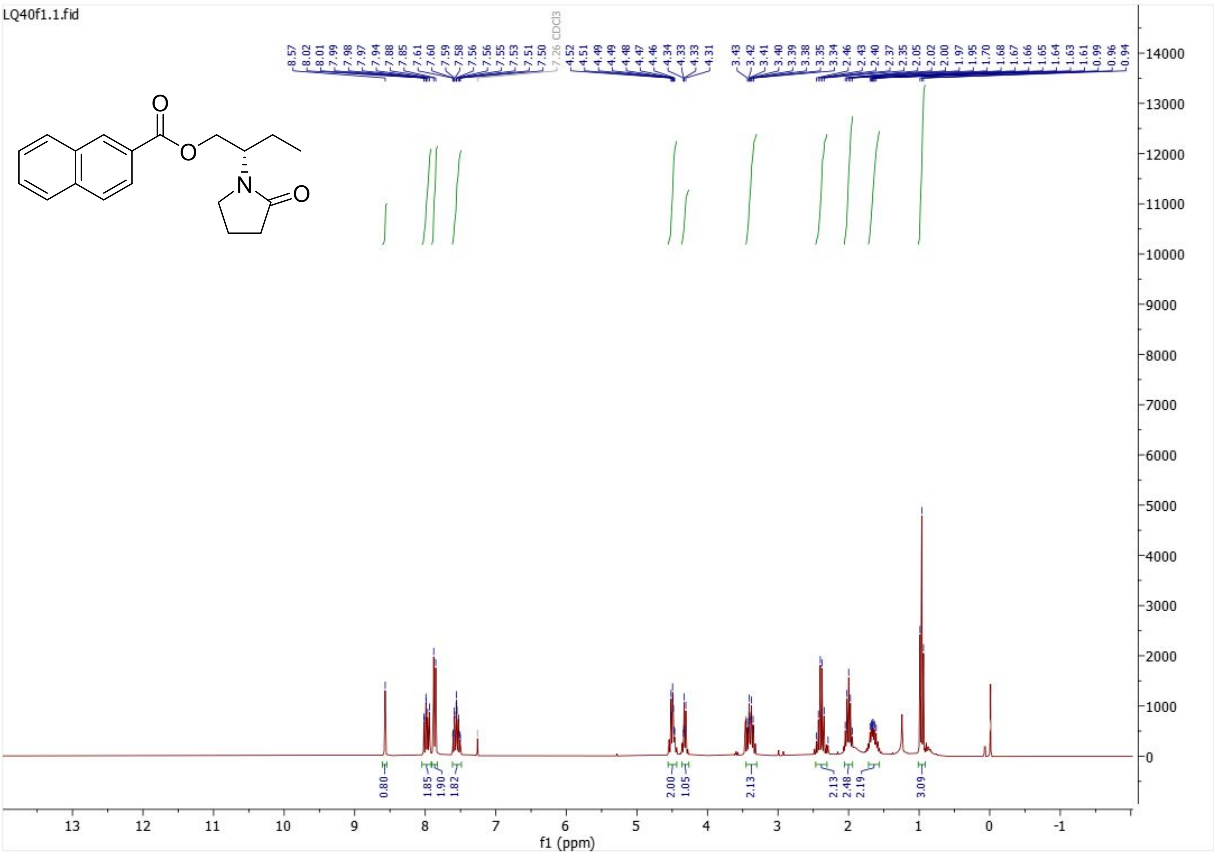
LQ40f1.1.fid
8.57 8.02 8.01 7.99 7.98 7.97 7.94 7.88 7.85 7.61 7.60 7.59 7.58 7.56 7.56 7.55 7.53 7.51 7.50
7.26 CDCl3
4.52 4.51 4.49 4.48 4.47 4.46 4.34 4.33 4.33 4.31
3.43 3.42 3.41 3.40 3.39 3.38 3.35 3.34 2.46 2.43 2.40 2.37 2.35 2.05 2.02 2.00 1.97 1.95 1.70 1.68 1.67 1.66 1.65 1.64 1.63 1.61 0.99 0.96 0.94
0.80 1.85 1.90 1.82 2.00 1.05 2.13 2.13 2.48 2.19 3.09
f1 (ppm)

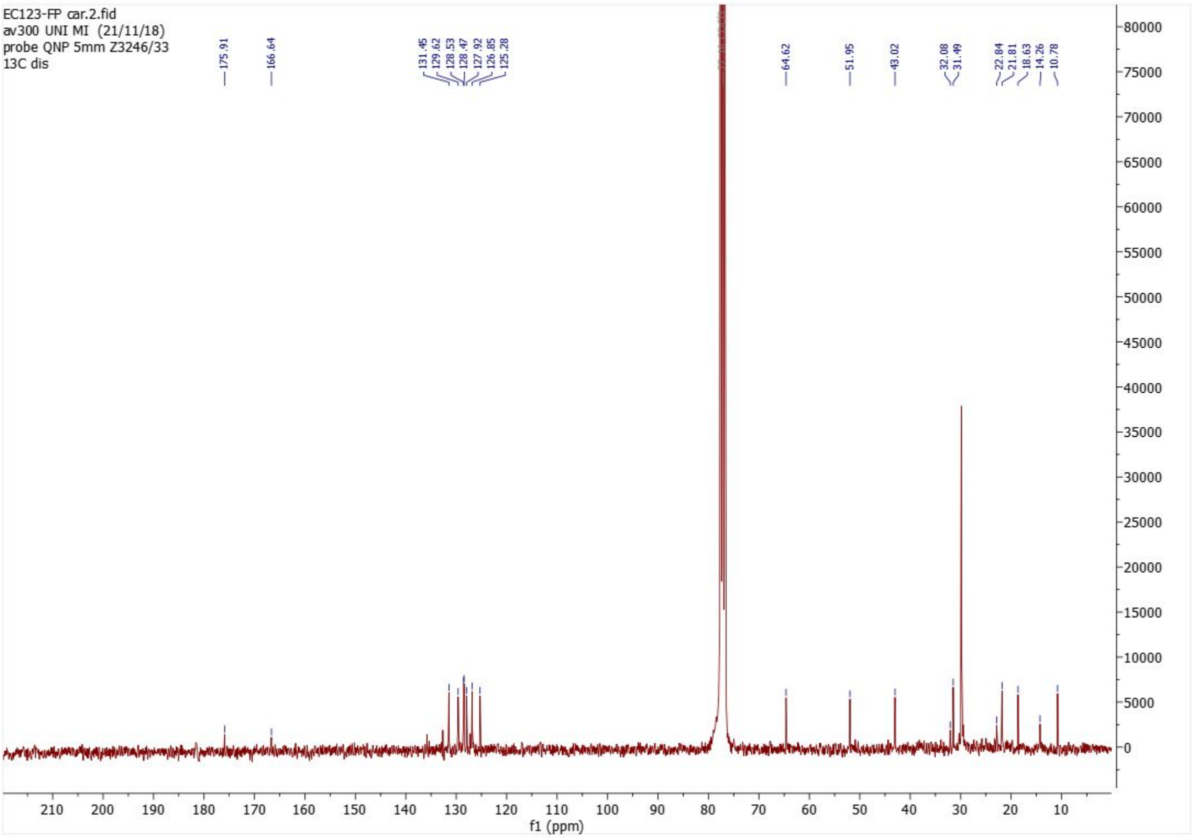
EC123-FP car.2.fid
av300 UNI MI (21/11/18)
probe QNP 5mm Z3246/33
13C dis
175.91 166.64
131.45 129.62 128.53 128.47 127.92 126.85 125.28
64.62 51.95 43.02 32.08 31.49 22.84 21.81 18.63 14.26 10.78
f1 (ppm)

(*S*)-1-(1-hydroxy-3-methylbutan-2-yl)pyrrolidin-2-one (4ba) ^{1}H NMR (300 MHz, $CDCl_3$); ^{13}C NMR (75 MHz, $CDCl_3$).

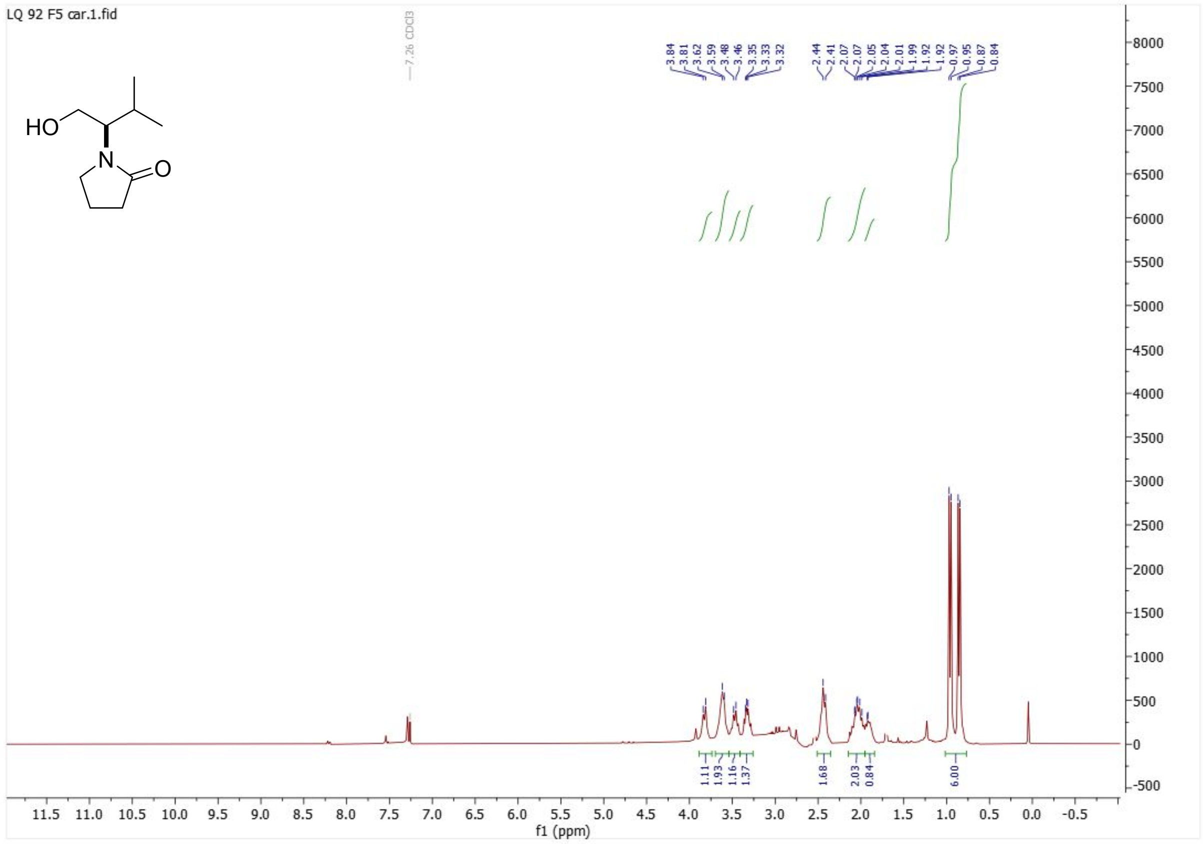

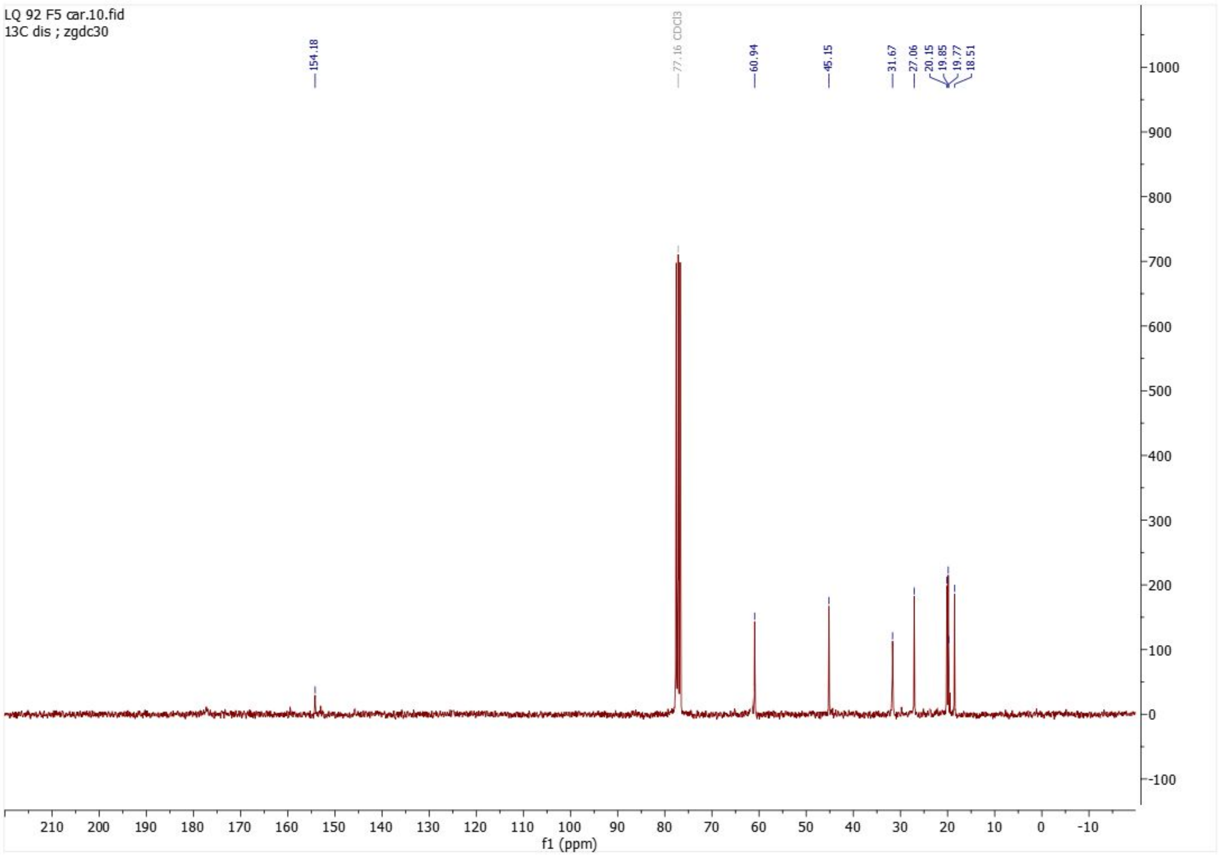

(*S*)-3-methyl-2-(2-oxopyrrolidin-1-yl)butyl 2-naphthoate ^{1}H NMR (300 MHz, $CDCl_3$); ^{13}C NMR (75 MHz, $CDCl_3$).

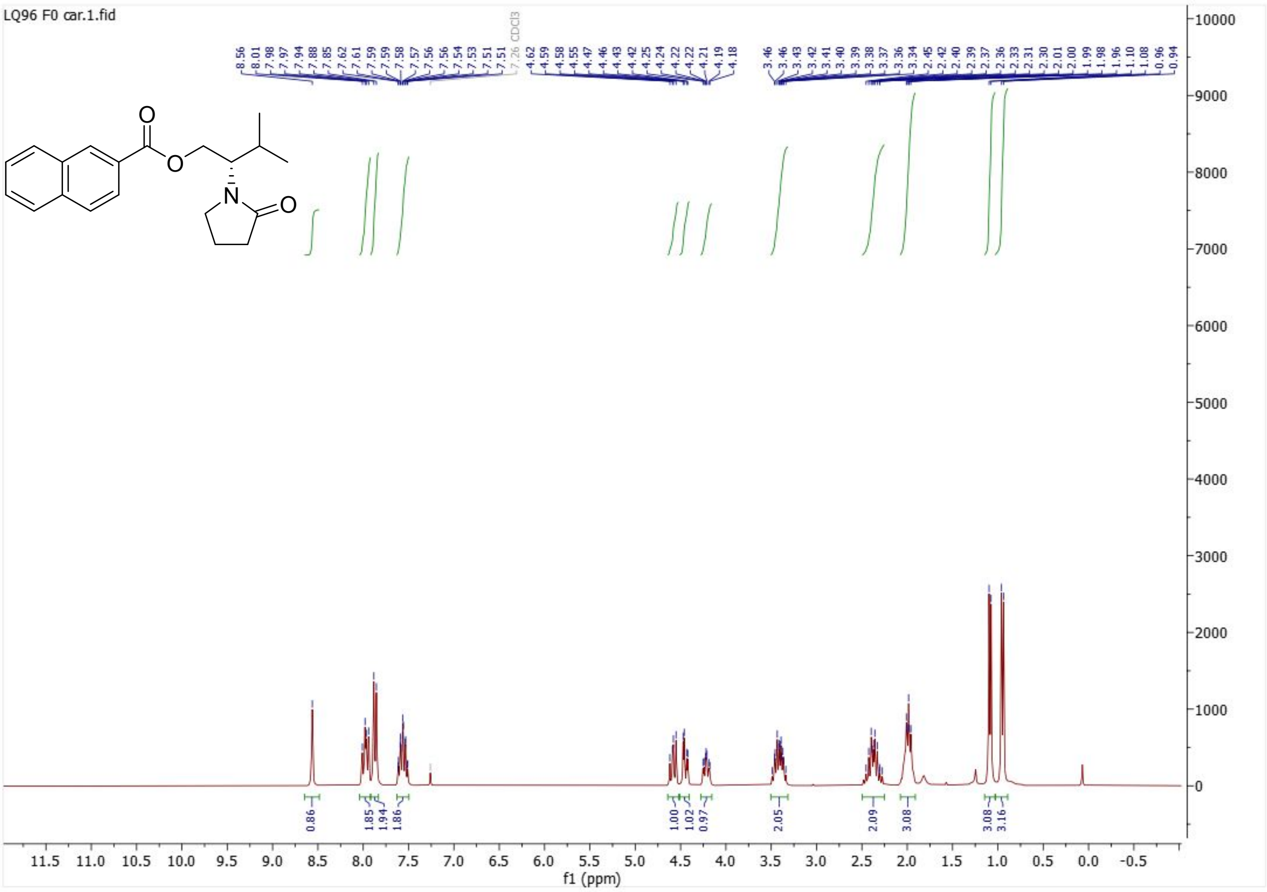

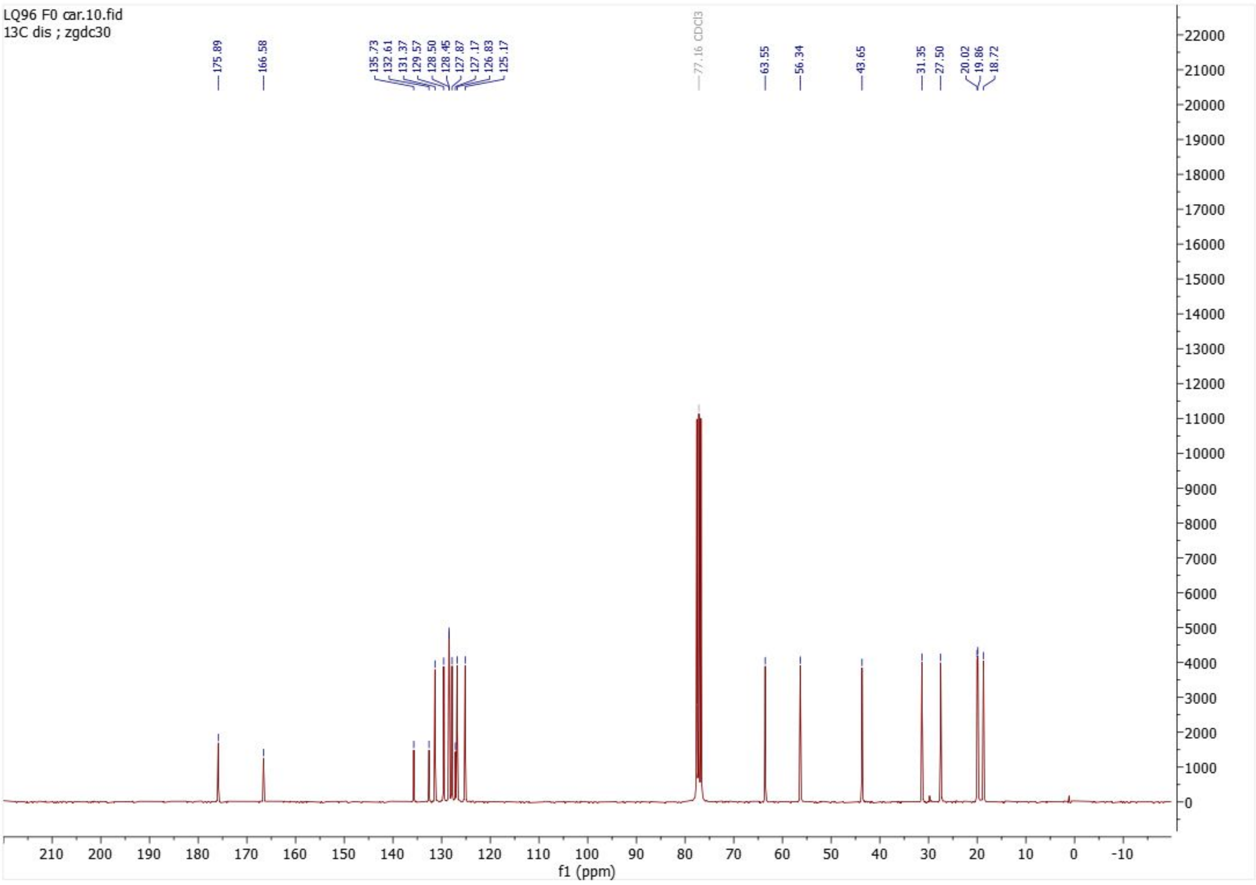

(*S*)-1-(1-(3,4-dimethoxyphenyl)-3-hydroxypropan-2-yl)pyrrolidin-2-one (4ca) ^{1}H NMR (400 MHz, $CDCl_3$); ^{13}C NMR (101 MHz, $CDCl_3$).

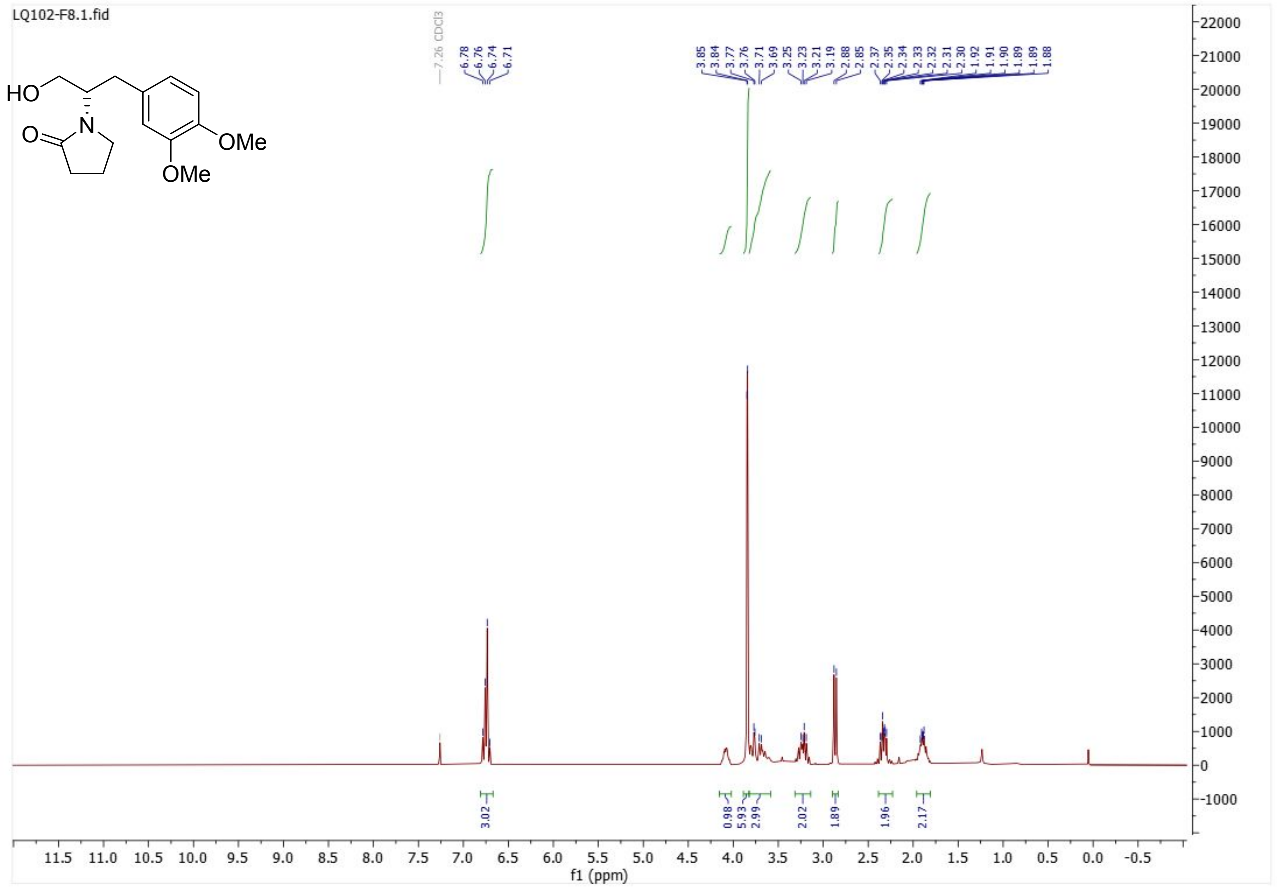

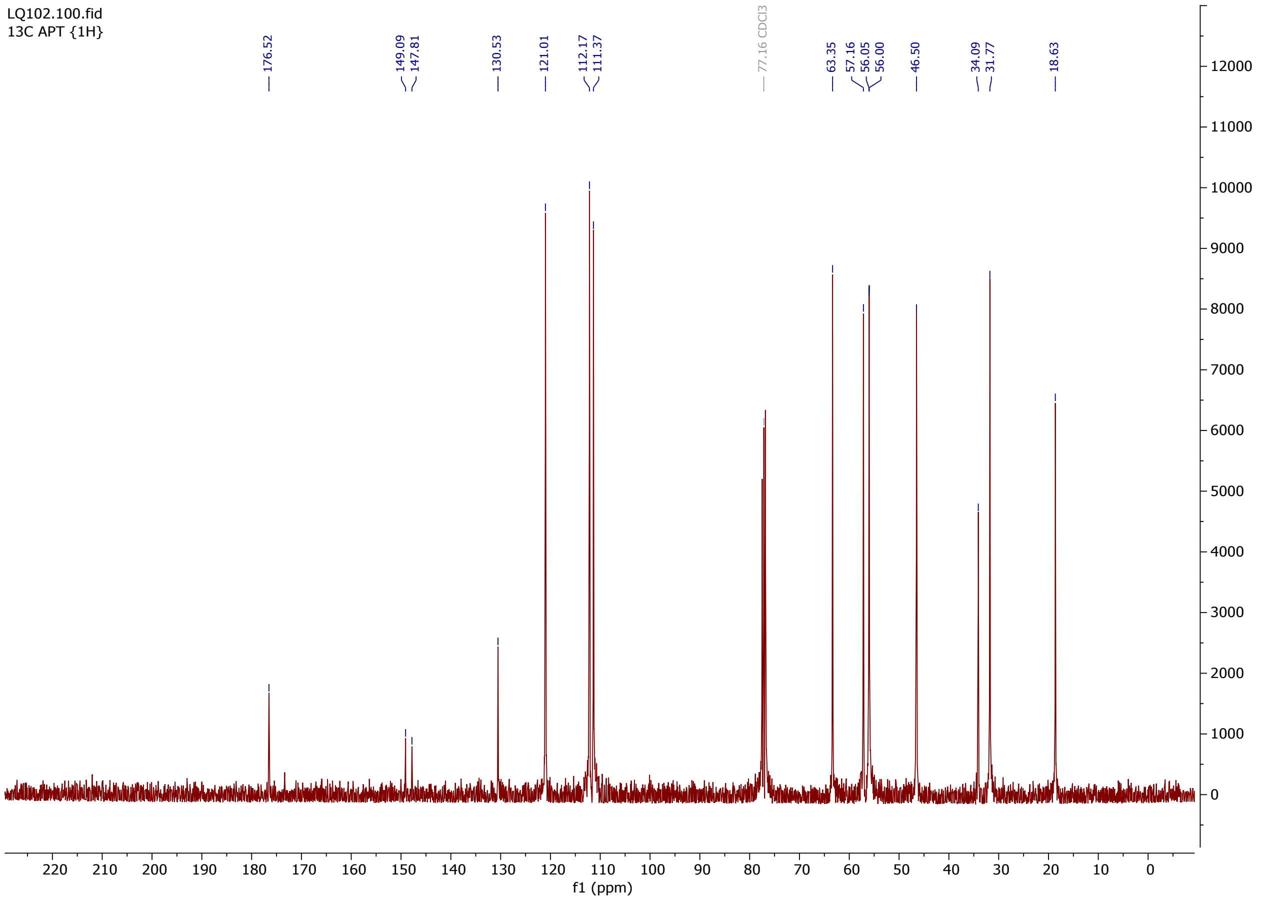

(*S*)-3-(3,4-dimethoxyphenyl)-2-(2-oxopyrrolidin-1-yl)propyl 1 naphthoate ^{1}H NMR (300 MHz, $CDCl_3$); ^{13}C NMR (75 MHz, $CDCl_3$).

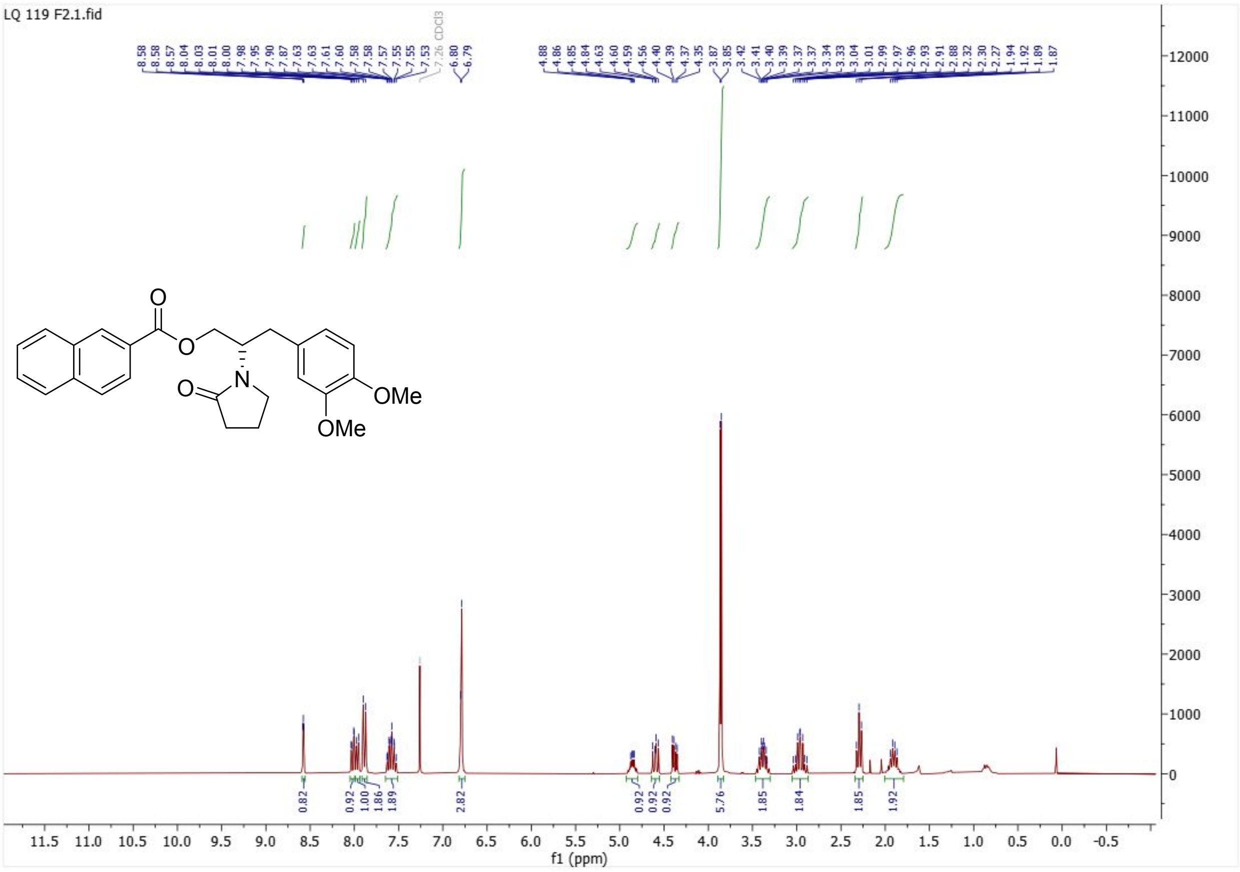

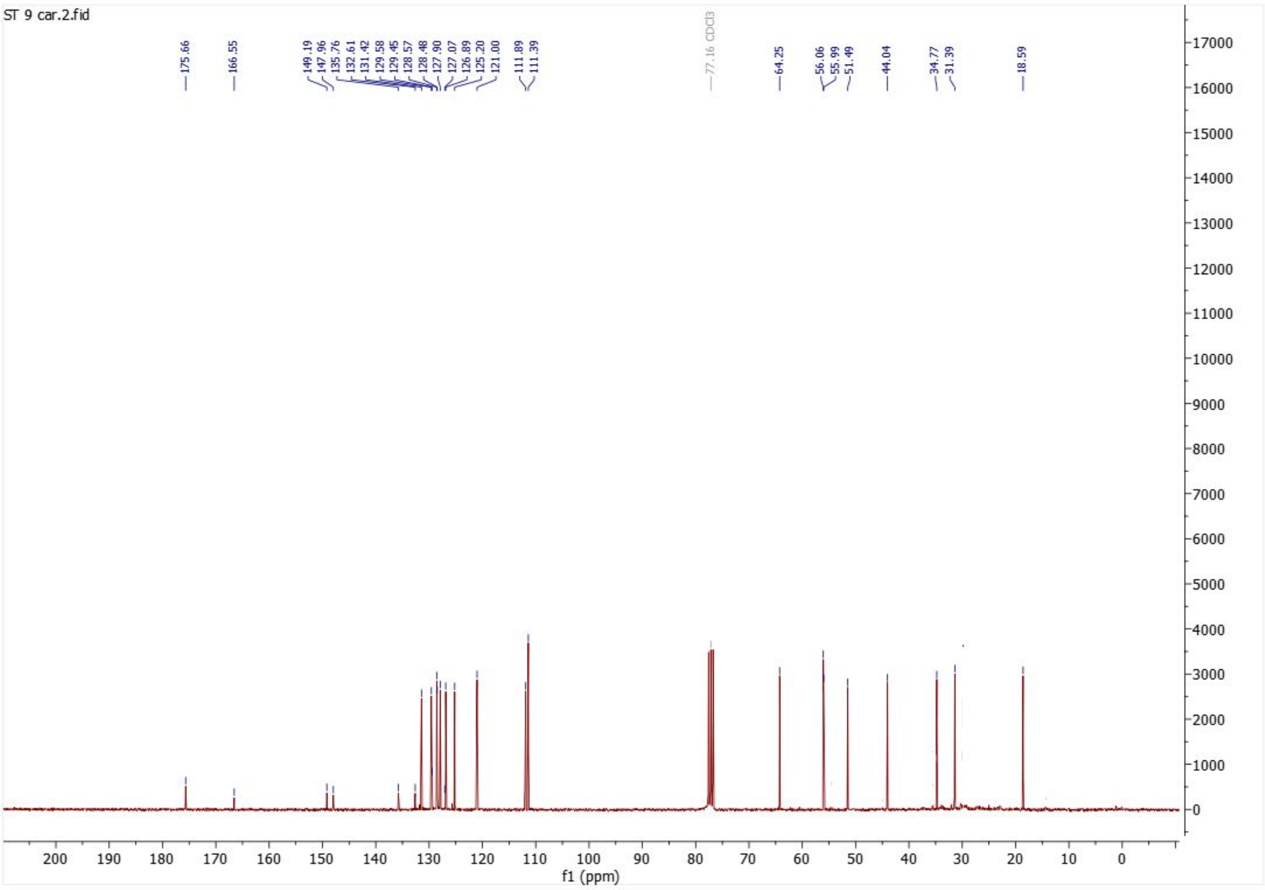

(*S*)-1-(1-hydroxyundec-10-en-2-yl)pyrrolidin-2-one (4ea) ^{1}H NMR (400 MHz, $CDCl_3$); ^{13}C NMR (101 MHz, $CDCl_3$).

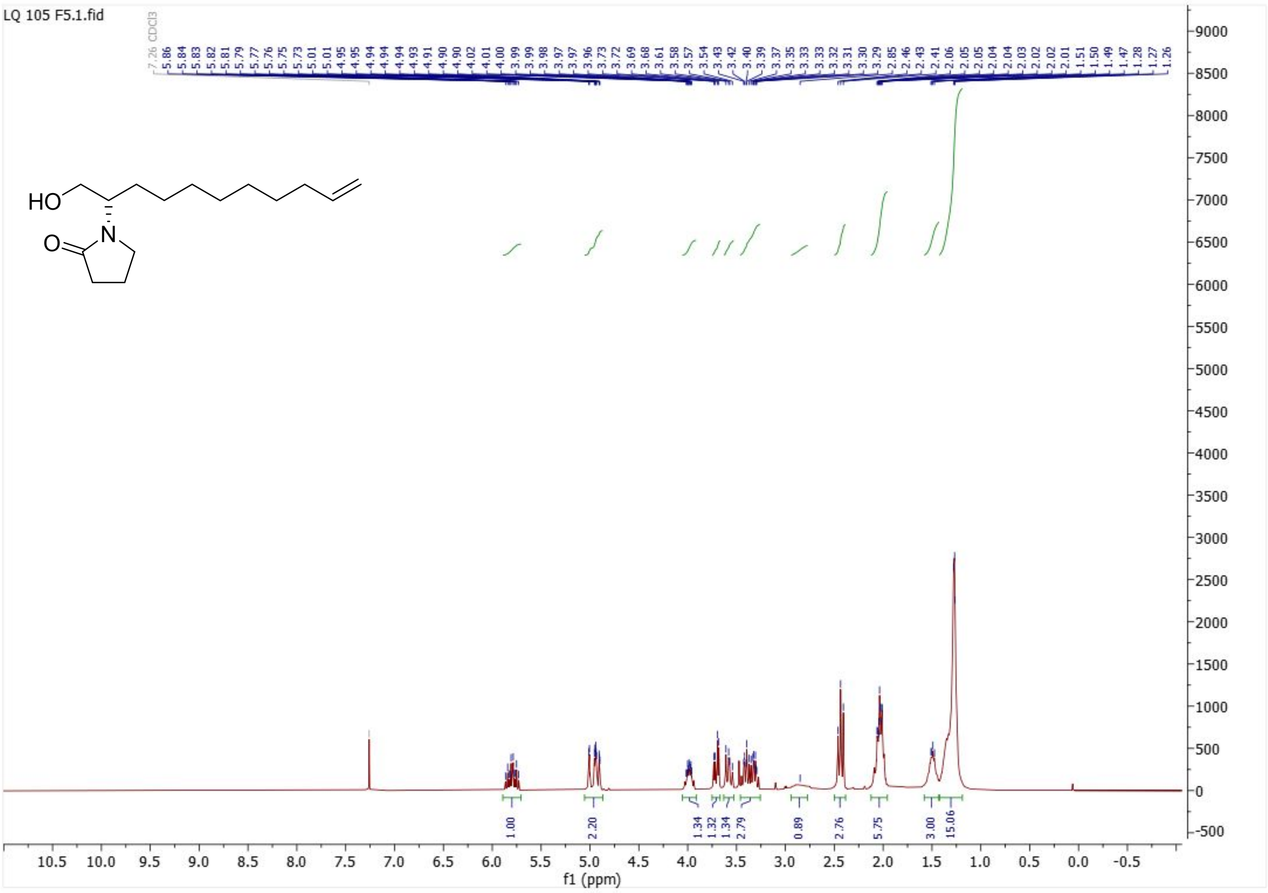

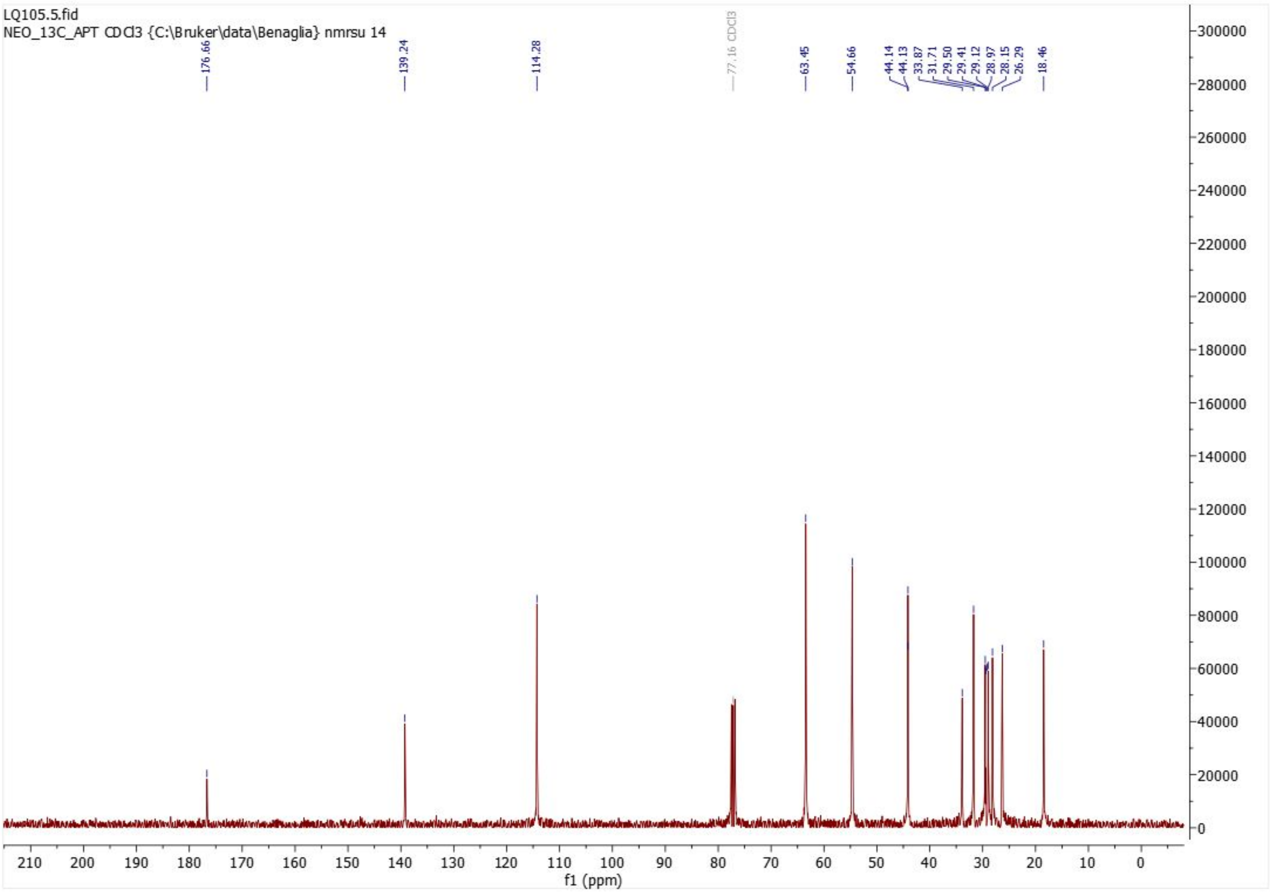

(*S*)-2-(2-oxopyrrolidin-1-yl)undec-10-en-1-yl 2-naphthoate ^{1}H NMR (300 MHz, $CDCl_3$); ^{13}C NMR (75 MHz, $CDCl_3$).

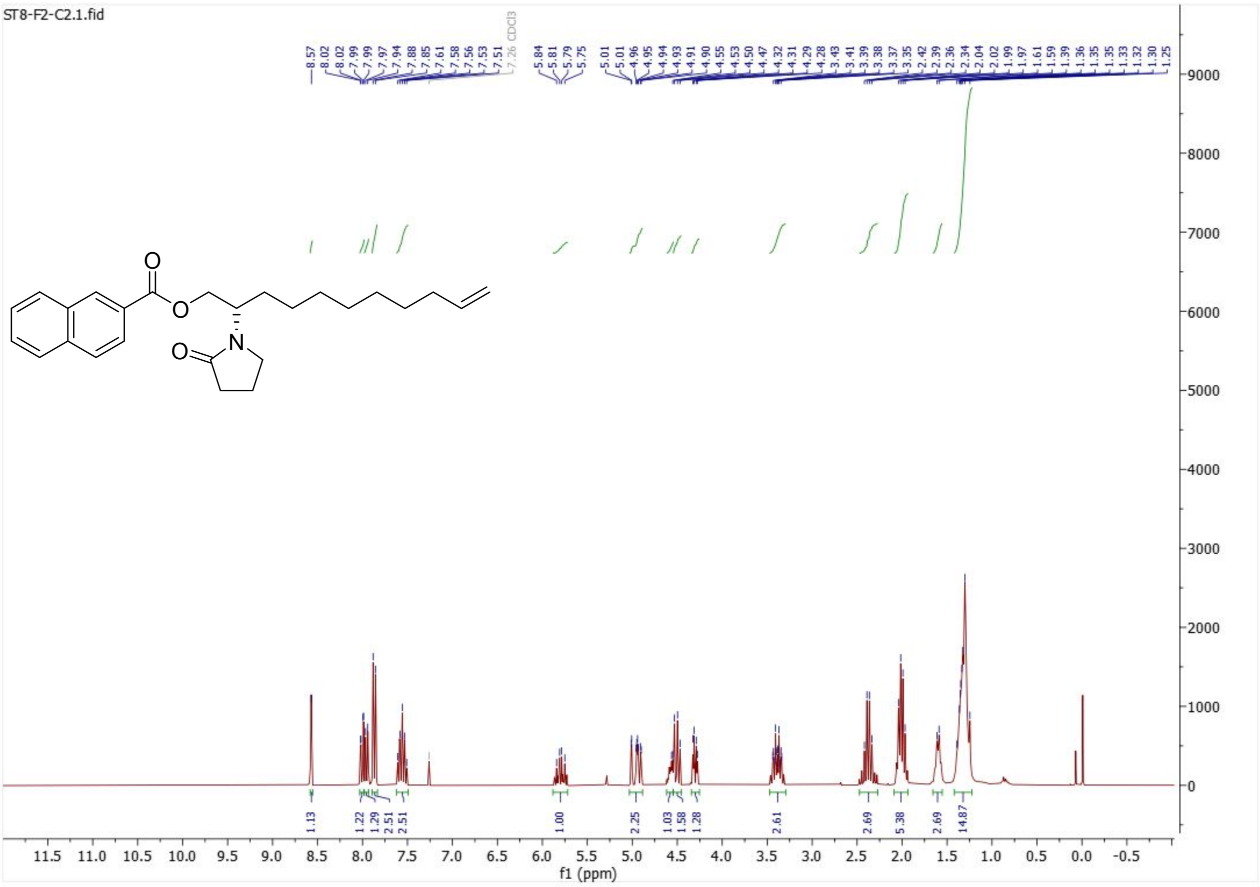

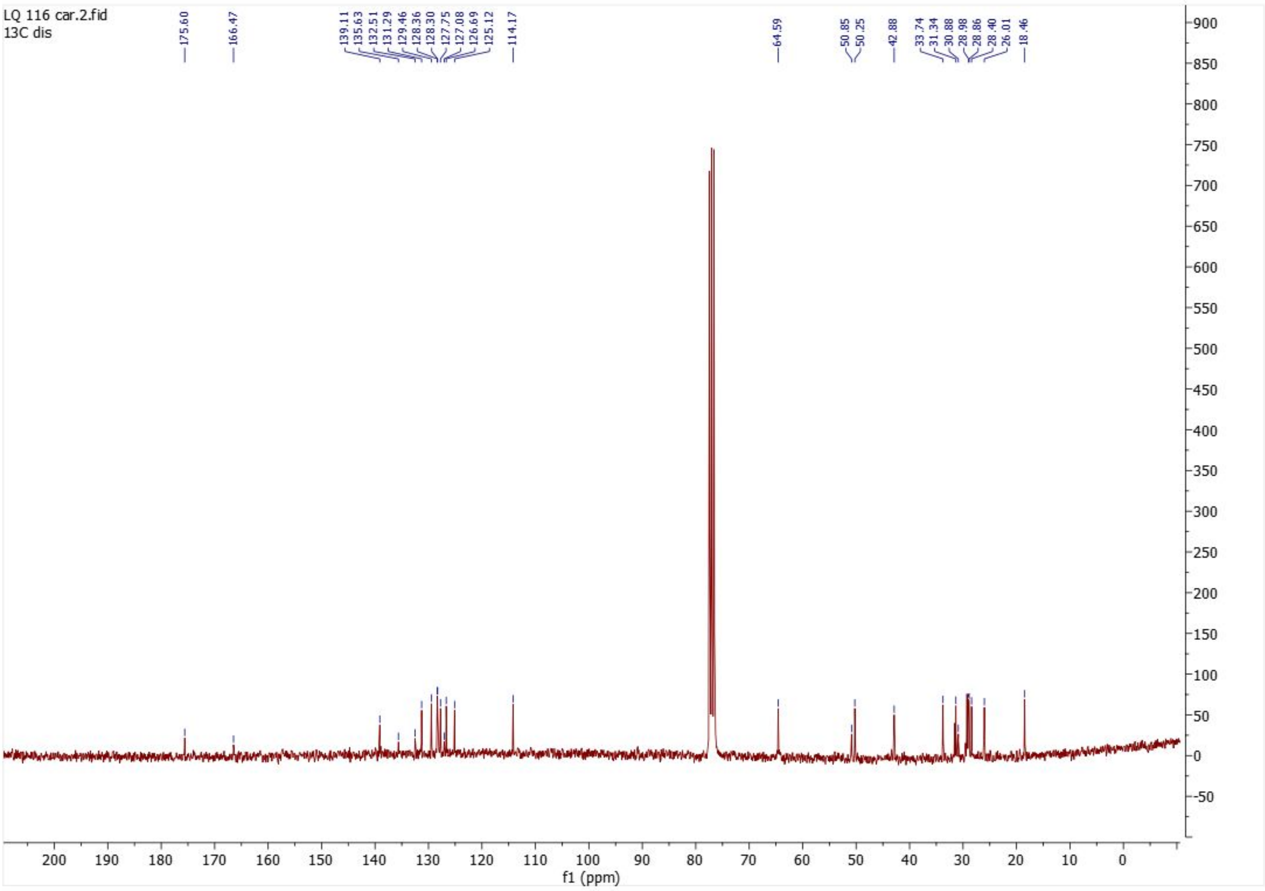

(*S*)-4-(benzyloxy)-1-hydroxybutan-2-yl)pyrrolidine-2-one (4fa) ^{1}H NMR (300 MHz, $CDCl_3$); ^{13}C NMR (75 MHz, $CDCl_3$).

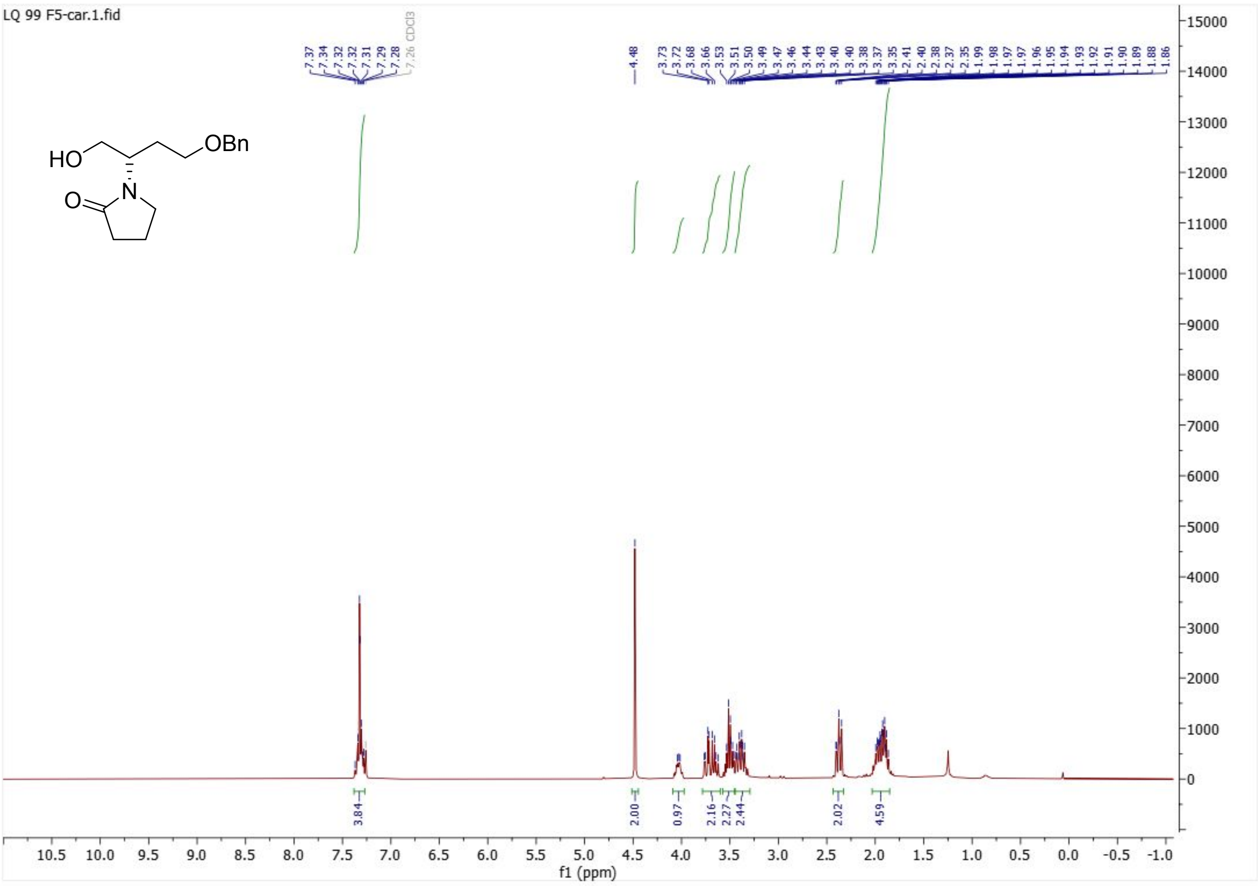

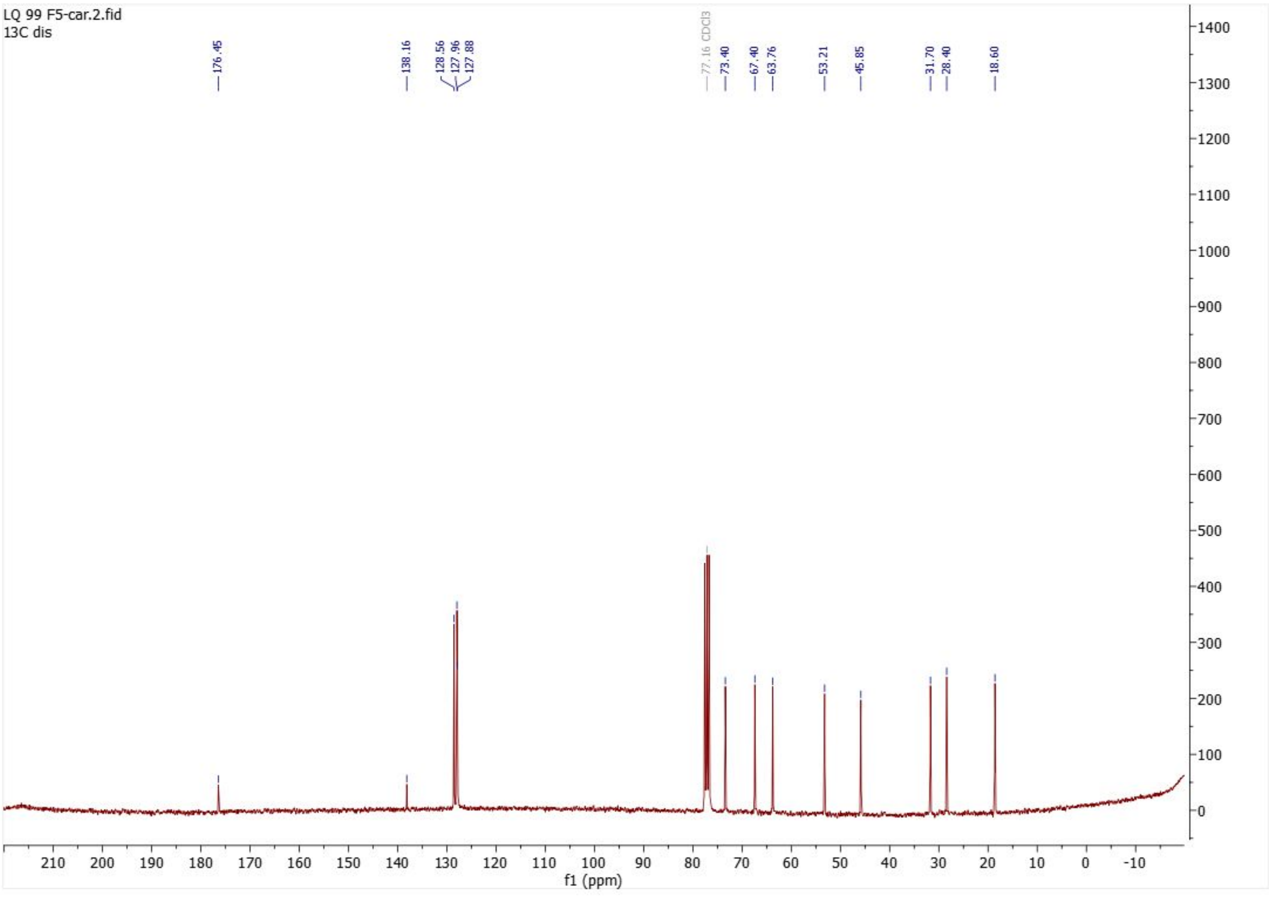

(*S*)-1-(1-hydroxybutan-2-yl)piperidin-2-one (4ab) ^{1}H NMR (300 MHz, $CDCl_3$); ^{13}C NMR (75 MHz, $CDCl_3$).

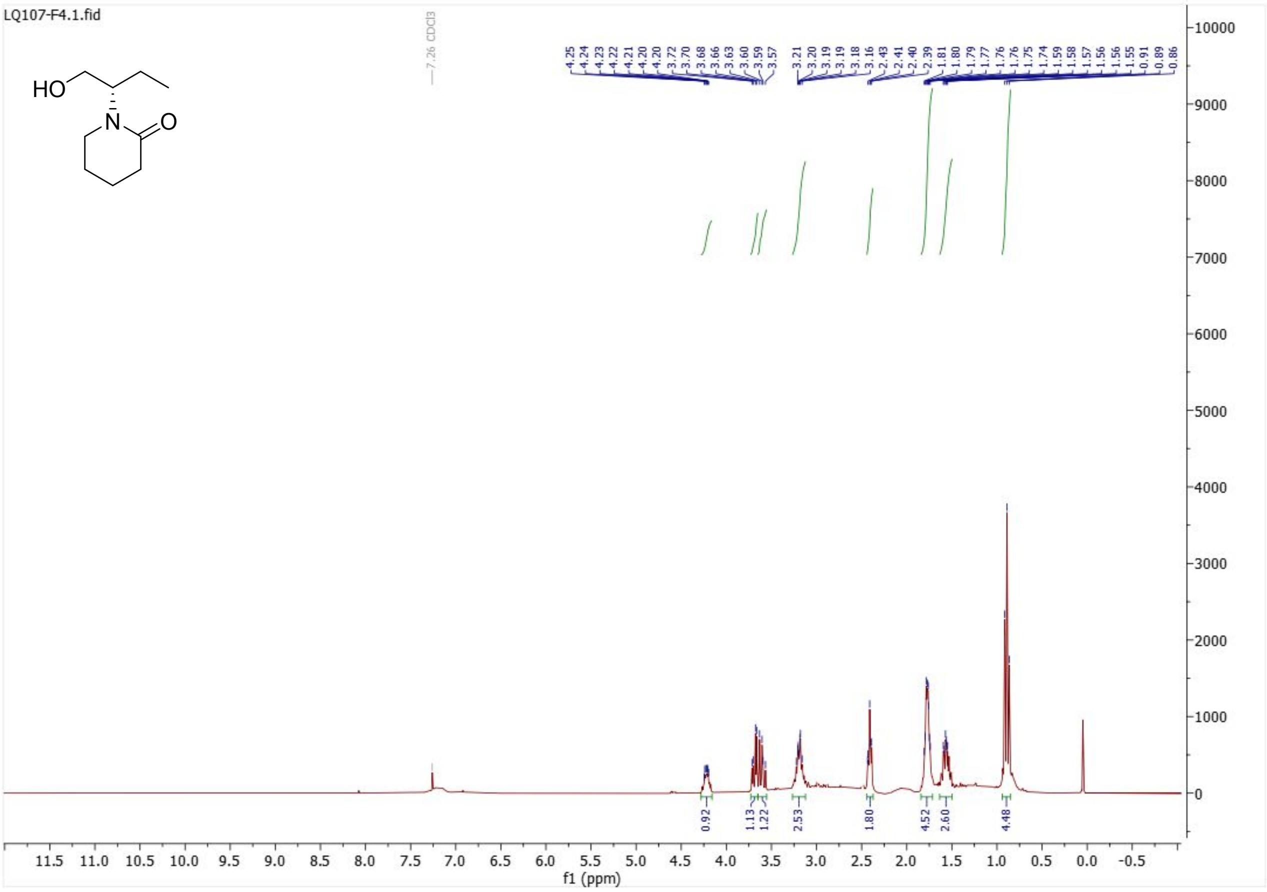

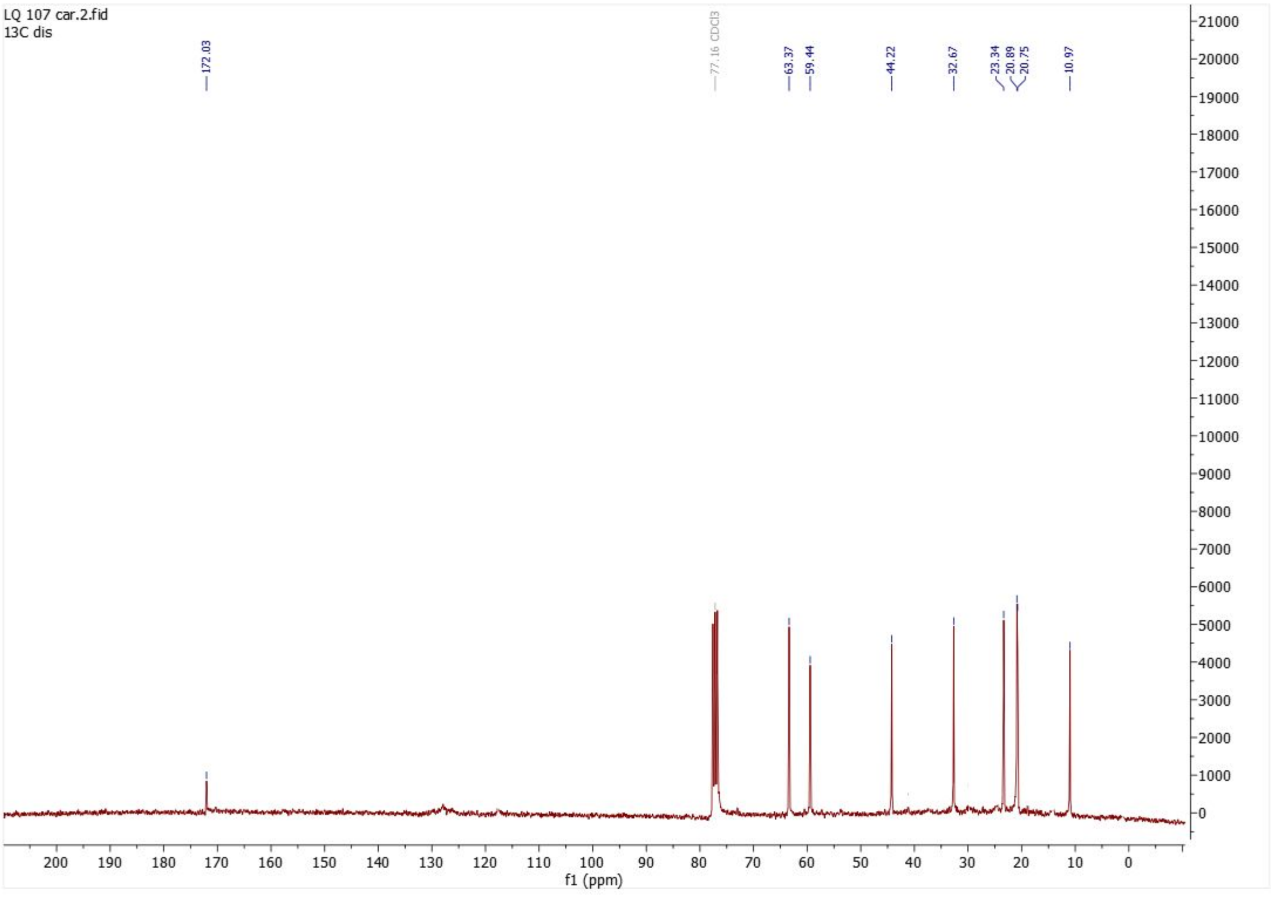

(*S*)-2-(2-oxopiperidin-1-yl)butyl 2-naphthoate ^{1}H NMR (300 MHz, $CDCl_3$); ^{13}C NMR (75 MHz, $CDCl_3$).

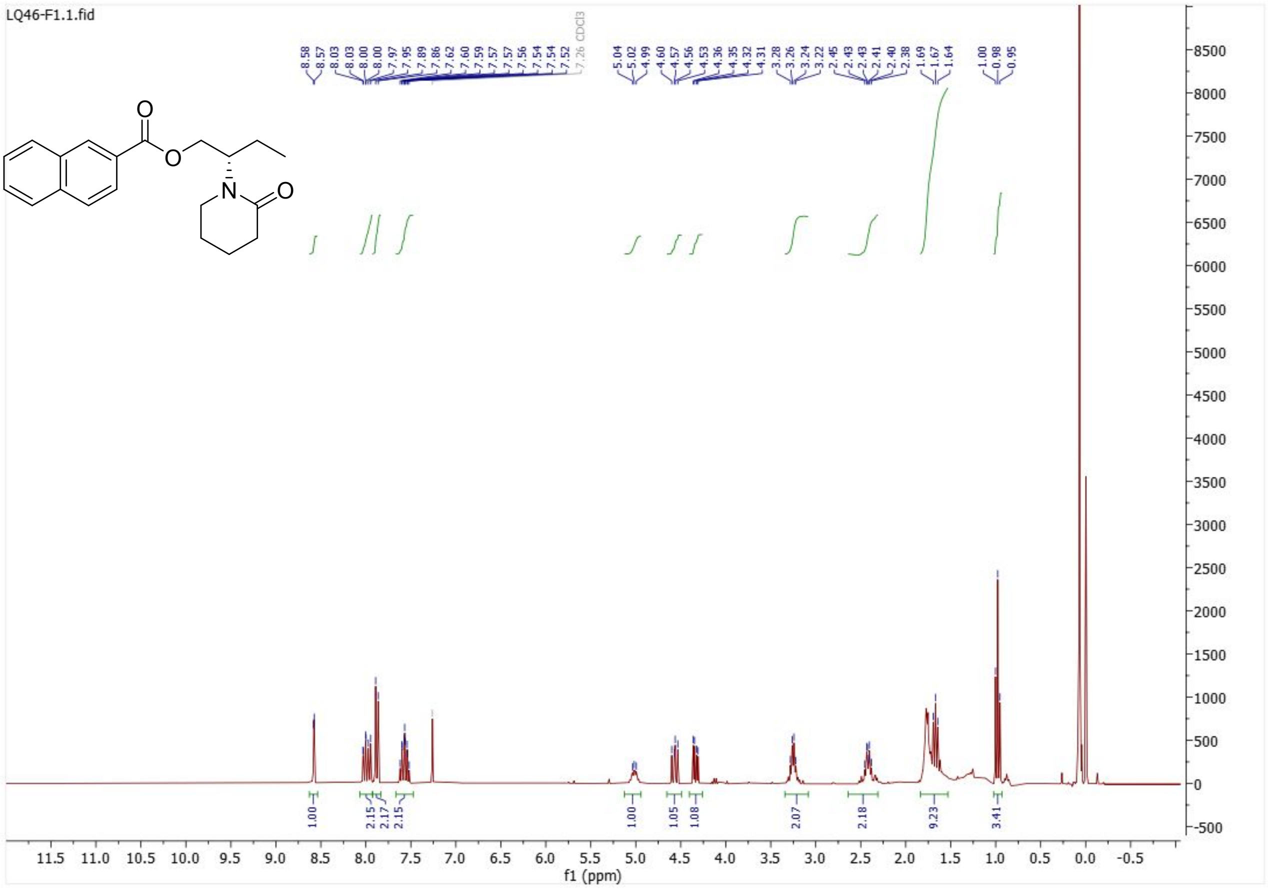

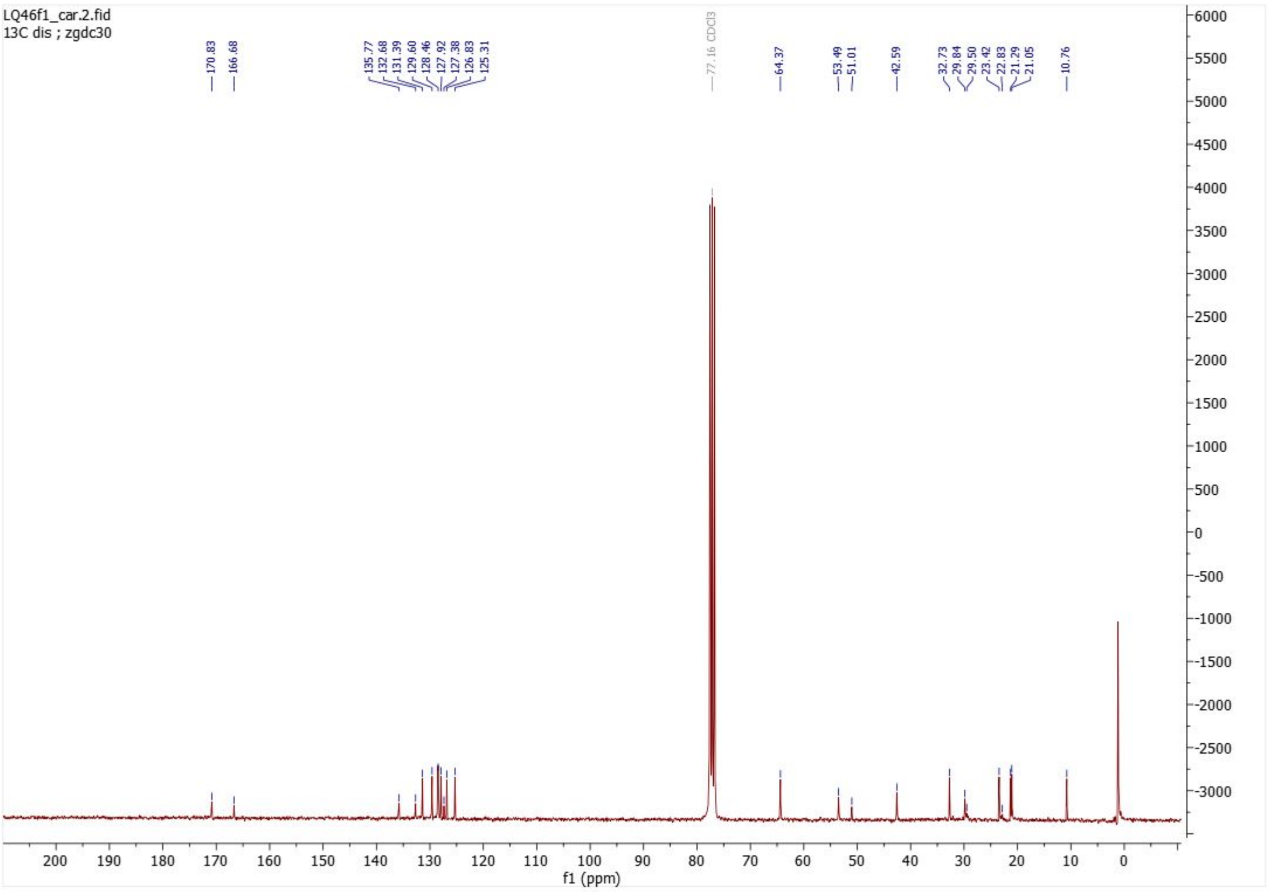

(*S*)-1-(1-hydroxybutan-2-yl)azepan-2-one (4ac) ^{1}H NMR (300 MHz, $CDCl_3$); ^{13}C NMR (75 MHz, $CDCl_3$).

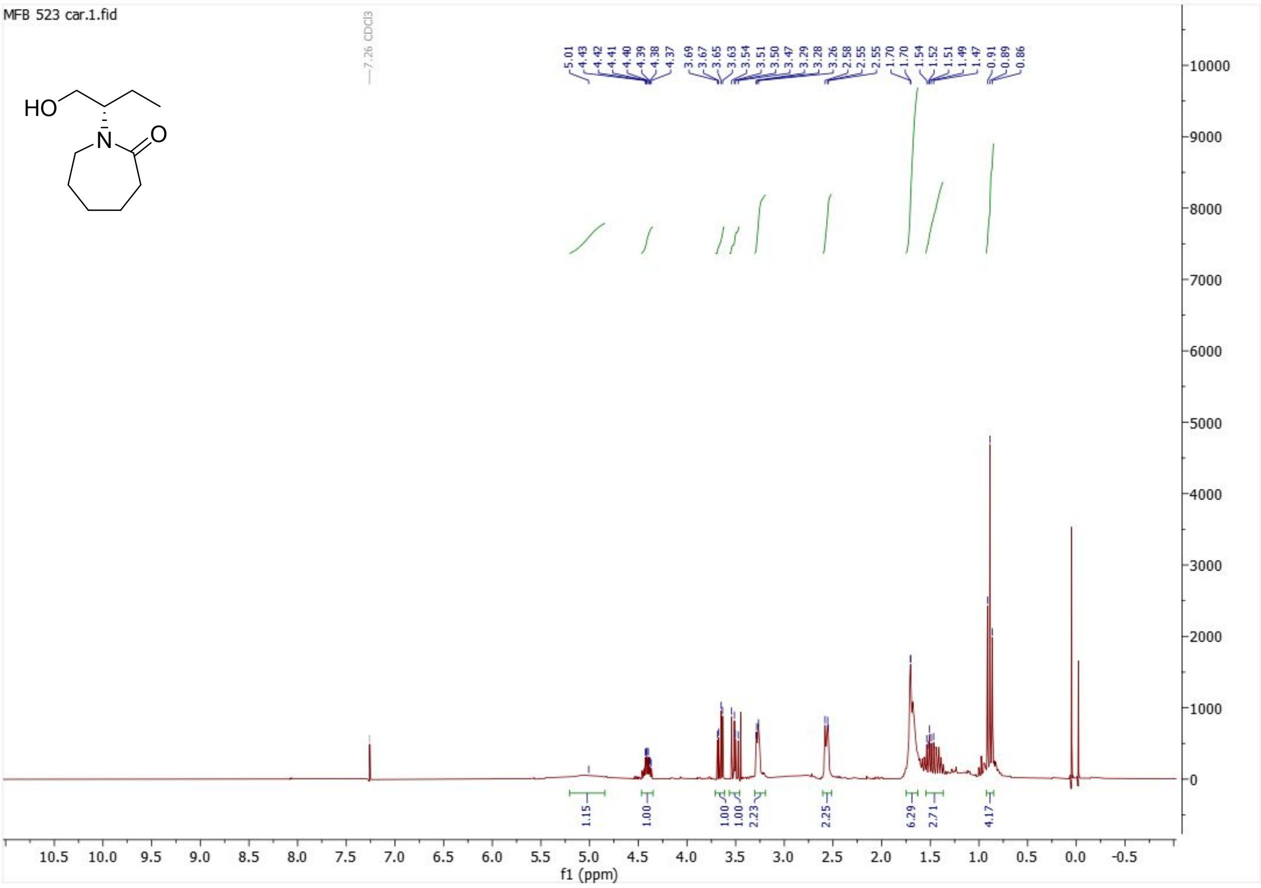

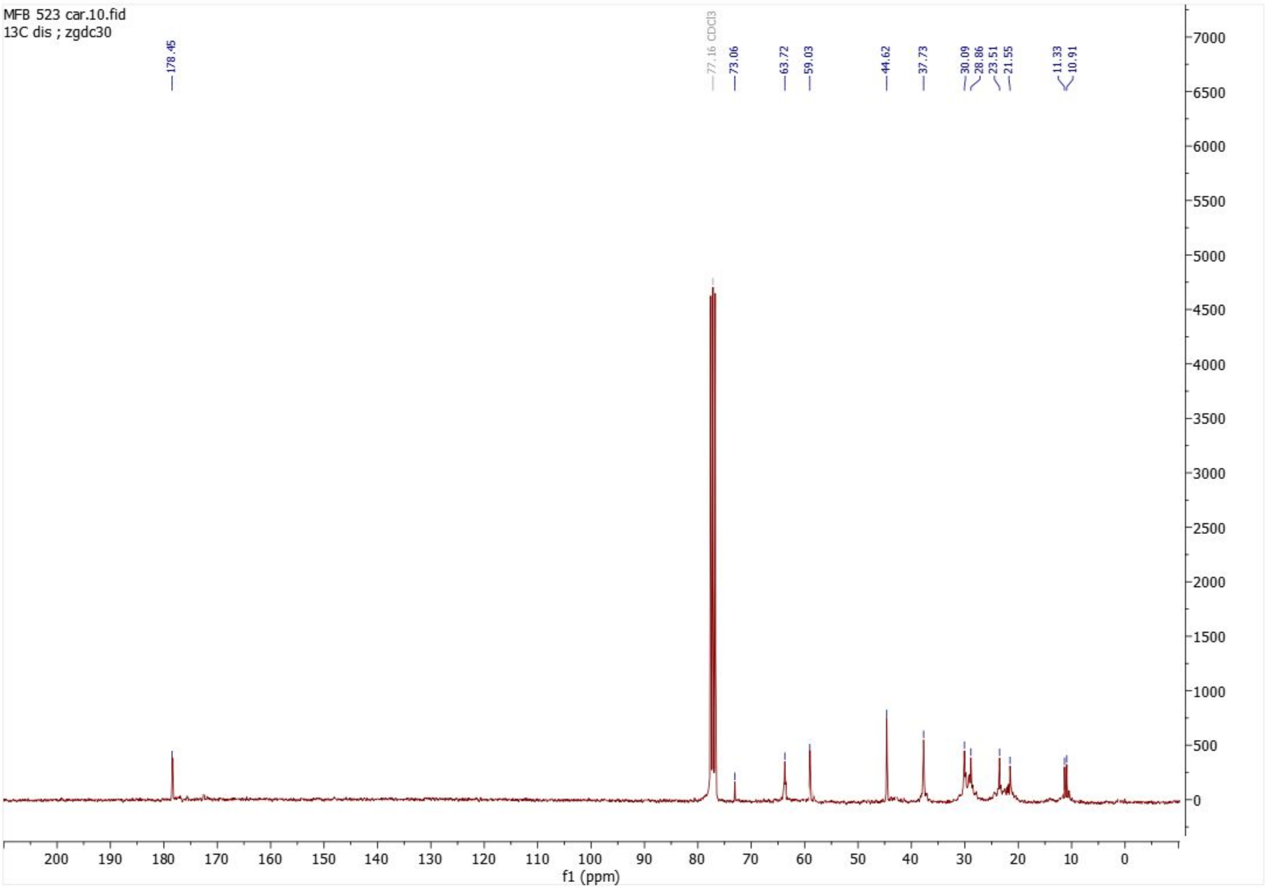

(*S*)-2-(2-oxoazepan-1-yl)butyl 2-naphthoate ^{1}H NMR (300 MHz, $CDCl_3$); ^{13}C NMR (75 MHz, $CDCl_3$).

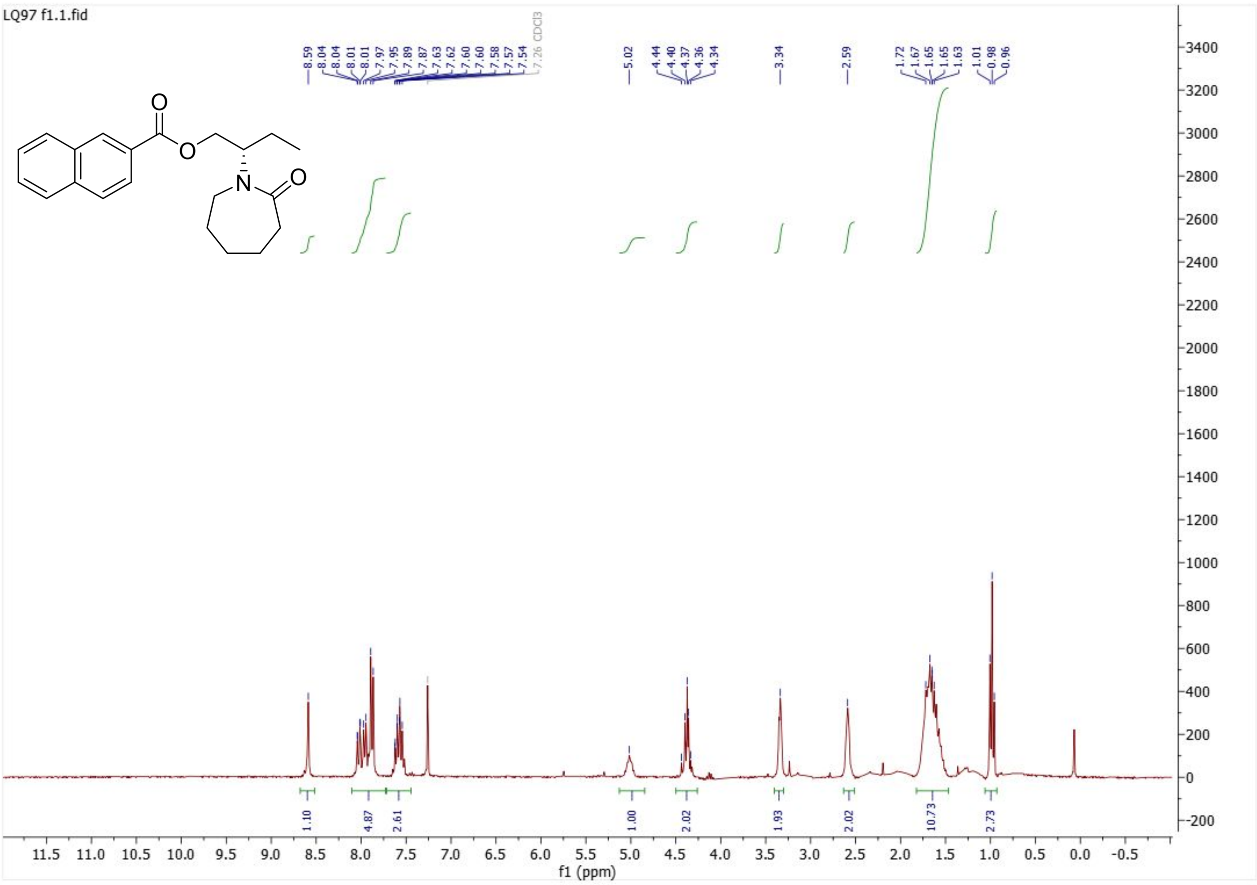

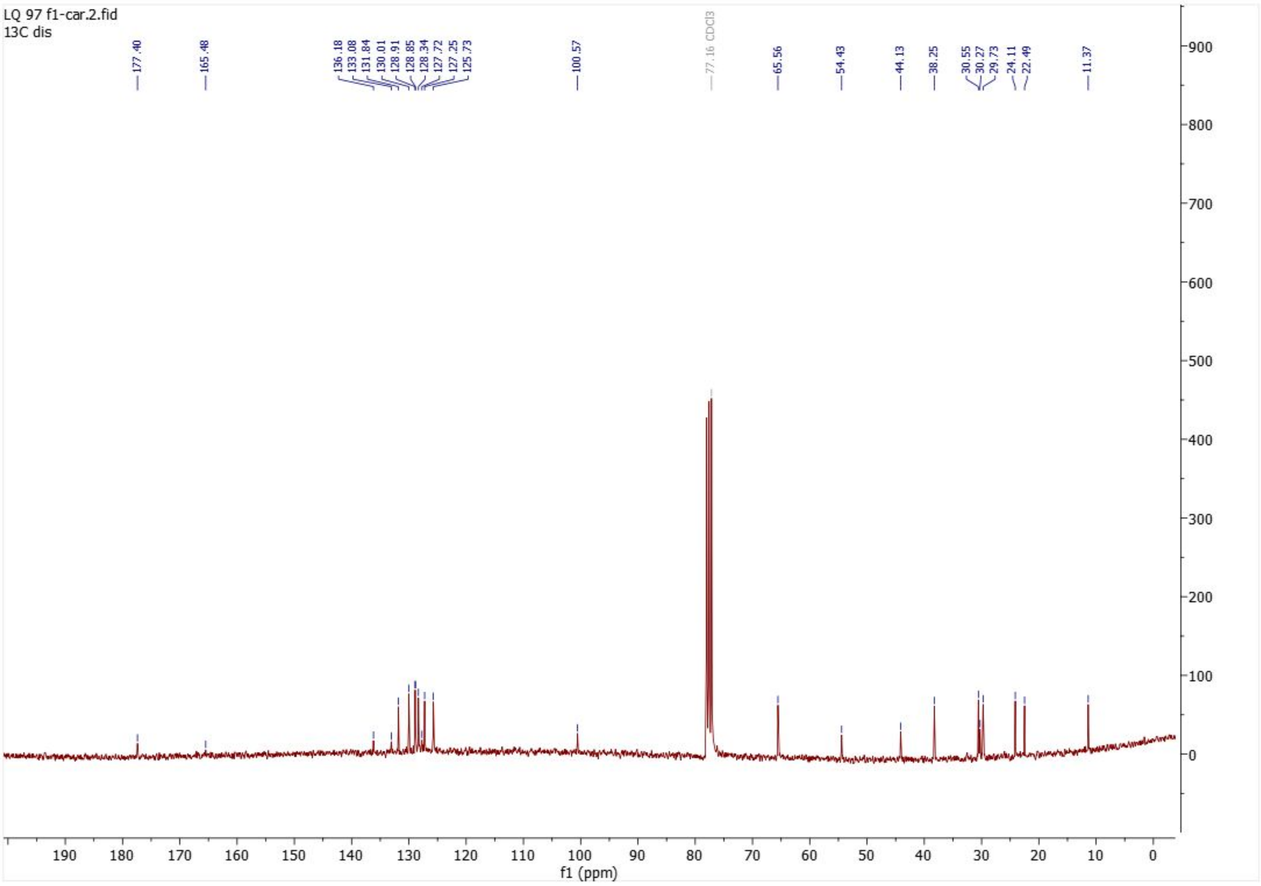

Levetiracetam (5) ^{1}H NMR (300 MHz, $CDCl_3$); ^{13}C NMR (75 MHz, $CDCl_3$).

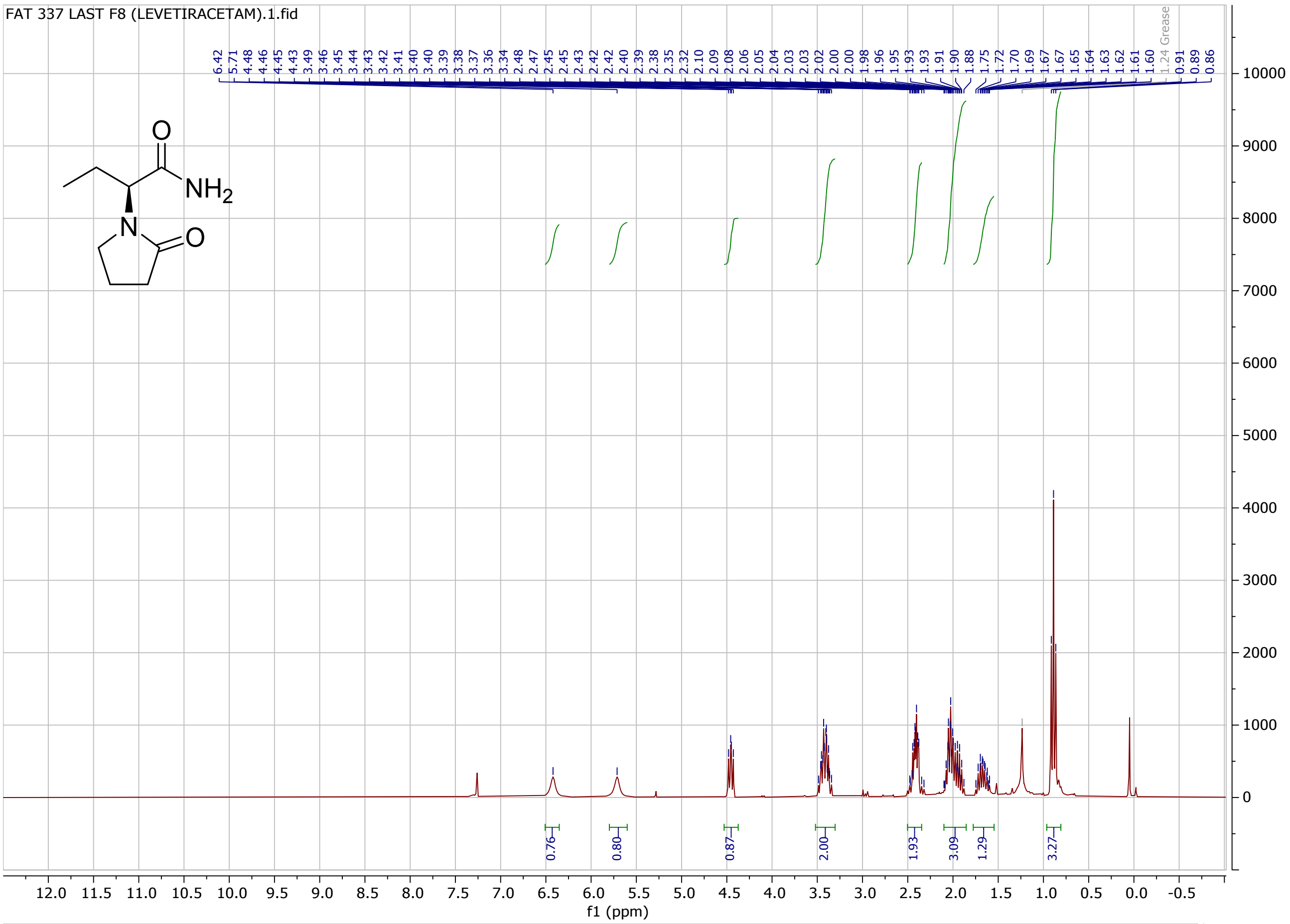

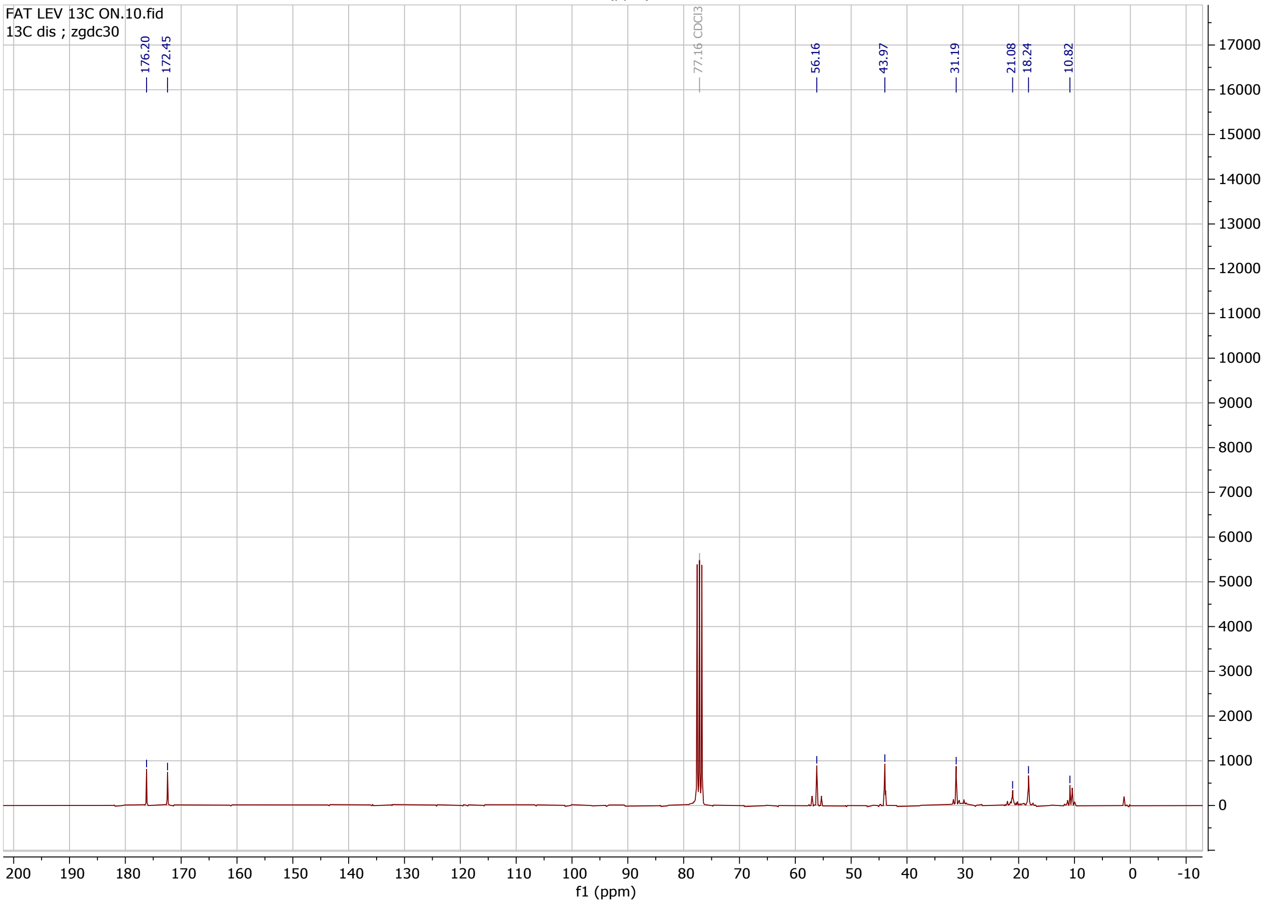

14 HPLC Traces

(*S*)-2-(2-oxopyrrolidin-1-yl)butyl 2-naphthoate

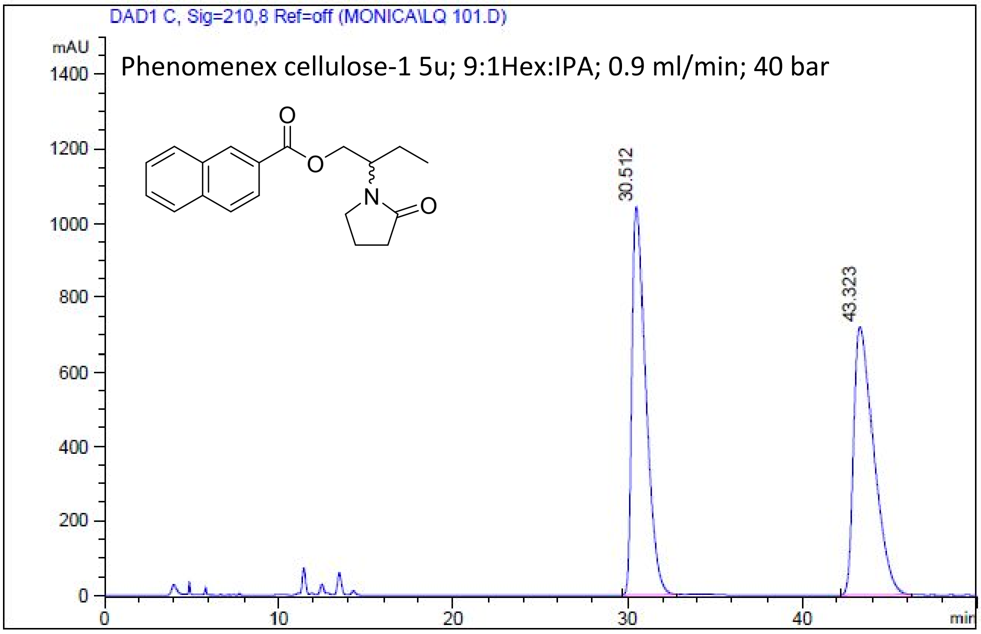

Signal 1: DAD1 C, Sig=210,8 Ref=off

Peak #	RT [min]	Type	Width [min]	Area	Area %	Name
1	30.512	BB	0.809	57049.754	49.812	
2	43.323	BB	1.121	57480.332	50.188	

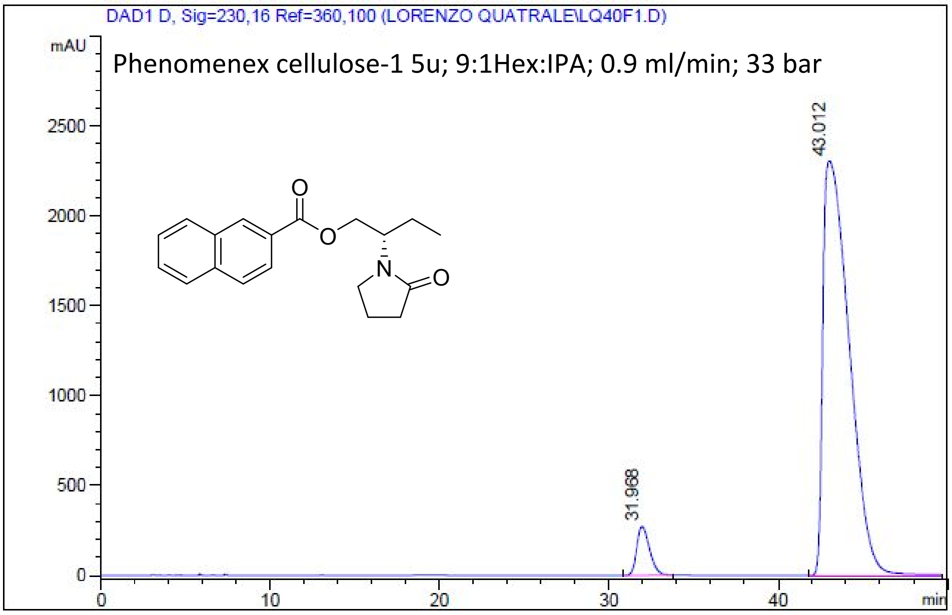

Peak #	RT [min]	Type	Width [min]	Area	Area %
1	31.968	BB	0.806	14155.285	5.392
2	43.012	BBA	1.412	248353.188	94.608

(S)-3-methyl-2-(2-oxopyrrolidin-1-yl)butyl 2-naphthoate

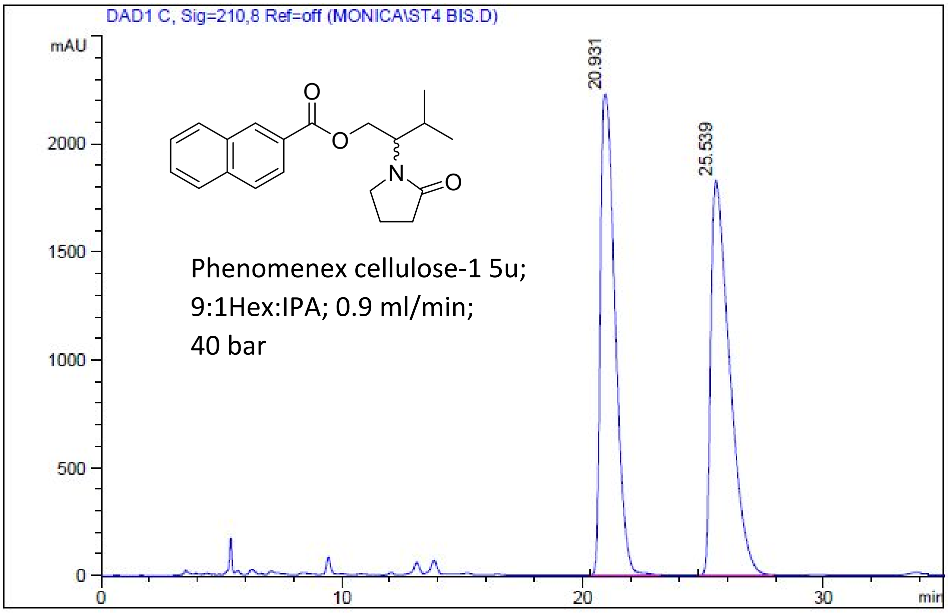

Signal 1: DAD1 C, Sig=210,8 Ref=off

Peak #	RT [min]	Type	Width [min]	Area	Area %	Name
1	20.931	BB	0.662	94580.008	48.550	
2	25.539	BB	0.831	100231.039	51.450	

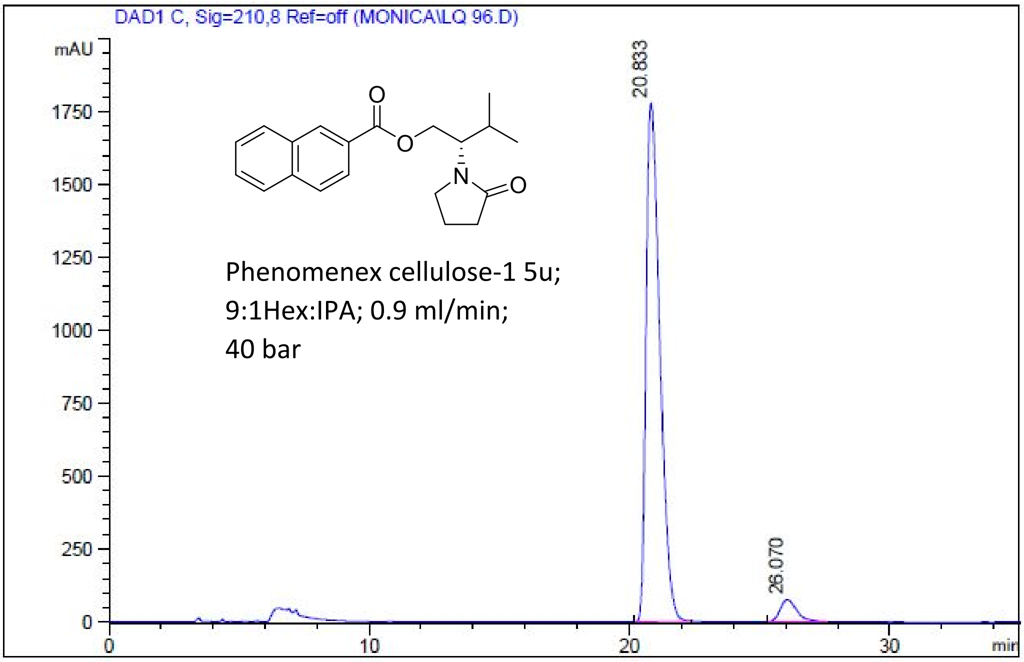

Signal 1: DAD1 C, Sig=210,8 Ref=off

Peak #	RT [min]	Type	Width [min]	Area	Area %	Name
1	20.833	BB	0.512	65647.555	94.895	
2	26.070	BB	0.656	3531.782	5.105	

(*S*)-1-(1-hydroxy-3-phenylpropan-2-yl)pyrrolidin-2-one (4da)

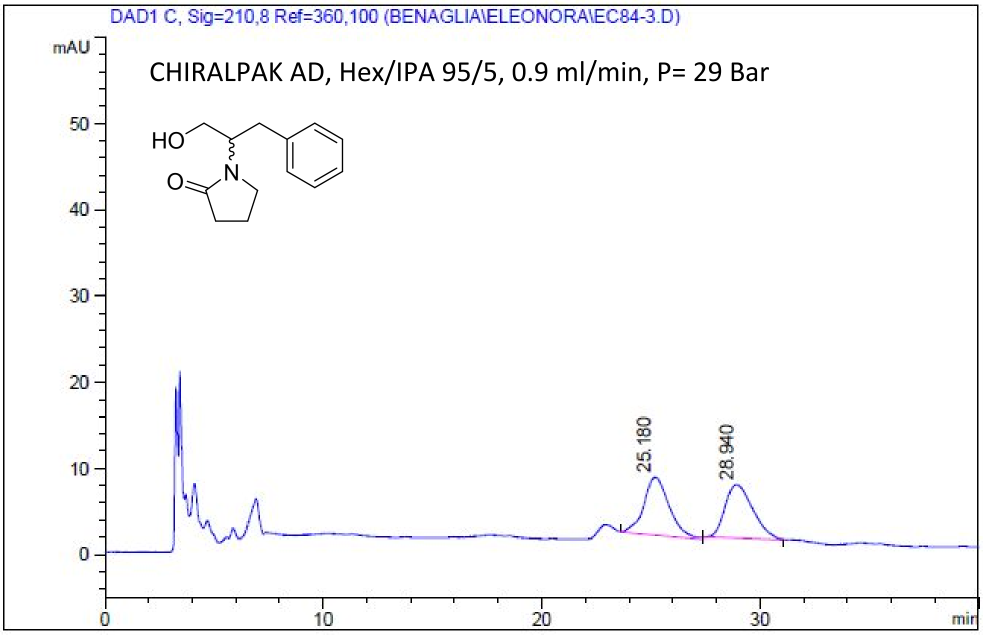

Peak #	RT [min]	Type	Width [min]	Area	Area %	Name
1	25.180	MM	1.341	541.071	49.709	
2	28.940	MM	1.473	547.415	50.291	

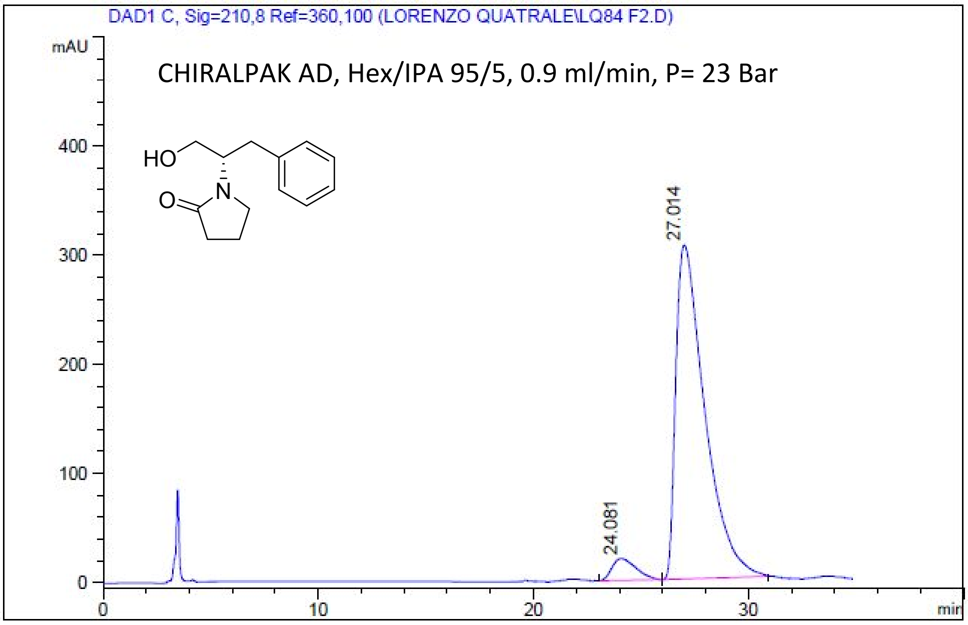

Peak #	RT [min]	Type	Width [min]	Area	Area %
1	24.081	VV	0.917	1558.747	5.169
2	27.014	VB	1.272	28594.707	94.831

(*S*)-3-(3,4-dimethoxyphenyl)-2-(2-oxopyrrolidin-1-yl)propyl 1 naphthoate

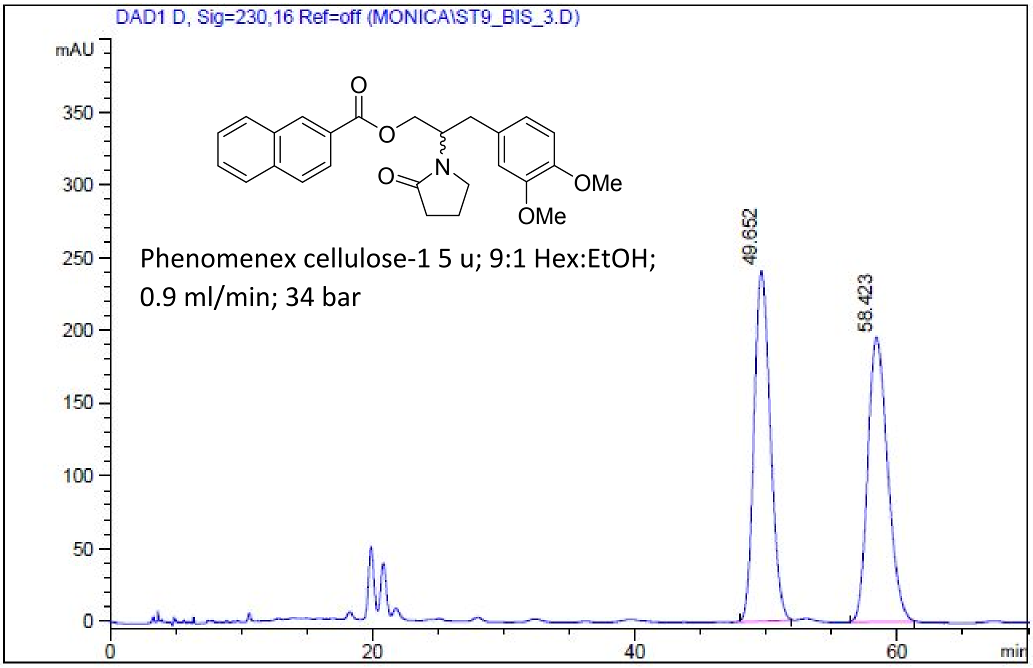

Peak #	RT [min]	Type	Width [min]	Area	Area %	Name
1	49.652	BB	1.339	20710.807	50.024	
2	58.423	BB	1.617	20690.988	49.976	

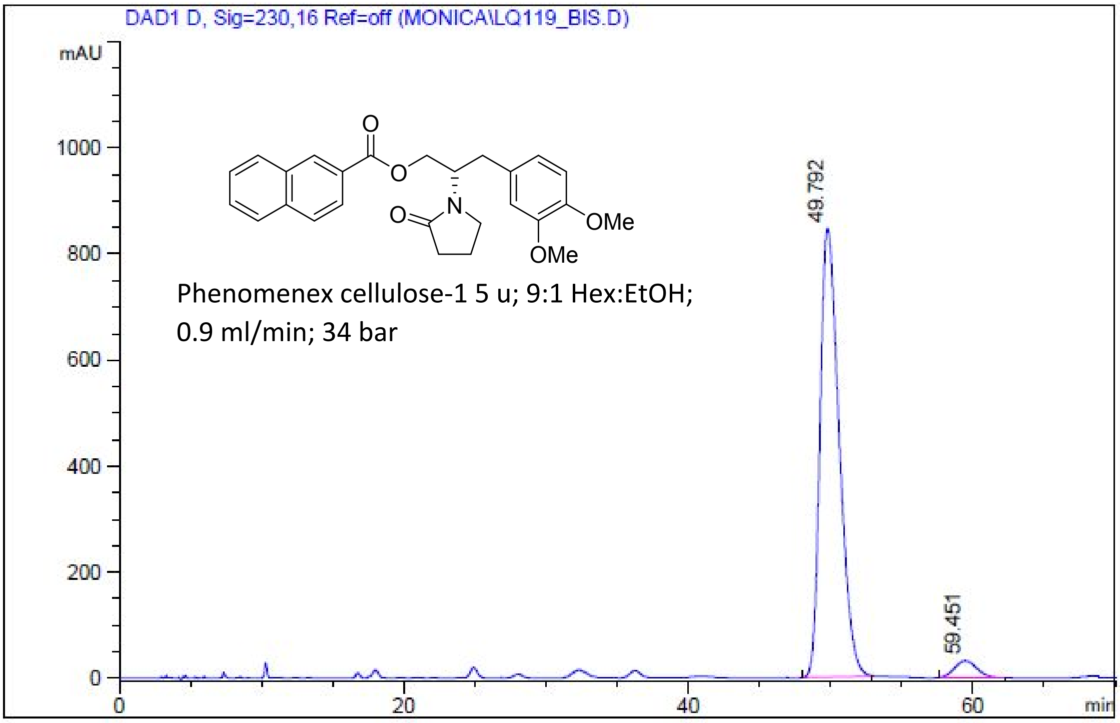

Peak #	RT [min]	Type	Width [min]	Area	Area %	Name
1	49.792	BB	1.425	78752.945	95.795	
2	59.451	BB	1.383	3456.821	4.205	

(S) 4-(benzyloxy)-1-hydroxybutan-2-yl)pyrrolidine-2-one (4fa)

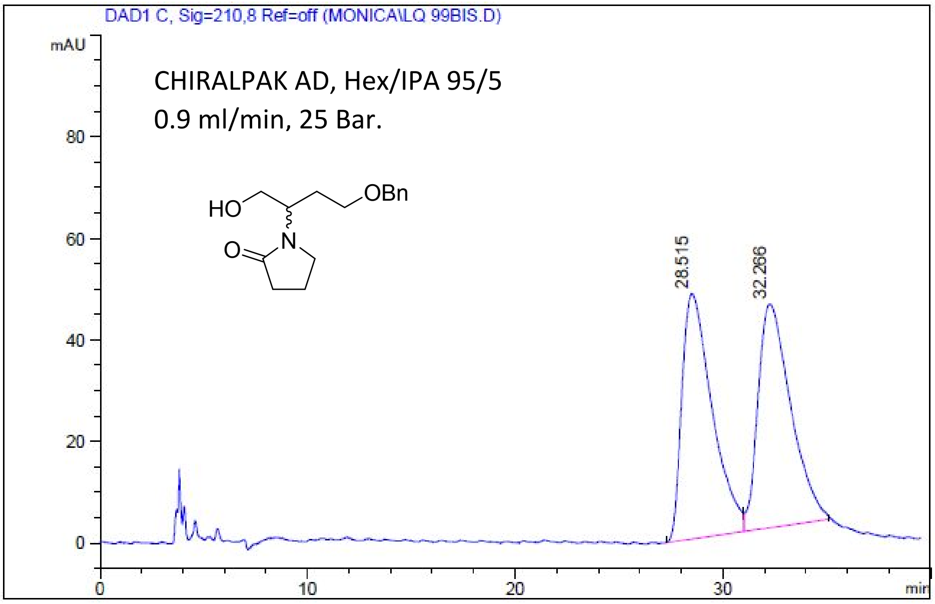

Signal 1: DAD1 C, Sig=210,8 Ref=off

Peak #	RT [min]	Type	Width [min]	Area	Area %	Name
1	28.515	BV	1.192	4877.710	50.241	
2	32.266	VB	1.284	4831.007	49.759	

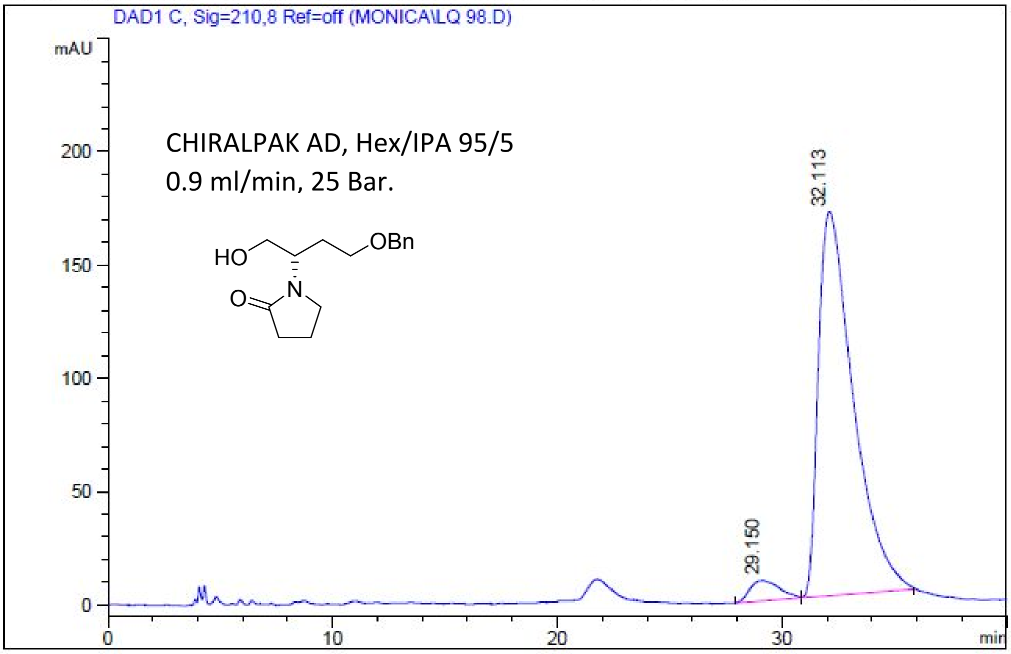

Signal 1: DAD1 C, Sig=210,8 Ref=off

Peak #	RT [min]	Type	Width [min]	Area	Area %	Name
1	29.150	BV	1.060	808.960	4.226	
2	32.113	VB	1.268	18332.719	95.774	

(*S*)-2-(2-oxopyrrolidin-1-yl)undec-10-en-1-yl 2-naphthoate

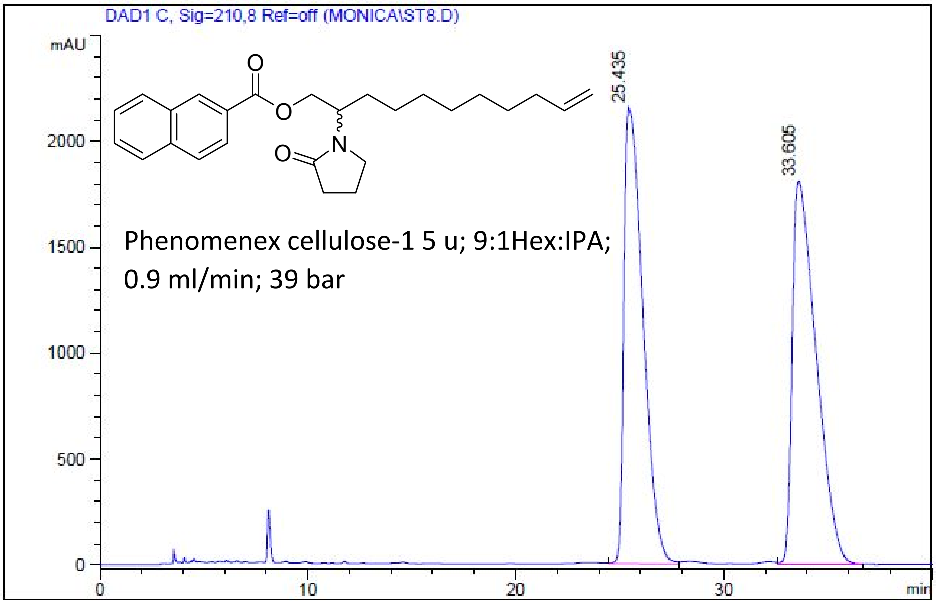

Signal 1: DAD1 C, Sig=210,8 Ref=off

Peak #	RT [min]	Type	Width [min]	Area	Area %	Name
1	25.435	VB	0.758	137733.578	48.748	
2	33.605	VB	0.948	144811.219	51.252	

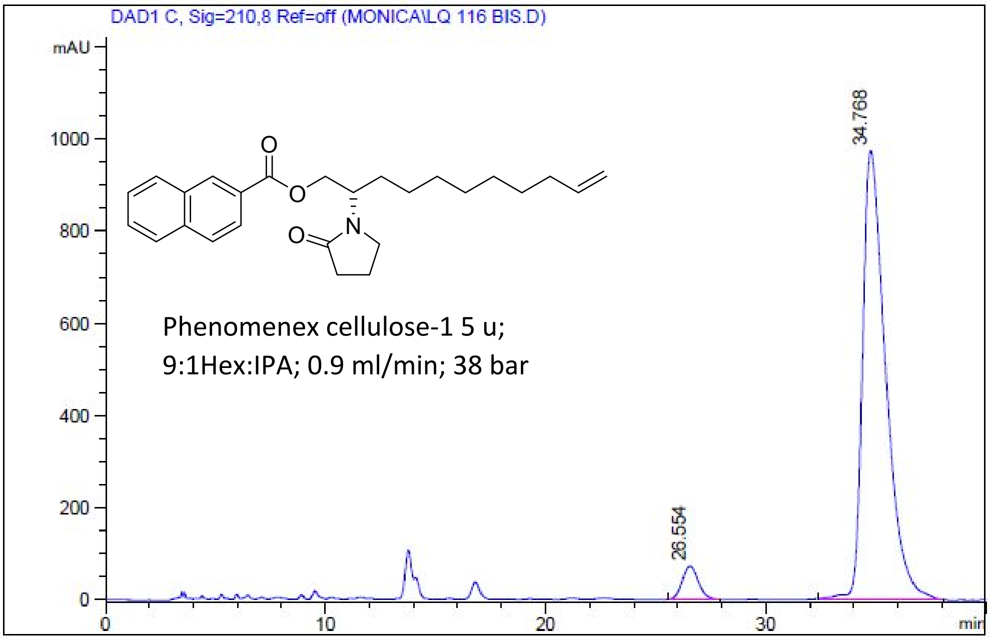

Signal 1: DAD1 C, Sig=210,8 Ref=off

Peak #	RT [min]	Type	Width [min]	Area	Area %	Name
1	26.554	BB	0.772	3730.410	4.951	
2	34.768	BB	1.054	71620.063	95.049	

(*S*)-2-(2-oxopiperidin-1-yl)butyl 2-naphthoate

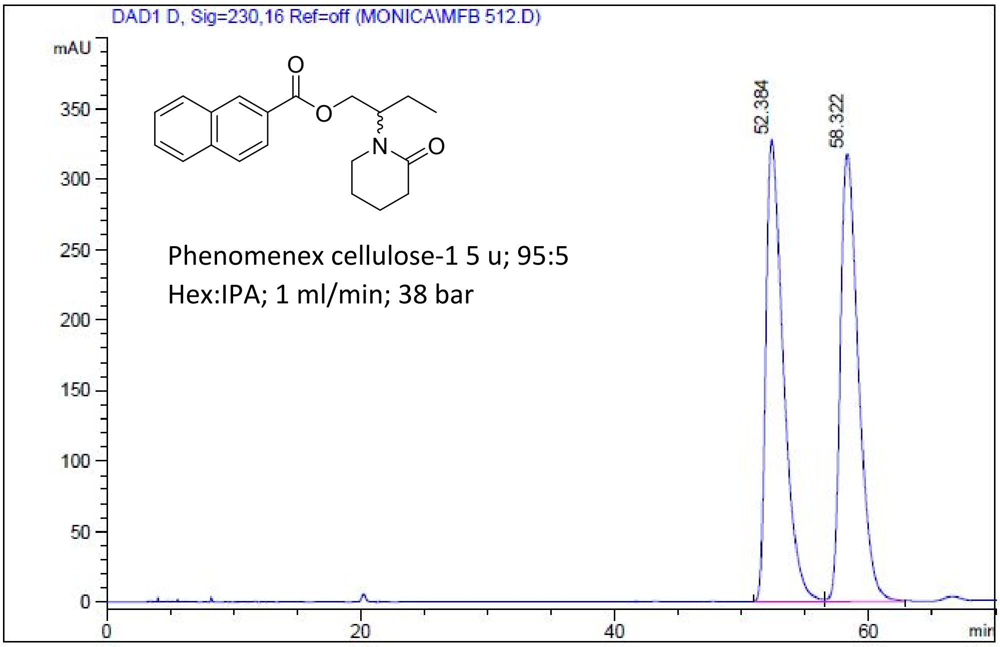

Signal 1: DAD1 D, Sig=230,16 Ref=off

Peak #	RT [min]	Type	Width [min]	Area	Area %	Name
1	52.384	BV	1.440	31365.445	49.974	
2	58.322	VB	1.507	31398.520	50.026	

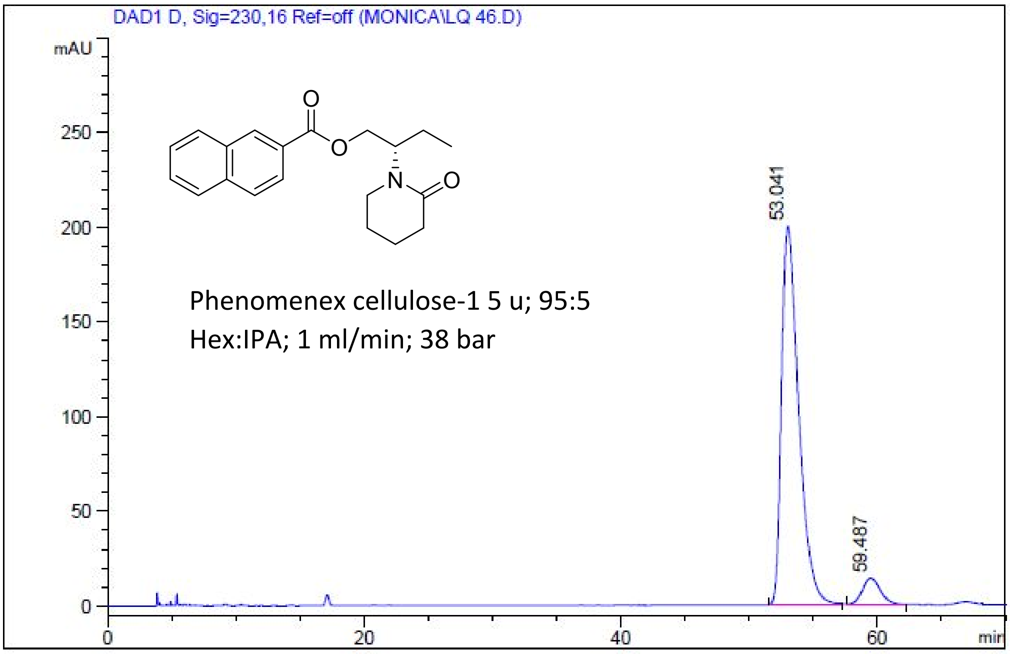

Signal 1: DAD1 D, Sig=230,16 Ref=off

Peak #	RT [min]	Type	Width [min]	Area	Area %	Name
1	53.041	BB	1.405	18599.738	92.993	
2	59.487	BB	1.529	1401.425	7.007	

(*S*)-2-(2-oxoazepan-1-yl)butyl 2-naphthoate

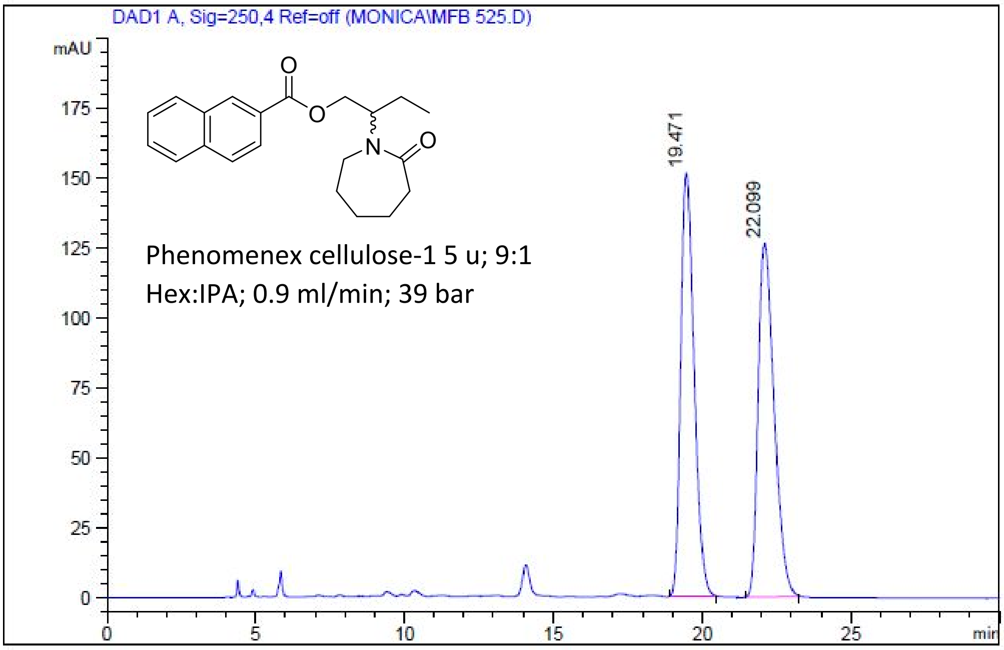

Peak #	RT [min]	Type	Width [min]	Area	Area %	Name
1	19.471	BB	0.483	4716.082	49.888	
2	22.099	BB	0.577	4737.182	50.112	

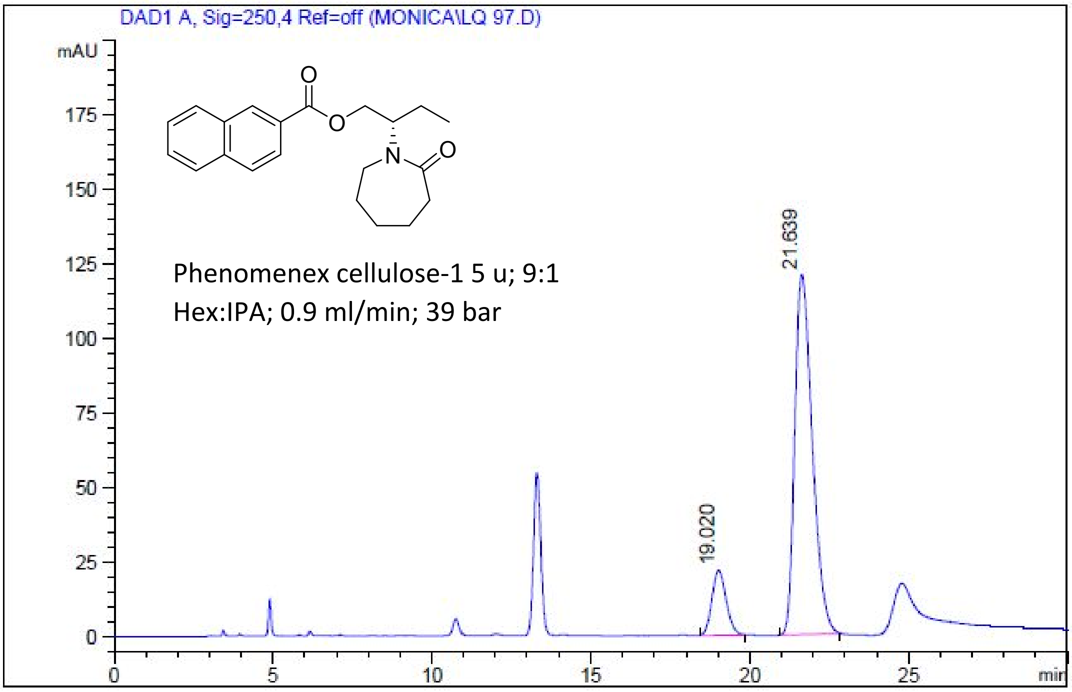

Peak #	RT [min]	Type	Width [min]	Area	Area %	Name
1	19.020	BB	0.468	681.054	12.801	
2	21.639	BB	0.588	4639.389	87.199	

Levetiracetam (5)

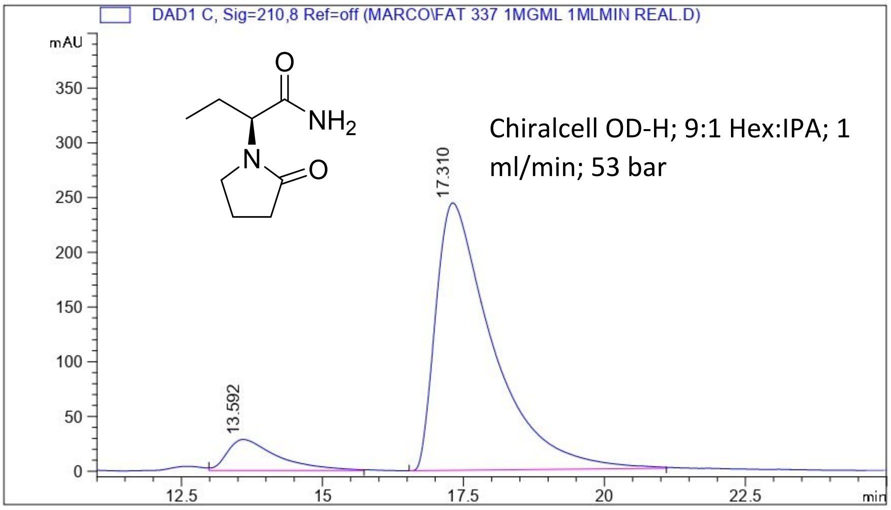

Signal 1: DAD1 C, Sig=210,8 Ref=off

Peak #	RT [min]	Type	Width [min]	Area	Area %	Name
1	13.592	VB	0.907	1752.594	9.237	
2	17.310	BB	1.019	17220.166	90.763	

15 References

[1] F. Herbrik, M. Sanz, A. Puglisi, S. Rossi, M. Benaglia, *Chem. – Eur. J.* **2022**, *28*, e202200164.

[2] T. W. Greulich, C. G. Daniliuc, A. Studer, *Org. Lett.* **2015**, *17*, 254–257.

[3] M. F. Boselli, I. Ghosh, N. Intini, M. Fattalini, A. Puglisi, B. König, M. Benaglia, *Chem. – Eur. J.* **2025**, *31*, e202404385.

[4] A. Hick, B. Gobert, E. Riguet, *Polyaromatic Urea Derivatives and Their Use in the Treatment of Muscle Diseases*, **2023**, US20230089368A1.

[5] J. B. Brazier, T. J. K. Gibbs, J. H. Rowley, L. Samulis, S. C. Yau, A. R. Kennedy, J. A. Platts, N. C. O. Tomkinson, *Org. Biomol. Chem.* **2015**, *13*, 133–141.

[6] L. Samulis, N. C. O. Tomkinson, *Tetrahedron* **2011**, *67*, 4263–4267.

[7] P. Torres, M. Guillén, M. Escribà, J. Crusats, A. Moyano, *Molecules* **2023**, *28*, 1997.

[8] T. Pecchioli, M. K. Muthyala, R. Haag, M. Christmann, *Beilstein J. Org. Chem.* **2015**, *11*, 730–738.

[9] M. C. Holland, J. B. Metternich, C. Daniliuc, W. B. Schweizer, R. Gilmour, *Chem. – Eur. J.* **2015**, *21*, 10031–10038.

[10] A. Pernet-Poil-Chevrier, F. Cantagrel, K. L. Jeune, C. Philouze, P. Y. Chavant, *Tetrahedron Asymmetry* **2006**, *17*, 1969–1974.

[11] E. Speckmeier, T. G. Fischer, K. Zeitler, *J. Am. Chem. Soc.* **2018**, *140*, 15353–15365.

[12] J. J. Kiddle, D. L. C. Green, C. M. Thompson, *Tetrahedron* **1995**, *51*, 2851–2864.

[13] M. Amatore, T. D. Beeson, S. P. Brown, D. W. C. MacMillan, *Angew. Chem. Int. Ed.* **2009**, *48*, 5121–5124.

[14] D. K. Arriaga, S. Kang, A. A. Thomas, *J. Org. Chem.* **2023**, *88*, 13720–13726.

[15] X. Qin, T. Tzvetkov, X. Liu, D.-C. Lee, L. Yu, D. C. Jacobs, *J. Am. Chem. Soc.* **2004**, *126*, 13232–13233.

[16] M. Mujahid, P. Mujumdar, M. Sasikumar, S. S. Kunte, M. Muthukrishnan, *Tetrahedron Asymmetry* **2012**, *23*, 1512–1515.

[17] K. Murai, D. Endo, N. Kawashita, T. Takagi, H. Fujioka, *Chem. Pharm. Bull. (Tokyo)* **2015**, *63*, 245–247.

[18] T. D. Owens, A. J. Souers, J. A. Ellman, *J. Org. Chem.* **2003**, *68*, 3–10.

[19] P. L. Southwick, D. R. Christman, *J. Am. Chem. Soc.* **1952**, *74*, 1886–1891.
